# Supplementary material for: Which elements of hospital-based clinical decision support tools for the assessment and management of children with head injury can be adapted for use by paramedics in prehospital care? A systematic mapping review and narrative synthesis
Source: BMJ Open. 2024 Feb 13;14(2):e078363. doi: 10.1136/bmjopen-2023-078363 (PMC10868315; doi:10.1136/bmjopen-2023-078363)
Supplement: Supplementary data [file bmjopen-2023-078363supp001.pdf]

#	Query	Limiters/Expanders	Last Run Via	Results
S1	(("Clinical decision support tool" OR "Clinical decision tool" OR "Clinical decision rule" OR "Diagnostic accuracy tool" OR "Triage tool" OR "Hospital-based tool" OR CDRs OR Intervention OR "Clinical decision score" OR "Decision Support Technique*") AND ("Head injury" OR "Minor head injury" OR "Traumatic brain injury" OR "Head trauma" OR "Head wound" OR "Intracranial injury") AND (Children OR Child OR Pediatric OR Paediatric OR Baby OR Babies OR Infant OR Schoolchild* OR Adolescent OR Teenage* OR "Young person")) AND ("emergency department" OR "trauma center" OR ED OR "A&E" OR "accident and emergency" OR "Emergency Service, Hospital")	Search modes - Boolean/Phrase	Interface - EBSCOhost Research Databases<br>Search Screen - Advanced Search<br>Database - CINAHL Plus;AMED - The Allied and Complementary Medicine Database;MEDLINE;APA PsycInfo	895

https://exports.ebscohost.com/sdc/a60c5b63-e8cf-4ea9-909b-1c08e39cb914.zip

Link to full search strategy.
