# Supplementary material for: Which elements of hospital-based clinical decision support tools for the assessment and management of children with head injury can be adapted for use by paramedics in prehospital care? A systematic mapping review and narrative synthesis
Source: BMJ Open. 2024 Feb 13;14(2):e078363. doi: 10.1136/bmjopen-2023-078363 (PMC10868315; doi:10.1136/bmjopen-2023-078363)
Supplement: Supplementary data [file bmjopen-2023-078363supp002.pdf]

Supplementary Table 1. Categories in each tool

	Signs of Skull fracture	Scalp Haematoma	Vomiting	Neuro Deficit	Headache	LOC	AMS	GCS	Seizure	Severe mechanism of injury	Drowsy	Amnesia	Vertigo	Non accidental injury	Clotting impairment/ anticoagulated	Drug/ alcohol use	Patient alert and well	Age
PECARN (2-18)	YES	NO	YES	NO	YES	YES	YES	YES	NO	YES	YES	NO	NO	NO	NO	NO	NO	NO
PECARN (<2)	YES	YES	NO	NO	NO	YES	YES	YES	NO	YES	YES	NO	NO	NO	NO	NO	YES	YES
CHALICE	YES	YES	YES	YES	NO	YES	NO	YES	YES	YES	YES	YES	NO	YES	NO	NO	NO	NO
CATCH	YES	YES	NO	NO	YES	YES	YES	YES	NO	YES	NO	YES	NO	NO	NO	NO	NO	NO
NEXUS 2	YES	YES	YES	YES	NO	YES	YES	YES	NO	NO	YES	YES	NO	NO	YES	NO	NO	NO
HIDATq	YES	YES	YES	YES	NO	YES	YES	YES	YES	YES	YES	YES	NO	YES	YES	NO	YES	YES
PALCHACK	YES	YES	YES	NO	YES	NO	YES	NO	NO	NO	NO	NO	NO	NO	NO	NO	NO	NO
HAYDEL	NO	YES	YES	NO	YES	NO	NO	NO	YES	NO	NO	YES	NO	NO	NO	YES	NO	YES
ATABAKI	YES	YES	YES	YES	YES	YES	YES	YES	YES	YES	YES	YES	YES	NO	NO	YES	NO	YES
GREENES	YES	YES	NO	NO	NO	NO	NO	NO	NO	NO	NO	NO	NO	NO	NO	NO	NO	NO
KLEMETTI	YES	YES	NO	YES	NO	YES	YES	NO	NO	NO	NO	NO	YES	NO	NO	NO	NO	NO
QUAYLE	YES	NO	NO	YES	NO	YES	YES	NO	YES	NO	NO	NO	NO	NO	NO	NO	NO	NO
DIETRICH	NO	NO	YES	YES	YES	YES	NO	NO	YES	NO	NO	NO	NO	NO	NO	NO	NO	NO
GUZEL	YES	YES	NO	YES	YES	YES	NO	NO	YES	YES	NO	YES	NO	NO	NO	NO	NO	NO
PredAHT	NO	YES	NO	NO	NO	NO	NO	NO	YES	NO	NO	NO	NO	NO	NO	NO	NO	NO
CHIDA	YES	NO	NO	NO	NO	NO	NO	YES	NO	NO	NO	NO	NO	NO	NO	NO	NO	NO
CIDSS2	YES	NO	NO	YES	NO	NO	NO	YES	YES	NO	NO	NO	NO	NO	NO	YES	NO	NO
PediBIRN	YES	YES	NO	NO	NO	NO	NO	NO	NO	NO	NO	NO	NO	NO	NO	NO	NO	NO
HEAD CT CHOICE	NO	NO	YES	YES	YES	NO	YES	YES	NO	NO	YES	NO	NO	NO	NO	NO	NO	NO
SNC GUIDELINE	YES	YES	YES	YES	YES	YES	YES	YES	YES	NO	NO	NO	NO	NO	YES	NO	NO	NO
BIG-1	YES	NO	NO	YES	NO	YES	NO	NO	NO	NO	NO	NO	NO	NO	NO	YES	NO	NO
NOVEL CDR	NO	NO	NO	NO	YES	YES	YES	YES	YES	YES	NO	NO	NO	NO	NO	NO	NO	NO
HEAD TRAUMA EBG	YES	YES	YES	YES	NO	YES	YES	YES	NO	YES	NO	NO	NO	NO	NO	NO	NO	YES
DA DALT	YES	NO	YES	YES	YES	YES	YES	YES	YES	NO	YES	YES	NO	NO	NO	NO	NO	NO
BUCHANIC	YES	YES	YES	YES	YES	NO	YES	NO	NO	NO	NO	NO	NO	NO	NO	NO	NO	NO
KIIDS-TBI CDS	YES	YES	NO	NO	NO	NO	NO	YES	NO	NO	NO	NO	NO	NO	NO	NO	NO	NO
