# Supplementary material for: Which elements of hospital-based clinical decision support tools for the assessment and management of children with head injury can be adapted for use by paramedics in prehospital care? A systematic mapping review and narrative synthesis
Source: BMJ Open. 2024 Feb 13;14(2):e078363. doi: 10.1136/bmjopen-2023-078363 (PMC10868315; doi:10.1136/bmjopen-2023-078363)
Supplement: Supplementary data [file bmjopen-2023-078363supp003.pdf]

Supplementary Table 2. Performance accuracy of each tool.

Name of tool	Internal validity	External validity	Implementation/ evaluation	Performance accuracy
**PECARN (under 2)**	Positive evidence	Positive evidence	Mixed evidence supporting potential effect.<br><br>Mixed evidence supporting positive conclusion for implementation.	Sensitivity: 100%<br><br>Specificity: between 53% and 60%
**PECARN (2-18)**	Positive evidence	Positive evidence	Mixed evidence supporting potential effect.<br><br>Mixed evidence supporting positive conclusion for implementation.	Sensitivity: 96.8%<br><br>Specificity: between 53% and 60%
**CHALICE**	Positive evidence	Positive evidence	Mixed evidence supporting positive conclusion for implementation.	Sensitivity: 98%<br><br>Specificity: 86%
**CATCH**	Positive evidence	Positive evidence	Not evaluated for usability, potential effect, or post implementation impact.	Sensitivity: 98%<br><br>Specificity: 50%
**NEXUS II**	Positive evidence	Mixed evidence supporting positive conclusion	Not evaluated for usability, potential effect, or post implementation impact.	Sensitivity: 99%<br><br>Specificity: 15%
**HIDATq**	Positive evidence	Not reported	Not evaluated in a published study after development	Negative predictive value for CT was 100% for intracranial injury. The

Supplementary Table 2. Performance accuracy of each tool.

				positive predictive value of the tool was low.
**PALCHACK**	Positive evidence	Positive evidence	Not evaluated in a published study after development	Sensitivity: 100%<br><br>Specificity: 46%
**HAYDEL**	Positive evidence	Not reported	Not evaluated in a published study after development	Sensitivity: 100%<br><br>Specificity: 24%
**ATABAKI**	Positive evidence	Not reported	Not evaluated in a published study after development	Sensitivity: 100%<br><br>Specificity: 46%
**BUCHANICH**	Positive evidence	Not reported	Not evaluated in a published study after development	Sensitivity: 100%<br><br>Specificity: 40%
**DA DALT**	Developed but not tested for internal validity	Not reported	Not evaluated in a published study after development	Sensitivity: 100%<br><br>Specificity: 87%
**GREENES**	Developed but not tested for internal validity	Not reported	Not evaluated in a published study after development	Sensitivity: 53%<br><br>Specificity: 72%
**KLEMETTI**	Developed but not tested for internal validity	Not reported	Not evaluated in a published study after development	Sensitivity: 94%<br><br>Specificity: 29%
**QUAYLE**	Developed but not tested for internal validity	Not reported	Not evaluated in a published study after development	Sensitivity: 44%<br><br>Specificity: 85%

Supplementary Table 2. Performance accuracy of each tool.

**DIETRICH**	Developed but not tested for internal validity	Not reported	Not evaluated in a published study after development	Sensitivity: 100%<br><br>Specificity: 17%
**GUZEL**	Developed but not tested for internal validity	Not reported	Not evaluated in a published study after development	Sensitivity: 69%<br><br>Specificity: 43%
**PredAHT 2**	Positive evidence	Positive evidence	Not evaluated for usability, potential effect,<br>or post implementation impact.	Sensitivity: 72%<br><br>Specificity: 86%
**CHIDA**	Negative evidence	Not reported	Not evaluated in a published study after development	Sensitivity: 94%<br><br>Specificity: 69%
**CIDSS2**	Positive evidence	Not reported	Not evaluated in a published study after development	Sensitivity: 51%<br><br>Specificity: 96%
**PediBIRN**	Positive evidence	Positive evidence	Not evaluated in a published study after development	Sensitivity: 96%<br><br>Specificity: 29%

Supplementary Table 2. Performance accuracy of each tool.

**Head CT Choice**	Positive evidence	Not reported	Not evaluated in a published study after development	Not reported
**SNC guideline**	Positive evidence	Positive evidence	Not evaluated in a published study after development	Sensitivity: 95%<br><br>Specificity: 59%
**BIG-1**	Positive evidence	Not reported	Not evaluated in a published study after development	Not reported
**Novel simplified CDR**	Positive evidence	Not reported	Not evaluated in a published study after development	Sensitivity: 80%<br><br>Specificity: 51%
**Head trauma EBG algorithm**	Positive evidence	Not reported	Not evaluated in a published study after development	Not reported
**KIIDS-TBI CDS**	Positive evidence	Positive evidence	Not evaluated in a published study after development	Sensitivity: 100%<br><br>Specificity: 26%

Supplementary Table 2. Performance accuracy of each tool.
