# Supplementary material for: Which elements of hospital-based clinical decision support tools for the assessment and management of children with head injury can be adapted for use by paramedics in prehospital care? A systematic mapping review and narrative synthesis
Source: BMJ Open. 2024 Feb 13;14(2):e078363. doi: 10.1136/bmjopen-2023-078363 (PMC10868315; doi:10.1136/bmjopen-2023-078363)
Supplement: Author's manuscript [file bmjopen-2023-078363.draft_revisions.pdf]

BMJ Open is committed to open peer review. As part of this commitment we make the peer review history of every article we publish publicly available.

When an article is published we post the peer reviewers' comments and the authors' responses online. We also post the versions of the paper that were used during peer review. These are the versions that the peer review comments apply to.

The versions of the paper that follow are the versions that were submitted during the peer review process. They are not the versions of record or the final published versions. They should not be cited or distributed as the published version of this manuscript.

BMJ Open is an open access journal and the full, final, typeset and author-corrected version of record of the manuscript is available on our site with no access controls, subscription charges or pay-per-view fees (http://bmjopen.bmj.com).

If you have any questions on BMJ Open's open peer review process please email info.bmjopen@bmj.com

BMJ Open

Which elements of hospital-based clinical decision support tools for the assessment and management of children with head injury can be adapted for use by paramedics in pre-hospital care? A systematic mapping review and narrative synthesis.

Journal:	*BMJ Open*
Manuscript ID	bmjopen-2023-078363
Article Type:	Original research
Date Submitted by the Author:	31-Jul-2023

Complete List of Authors:	Proctor, Alyesha; University of the West of England,<br>Lyttle, Mark; University of the West of England Bristol, Faculty of Health and Applied Sciences<br>Billing, Jedd; University of the West of England<br>Shaw, Pauline; University of the West of England<br>Simpson, Julian; University of the West of England<br>Voss, Sarah; University of the West of England, Health and Life Sciences<br>Benger, Jonathan; The University Hospitals NHS Foundation trust, Academic Department of Emergency care; The University of the West of England, Faculty of Health & Life Sciences

Keywords:	Systematic Review, PAEDIATRICS, ACCIDENT & EMERGENCY MEDICINE

SCHOLARONE™
Manuscripts

I, the Submitting Author has the right to grant and does grant on behalf of all authors of the Work (as defined in the below author licence), an exclusive licence and/or a non-exclusive licence for contributions from authors who are: i) UK Crown employees; ii) where BMJ has agreed a CC-BY licence shall apply, and/or iii) in accordance with the terms applicable for US Federal Government officers or employees acting as part of their official duties; on a worldwide, perpetual, irrevocable, royalty-free basis to BMJ Publishing Group Ltd ("BMJ") its licensees and where the relevant Journal is co-owned by BMJ to the co-owners of the Journal, to publish the Work in this journal and any other BMJ products and to exploit all rights, as set out in our licence.

The Submitting Author accepts and understands that any supply made under these terms is made by BMJ to the Submitting Author unless you are acting as an employee on behalf of your employer or a postgraduate student of an affiliated institution which is paying any applicable article publishing charge ("APC") for Open Access articles. Where the Submitting Author wishes to make the Work available on an Open Access basis (and intends to pay the relevant APC), the terms of reuse of such Open Access shall be governed by a Creative Commons licence – details of these licences and which Creative Commons licence will apply to this Work are set out in our licence referred to above.

Other than as permitted in any relevant BMJ Author's Self Archiving Policies, I confirm this Work has not been accepted for publication elsewhere, is not being considered for publication elsewhere and does not duplicate material already published. I confirm all authors consent to publication of this Work and authorise the granting of this licence.

For peer review only

TITLE:

Which elements of hospital-based clinical decision support tools for the assessment and management of children with head injury can be adapted for use by paramedics in pre-hospital care? A systematic mapping review and narrative synthesis.

AUTHORS:

Alyesha Proctor[1]

Dr Mark D Lyttle[1]

Jedd Billing[1]

Pauline Shaw[1]

Julian Simpson[1]

Professor Sarah Voss[1]

Professor Jonathan Benger[1]

[1]Faculty of Health and Applied Sciences, University of the West of England, Glenside Campus (1H30), Blackberry Hill, Bristol, BS16 1DD, England

Alyesha.proctor@uwe.ac.uk (NIHR Clinical Doctoral Research Fellow, Advanced Paramedic Practitioner)

Mark.Lyttle@uwe.ac.uk (Senior Research Fellow, Consultant in Paediatric Emergency Medicine)

Jedd.billing@uwe.ac.uk (Senior Lecturer in Paramedic Science)

Pauline.shaw@uwe.ac.uk (Subject Librarian)

Julian.simpson@uwe.ac.uk (Programme Lead, Senior Lecturer in Paramedic Science)

Sarah.voss@uwe.ac.uk (Professor of Emergency Care)

Jonathan.benger@uwe.ac.uk (Professor of Emergency Care, Consultant in Emergency Medicine)

*Corresponding author address: Alyesha Proctor, Glenside Campus, Blackberry Hill, Stapleton, Bristol, BS16 1DD.

ABSTRACT:

Background:

Hospital-based clinical decision tools support clinician decision making when a child presents to the Emergency Department with a head injury, particularly with regard to computed tomography scanning. However, there is no decision tool to support pre-hospital clinicians in deciding which head injured children require conveyance to hospital. This study aims to identify clinical decision tools, or constituent elements, which may be adapted for use in pre-hospital care to inform conveyance decisions.

Methods:

Systematic mapping review and narrative synthesis of published journal articles and grey literature. Searches were conducted using MEDLINE, EMBASE, PsycINFO, CINAHL and AMED. We systematically

For peer review only

identified all in-hospital clinical decision support tools and extracted from these the clinical criteria used in decision making. We complemented this with a narrative synthesis.

Results:

Following de-duplication 874 articles were identified. After screening titles and abstracts, 697 articles were excluded, leaving 177 full text articles. Of these, 95 were excluded, yielding 82 studies. A further 14 studies were identified in the literature after cross checking, totalling 96 analysed studies. 25 relevant in-hospital clinical decision tools were identified, encompassing 67 different clinical criteria, which grouped into 18 categories.

Conclusion:

Factors that increase the likelihood of neurosurgical intervention, and should be considered for use in a clinical decision tool designed to support paramedics in the assessment and management of children with head injury, are: signs of skull fracture; a large, boggy or non-frontal scalp haematoma (particularly in infants); focal neurological deficit; Glasgow Coma Scale score less than 15; prolonged or worsening headache; prolonged loss of consciousness; post traumatic seizure; amnesia in older children; non-accidental injury; drug or alcohol use; less than one year old. Clinical criteria that require further investigation include mechanism of injury; clotting impairment/anticoagulation; vertigo; length of time of unconsciousness; number of vomits.

Strengths and limitations of the study:

- The review is highly inclusive, with a range of global study settings, including qualitative, quantitative, and mixed methods research.
- The review provided an opportunity to complete a detailed and contemporary search of all available sources and identified 25 in-hospital tools, compared to 14 in a previous systematic review.
- The role of mapping reviews is to provide a descriptive account of the published literature; therefore, this should be considered when assessing the findings of the overall evidence synthesis.
- A direct comparison of the clinical decision support tools is not possible because they address different questions, age groups, injury severities, and outcomes.

INTRODUCTION:

Healthcare systems internationally are under substantial pressure due to rising patient demand. A leading priority is to reduce the number of patients who are unnecessarily conveyed to an Emergency Department (ED) by ambulance [1]. Children frequently use the ambulance service and ED, and childhood head injury is common; approximately 800,000 head-injured children attend EDs each year in the UK [2]. Very few children with head injury who are conveyed to the ED by ambulance need specialist treatment, and the vast majority could be managed safely at scene. Despite this, a national overview of head injury in children found that one-third of children in hospital with head injury were transported by ambulance, and 74% of those conveyed were non-serious, requiring no intervention [3].

Head injury guidance produced by the National Institute for Health and Care Excellence (NICE) [4] is of limited utility to paramedics for reasons including: a lack of validation in pre-hospital care; reference to patient observation over time which is not always possible; challenges in the

For peer review only - http://bmjopen.bmj.com/site/about/guidelines.xhtml

assessment of amnesia in younger children; limited research evidence on which to base recommendations relating to ambulance non-conveyance [4].

There are several existing validated hospital-based decision support tools that clinicians use when a child presents to the ED with a minor head injury, and studies have demonstrated their effectiveness in aiding decision-making for both immediate discharge and computed tomography (CT) scanning [5] [6]. Currently, there are no pre-hospital decision tools designed to support paramedics in reducing unnecessary conveyance to the ED for head-injured children. None of the existing hospital-based tools can be implemented directly in pre-hospital care, since they are designed to support decision making on performing CT scanning, rather than ED conveyance.

AIM:

To systematically identify and examine clinical criteria within existing hospital-based clinical decision support tools for children with minor head injury, with a focus on determining their potential to aid decision making regarding hospital conveyance by paramedics.

METHODS:

Introduction:

Systematic mapping aims to collate, explain, and catalogue available evidence (primary and secondary) relating to a topic of interest, to identify a focus for more specific investigation [7]. It is a review methodology that is often used in healthcare research that aims to detail and categorise literature on a particular topic, with the intention of developing this into more comprehensive work [8]. This systematic mapping review was conducted in accordance with published methods [9] and is reported in line with the PRISMA statement [10]. It has not been registered with Prospero as it is a mapping review.

Patients and public have been involved throughout this research. Both a Young Persons Advisory Group (YPAG) (children aged 9-16 years) and Parent Advisory Group (PAG) have supported this study and confirmed its importance for children, parents, and the wider health and care system.

Search strategy:

Searches were conducted in the following databases, for articles published between January 1980 and August 2022 to ensure that all relevant contemporary research was included: MEDLINE, EMBASE, PsycINFO, CINAHL and AMED. A Google Scholar and a Web of Science search were undertaken to identify reports or proceedings not indexed in the above. A literature advisory group, including experts in the field, was consulted to identify relevant grey literature and unpublished reports. Furthermore, to find additional published, unpublished, and ongoing studies, the reference lists of all relevant studies were examined.

Search terms were developed iteratively by discussion among the research team, patient and public representatives and a librarian, seeking a balance between comprehensiveness and focus. A combination of MeSH terms and synonym text-strings/phrases were used in the search strategy and were combined using Boolean operators. Updated searches were re-run before the final analysis. The search terms used can be seen in Table 1.

The search was limited to English language studies due to resource restrictions and the cost of translation, and only articles with full texts available were included. Studies that may have been relevant but did not include or refer to a clinical decision support tool for the assessment and

For peer review only - http://bmjopen.bmj.com/site/about/guidelines.xhtml

management of head-injured children, were excluded; however, there were no restrictions on the types of study included. Eligibility criteria are presented in full in Table 2.

Table 1. Search terms:

Clinical decision support tool	Head injury	Children	Hospital
Clinical decision tool	Minor head injury	Child	ED
Clinical decision rule	TBI (Traumatic brain injury)	Pediatric	Emergency Department
Diagnostic accuracy tool	Head trauma	Paediatric	A+E
Triage tool	Head wound	Baby	Accident and Emergency
Hospital-based tool	Intracranial injury	Babies	Emergency room
CDRs	CiTBI (clinical important traumatic brain injury)	Infant	Trauma centre
Intervention	ICI (intracranial injury)	Schoolchild	
Clinical decision score	CSII (clinically significant intracranial injury)	Adolescent	
Paediatric head injury predictive tool	Trivial head injury	Teenager	
Clinical Prediction Rule		Young person	
Clinical Prediction Tool			
Clinical decision support tool (CDST)			

Table 2. Inclusion and exclusion criteria:

Inclusion Criteria	Exclusion Criteria
Studies in the English Language	Non- English language
Quantitative, qualitative, mixed methods research, systematic reviews	
Children (under 19 years of age)	Adult population (19 years or older)
Date of publication 1980-present	
Any study that includes a clinical decision support tool for assessing and managing children with head injury	Any study that did not include a clinical decision support tool for the assessment and management of head-injured children
International as long as in the English language	

Extracting, Coding, and Synthesising the Data:

Titles and abstracts were double screened for relevance and assessed for eligibility using Covidence software [11], following which full texts of included articles underwent review for eligibility. Any disagreements were resolved through discussion with a senior researcher. Reasons for exclusion

For peer review only - http://bmjopen.bmj.com/site/about/guidelines.xhtml

were documented, a topic expert reviewed the final set of included papers to ensure relevant studies and tools had not been overlooked. Figure 1 shows the PRISMA flow diagram [10].

Data extraction included systematically identifying the title of each tool, the aim, derivation reference, supporting literature, conclusion, and key points relating to the content and format of a clinical decision support tool for paramedic use. An inductive coding frame was developed to map emerging concepts, which involved deriving codes from the data. Key messages/concepts in relation to predicting conveyance from all studies were extracted from the methods, results, and conclusion sections. This resulted in a visual synthesis and classification of available studies and tools. The data extraction tool was piloted using two included studies, and data were extracted by hand and cross-checked by another researcher.

Clinical criteria used in decision making were extracted from all tools; a summary map was produced providing a visual display of clinical criteria included in each tool. After independently producing pilot categories based on a sample of clinical decision support tools, the research team formed consensus on categories. The frequency of each clinical criterion was tabulated, and performance accuracy was recorded where reported. A narrative synthesis was subsequently developed using this framework.

Assessment of Quality:

No formal assessment of quality assessment was undertaken, as this is not required in mapping reviews [12]. Data extraction in mapping reviews describes the studies and their main methods but does not attempt a full data extraction and quality assessment [12].

RESULTS:

A total of 874 articles were identified after duplicates were removed. After screening titles and abstracts, 697 articles were excluded, which left 177 full text articles to be assessed for eligibility. Of these, 95 were excluded for reasons including: adult population only; not including a clinical decision support tool; not in the English language; and full text not available. Three articles were not included, as they were conference abstracts. Fourteen additional studies were identified through citation searching, resulting in 96 included studies (Figure 1).

For peer review only - http://bmjopen.bmj.com/site/about/guidelines.xhtml

Figure 1. PRISMA flow diagram:

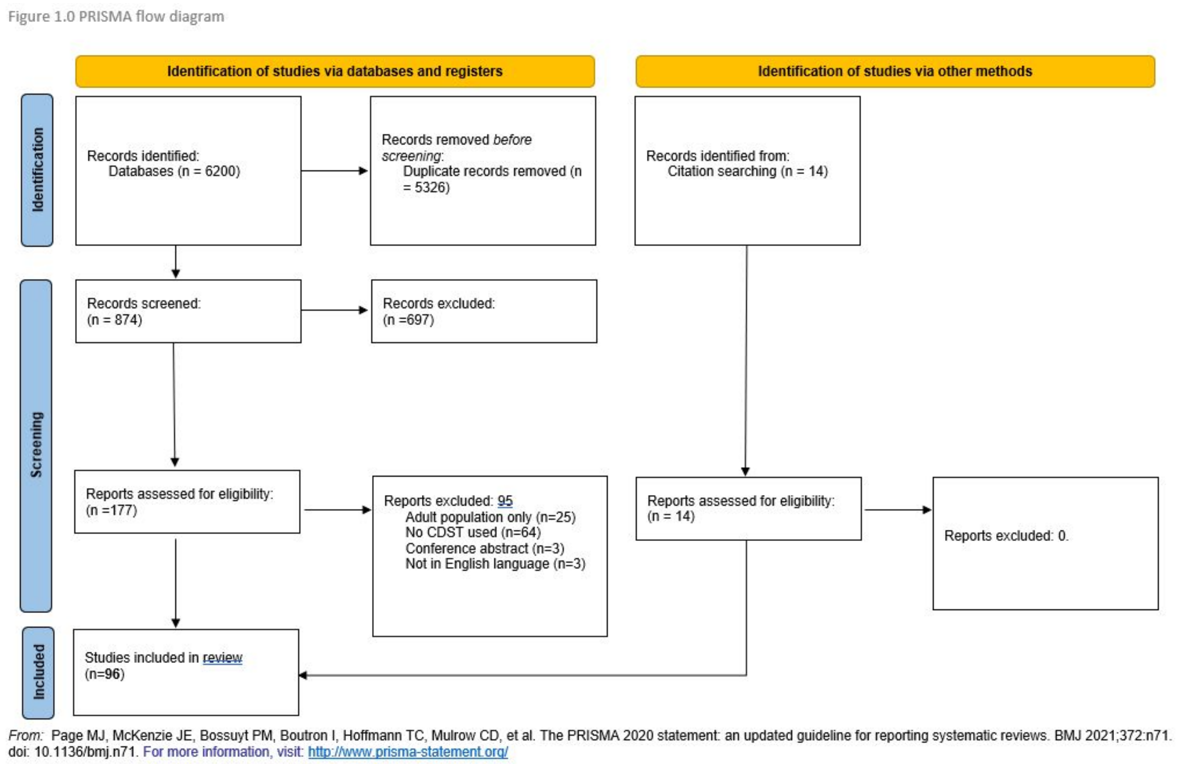

In total, 25 relevant in-hospital clinical decision support tools for the assessment and management of children with head injury were identified, displayed in Table 3.

Table 3. Hospital based clinical decision support tools for the assessment and management of children with head injury:

Clinical Decision Support Tools	Full title	Reference
1. PECARN	Paediatric Emergency Care Applied Research Network rule.	[6] Kuppermann, N., *et al.* (2009) Identification of children at very low risk of clinically important brain injuries after head trauma: A prospective cohort study. *The Lancet,* 374(9696), pp. 1160-1170.
2. CHALICE	Children's Head injury Algorithm for the prediction of Important Clinical Events Rule.	[13] Dunning, J., Daly, J., Lomas, J., Lecky, F., Batchelor, J. and Mackway-Jones (2006). Derivation of the children's head injury algorithm for the prediction of important clinical events decision rule for head injury in children. *Archives of Disease in Childhood,* 91(11), pp. 885-91.
3. CATCH	Canadian Assessment of Tomography for Childhood Head injury Rule.	[14] Osmond, M.H. (2010) CATCH: a clinical decision rule for the use of computed tomography in children with minor head injury, *Canadian Medical Association Journal,* 182(4).
4. NEXUS II	National Emergency X-Ray Utilization Study.	[15] Oman, J. A., Cooper, R. J., Holmes, J. F., Viccellio, P., Nyce, A., Ross, S. E., ... & Mower, W. R. (2006).

For peer review only - http://bmjopen.bmj.com/site/about/guidelines.xhtml

		Predictive performance of a decision rule to predict need for computed tomography among children with blunt head trauma. *Pediatrics,* 117(2), pp. 238-246.
5. HIDATq	Head Injury Discharge At Triage questionnaire.	[5] Aldridge, P., Castle, H., Phillips, C., Russel, E., Guerrero-Luduena, R. and Rout, R. (2020). Head home: a prospective cohort study of a nurse led paediatric head injury clinical decision tool at a district general hospital, Emergency Medical Journal, 37(11), pp. 680-685.
6. PALCHAK	UC Davis Rule for Paediatric Head Trauma.	[16] Palchak, M.J *et al.* (2003). A decision rule for identifying children at low risk for brain injuries after blunt head trauma, *Annals of Emergency Medicine*, 42(4), pp. 492-506
7. HAYDEL	New Orleans/Charity Head Trauma/Injury Rule.	[17] Haydel, M.J. and Shembekar, A. D. (2003) Prediction of intracranial injury in children aged five years and older with loss of consciousness after minor head injury due to nontrivial mechanisms, *Annals of Emergency Medicine,* 42(4).
8. ATABAKI	No title	[18] Atabaki, S. M., Stiell, I. G., Bazarian, J. J., Sadow, K. E., Vu, T. T., Camarca, M. A., ... & Chamberlain, J. M. (2008). A clinical decision rule for cranial computed tomography in minor pediatric head trauma. *Archives of pediatrics and adolescent medicine,* 162(5), pp. 439-445
9. GREENES	No title	[19] Greenes, D. S., & Schutzman, S. A. (1999). Clinical indicators of intracranial injury in head-injured infants. *Pediatrics,* 104(4), pp. 861-867.
10. KLEMETTI	No title	[20] Klemetti, S., Uhari, M., Pokka, T., & Rantala, H. (2009). Evaluation of decision rules for identifying serious consequences of traumatic head injuries in pediatric patients. Pediatric emergency care, 25(12), 811-815
11. QUAYLE	No title	[21] Quayle, K. S., Jaffe, D. M., Kuppermann, N., Kaufman, B. A., Lee, B. C., Park, T. S., & McAlister, W. H. (1997). Diagnostic testing for acute head injury in children: when are head computed tomography and skull radiographs indicated? *Pediatrics,* 99(5).
12. DIETRICH	No title	[22] Dietrich, A. M., Bowman, M. J., Ginn-Pease, M. E., Kosnik, E., & King, D. R. (1993). Pediatric head injuries: can clinical factors reliably predict an abnormality on computed tomography? *Annals of emergency medicine, 22*(10), pp. 1535-1540.
13. GUZEL	No title	[23] Güzel, A., Hiçdönmez, T., Temizöz, O., Aksu, B., Aylanç, H., & Karasalihoglu, S. (2009). Indications for brain computed tomography and hospital admission in pediatric patients with minor

For peer review only - http://bmjopen.bmj.com/site/about/guidelines.xhtml

		head injury: how much can we rely upon clinical findings?. *Pediatric neurosurgery,* 45(4), pp. 262-270
14. PredAHT	Predicting Abusive Head Trauma	[24] Cowley, Laura E *et al.* (2018) Acceptability of the Predicting Abusive Head Trauma (PredAHT) clinical prediction tool: A qualitative study with child protection professionals, *Child Abuse & Neglect* 81, pp. 192-205.
15. CHIDA	Children's Intracranial Injury Decision Aid.	[25] Neumayer, Katie *et al.* (2020) Validation of the "CHIIDA" and application for PICU triage in children with complicated mild traumatic brain injury. *Journal of pediatric surgery*, 55(7), pp. 1255-1259
16. CIDSS2	Stands for:<br>Coma scale<br>Intoxication<br>Deficit (neurological)<br>Seizure<br>Skull fracture 2	[26] Greenberg, J., Yan, Y., Carpenter, C., Lumba-Brown, A., Keller, M., Pineda, J., Brownson, R. and Limbrick, D. (2018) Development of the $CIDSS_2$ Score for Children with Mild Head Trauma without Intracranial Injury. *Journal of Neurotrauma,* 35(22), pp. 2699-2707.
17. PediBIRN	Paediatric Brain Injury Research Network	[27] Pfeiffer, H., Smith, A., Kemp, M., Cowley, E., Cheek, A., Dalziel, S., Borland, S., O'Brien, S., Bonisch, M., Neutze, J., Oakley, E., Crowe, L., Hearps, S., Lyttle, M., Bressan, S. and Babl, F. (2018) External validation of the PediBIRN clinical prediction rule for abusive head trauma. *Paediatrics,* 141(5).
18. Head CT Choice	Head Computed Tomography Choice	[28] Hess, Erik *et al.* (2014) Effectiveness of the head CT choice decision aid in parents of children with minor head trauma: study protocol for a multicentre randomized trial. *Trials,* 15(1), pp. 253-253
19. SNC Tool	The Scandinavian Neurotrauma Committee Guidance	[29] Unden, J. *et al.* (2018) External validation of the Scandinavian guidelines for management of minimal, mild and moderate head injuries in children, *BMC Medicine,* 16(176).
20. BIG-1	Brain Injury Guidelines	[30] Schwartz, Jamie *et al.* (2020)<br>Applying Pediatric Brain Injury Guidelines at a Level I Adult/Pediatric Safety-Net Trauma Center.<br>*The Journal of surgical research,* 255, pp. 106-110
21. Novel Simplified CDR	Novel Simplified Clinical Decision Rule	[31] Yogo, N., Toida, C., Muguruma, T., Gakumazawa, M., Shinohara, M. and Takeuchi, I. (2021) Simplified Clinical Decision Rule Using Clinically Important Events for Risk Prediction in Pediatric Head Injury: A Retrospective Cohort Study, *Journal of Clinical Medicine,* 10(22).

For peer review only - http://bmjopen.bmj.com/site/about/guidelines.xhtml

22. Head Trauma EBG Algorithm	Head Trauma EBG Algorithm	[32] Brittany M. Stopa*, et al.* (2019) Comparison of minor head trauma management in the emergency departments of a United States and Italian Children's hospital, *Italian Journal of Pediatrics,* 45(24).
23. Da Dalt	No title	[33] Da Dalt, L. *et al*. (2006) Predictors of intracranial injuries in children after blunt head trauma. *European Journal of Paediatrics,* 165(3), pp. 142–8.
24. Buchanich	No title	[34] Buchanich, J. M. (2007). A clinical decision-making rule for mild head injury in children less than three years old (Doctoral dissertation, University of Pittsburgh).
25. KIIDS-TBI- CDS	The Kids Intracranial Injury Decision Support tool for Traumatic Brain Injury	[35] Greenberg *et al*. (2021) Development and external validation of the KIIDS-TBI tool for managing children with mild traumatic brain injury and intracranial injuries. *Academic Emergency Medicine,* 28(12).

Sixty-seven decision-making criteria were extracted across all tools. Several criteria related to the same clinical feature (for example, vomiting), however there were many different cut points (e.g., vomiting once only, vomiting twice, or vomiting three times). These 67 criteria were grouped into 18 clinical categories: vomiting; headache; loss of consciousness (LOC); seizure; amnesia; dizziness/vertigo; clotting impairment/anticoagulated; non-accidental injury; age; severe mechanism of injury; drug/alcohol use; signs of a skull fracture; haematoma; neurological deficit; altered mental status; Glasgow come score (GCS); drowsiness and well with no clinical concern.

Table 4 shows which clinical categories are included within each hospital-based tool.

For peer review only

Table 4. Categories in each tool.

	Signs of Skull fracture	Scalp Haematoma	Vomiting	Neuro Deficit	Headache	LOC	AMS	GCS	Seizure	Severe mechanism of injury	Drowsy	Amnesia	Vertigo	Non accidental injury	Clotting impairment/ anticoagulated	Drug/ alcohol use	Patient alert and well	Age
PECARN (2-18)	YES	NO	YES	NO	YES	YES	YES	YES	NO	YES	YES	NO	NO	NO	NO	NO	NO	NO
PECARN (<2)	YES	YES	NO	NO	NO	YES	YES	YES	NO	YES	YES	NO	NO	NO	NO	NO	YES	YES
CHALICE	YES	YES	YES	YES	NO	YES	NO	YES	YES	YES	YES	YES	NO	YES	NO	NO	NO	NO
CATCH	YES	YES	NO	NO	YES	YES	YES	YES	NO	YES	NO	YES	NO	NO	NO	NO	NO	NO
NEXUS 2	YES	YES	YES	YES	NO	YES	YES	YES	NO	NO	YES	YES	NO	NO	YES	NO	NO	NO
HIDATq	YES	YES	YES	YES	NO	YES	YES	YES	YES	YES	YES	YES	NO	YES	YES	NO	YES	YES
PALCHACK	YES	YES	YES	NO	YES	NO	YES	NO	NO	NO	NO	NO	NO	NO	NO	NO	NO	NO
HAYDEL	NO	YES	YES	NO	YES	NO	NO	NO	YES	NO	NO	YES	NO	NO	NO	YES	NO	YES
ATABAKI	YES	YES	YES	YES	YES	YES	YES	YES	YES	YES	YES	YES	YES	NO	NO	YES	NO	YES
GREENES	YES	YES	NO	NO	NO	NO	NO	NO	NO	NO	NO	NO	NO	NO	NO	NO	NO	NO
KLEMETTI	YES	YES	NO	YES	NO	YES	YES	NO	NO	NO	NO	NO	YES	NO	NO	NO	NO	NO
QUAYLE	YES	NO	NO	YES	NO	YES	YES	NO	YES	NO	NO	NO	NO	NO	NO	NO	NO	NO
DIETRICH	NO	NO	YES	YES	YES	YES	NO	NO	YES	NO	NO	NO	NO	NO	NO	NO	NO	NO
GUZEL	YES	YES	NO	YES	YES	YES	NO	NO	YES	YES	NO	YES	NO	NO	NO	NO	NO	NO
PredAHT	NO	YES	NO	NO	NO	NO	NO	NO	YES	NO	NO	NO	NO	NO	NO	NO	NO	NO
CHIDA	YES	NO	NO	NO	NO	NO	NO	YES	NO	NO	NO	NO	NO	NO	NO	NO	NO	NO
CIDSS2	YES	NO	NO	YES	NO	NO	NO	YES	YES	NO	NO	NO	NO	NO	NO	YES	NO	NO
PediBIRN	YES	YES	NO	NO	NO	NO	NO	NO	NO	NO	NO	NO	NO	NO	NO	NO	NO	NO
HEAD CT CHOICE	NO	NO	YES	YES	YES	NO	YES	YES	NO	NO	YES	NO	NO	NO	NO	NO	NO	NO
SNC GUIDELINE	YES	YES	YES	YES	YES	YES	YES	YES	YES	NO	NO	NO	NO	NO	YES	NO	NO	NO
BIG-1	YES	NO	NO	YES	NO	YES	NO	NO	NO	NO	NO	NO	NO	NO	NO	YES	NO	NO
NOVEL CDR	NO	NO	NO	NO	YES	YES	YES	YES	YES	YES	NO	NO	NO	NO	NO	NO	NO	NO
HEAD TRAUMA EBG	YES	YES	YES	YES	NO	YES	YES	YES	NO	YES	NO	NO	NO	NO	NO	NO	NO	YES
DA DALT	YES	NO	YES	YES	YES	YES	YES	YES	YES	NO	YES	YES	NO	NO	NO	NO	NO	NO
BUCHANIC	YES	YES	YES	YES	YES	NO	YES	NO	NO	NO	NO	NO	NO	NO	NO	NO	NO	NO
KIIDS-TBI CDS	YES	YES	NO	NO	NO	NO	NO	YES	NO	NO	NO	NO	NO	NO	NO	NO	NO	NO

For peer review only - http://bmjopen.bmj.com/site/about/guidelines.xhtml

Of these clinical categories, the most frequent were skull fracture, loss of consciousness, and scalp haematoma. The most infrequently used included vertigo, suspicion of non-accidental injury and patient acting normal as per parent. Table 5 tabulates the frequency of each clinical criterion.

Table 5. Frequency of each clinical category across all the tools.

Categories	CDST	Frequency (n=25)
Signs of any description of skull fracture<br><br>(Depressed, basal skull, palpable)	PECARN 18, PECARN 2, NEXUS 2, HIDATq, PALCHACK, CHALICE, CATCH, GUZEL, BUCHANICH, DA DALT, KLEMETTI, QUAYLE, ATABAKI, SNC Guideline, BIG-1, Head trauma EBG algorithm, GREENES, CHIDA, CIDSS2, PediBIRN, KIIDS-TBI CDS	**21 (84%)**
LOC	PECARN 18, PECARN 2, CHALICE, CATCH, NEXUS 2, ATABAKI, KLEMETTI, QUAYLE, HIDATq, GUZEL, DIETRICH, DA DALT, BUCHANICH, SNC Guideline, BIG-1, Novel simplified CDR, Head trauma EBG algorithm	**17 (68%)**
Scalp haematoma/trauma	PECARN 2, CHALICE, PALCHAK, KLEMETTI, NEXUS 2, GUZEL, HAYDEL, GREENES, CATCH, HIDATq, BUCHANICH, ATABAKI, SNC Guideline, Head trauma EBG algorithm, PredAHT, PediBIRN, KIIDS-TBI CDS	**17 (68%)**
Neurological deficit	CHALICE, NEXUS 2, HIDATq, GUZEL, DIETRICH, DA DALT, BUCHANICH, KLEMETTI, ATABAKI, QUAYLE, Head CT Choice, SNC Guidelines, BIG-1, Head trauma EBG algorithm, CIDSS2	**15 (60%)**
Behaviour change/ altered mental status	PECARN 18, PECARN 2, CATCH, HIDATq, PALCHAK, ATABAKI, BUCHANICH, DA DALT, KLEMETI, QUAYLE, NEXUS 2, Head CT Choice, SNC Guidelines, Novel simplified CDR, Head trauma EBG algorithm	**15 (60%)**
GCS	PECARN 18, PECARN 2, CHALICE, NEXUS 2, CATCH, HIDATq, ATABAKI, CHIDA, CIDSS2, Head CT Choice, SNC	**15 (60%)**

For peer review only - http://bmjopen.bmj.com/site/about/guidelines.xhtml

For peer review only

	Guideline, Novel simplified CDR, Head trauma EBG algorithm, DA DALT, KIIDS-TBI CDT	
Vomiting	CHALICE, NEXUS 2, PECARN 18, PALCHACK, HIDATq, HAYDEL, DIETRICH, BUCHANICH, DA DALT, Head CT Choice, SNC Guidelines, Head trauma EBG algorithm	**12 (48%)**
Headache	PECARN 18, CATCH, PALCHAK, GUZEL, DIETRICH, HAYDEL, ATABAKI, BUCHANICH, DA DALT, Head CT Choice, SNC Guidelines, Novel simplified CDR	**12 (48%)**
Seizure	CHALICE, HIDATq, HAYDEL, ATABAKI, QUAYLE, DIETRICH, GUZEL, PredAHT, CIDSS2, SNC Guideline, Novel simplified CDR, DA DALT.	**12 (48%)**
Severe mechanism of injury	PECARN 18, PECARN 2, GUZEL CHALICE, CATCH, HIDATq, ATABAKI, Novel simplified CDR, Head trauma EBG algorithm	**9 (36%)**
Drowsiness	CHALICE, HIDATq, DA DALT, PECARN 18, PECARN 2, NEXUS 2, ATABAKI, Head CT Choice	**8 (32%)**
Amnesia	CHALICE, CATCH, NEXUS 2, HIDATq, HAYDEL, ATABAKI, GUZEL, DA DALT	**8 (32%)**
Drug or alcohol use	HIDATq, HAYDEL, ATABAKI, CIDSS2, BIG-1	**5 (20%)**
Age	PECARN, HIDATq, ATABAKI, HAYDEL	**4 (16%)**
Clotting impairment/anticoagulated	NEXUS 2, HIDATq, SNC Guideline	**3 (12%)**
Dizziness/vertigo	ATABAKI, KLEMETI	**2 (8%)**
Non accidental injury	CHALICE, HIDATq,	**2 (8%)**
Patient alert and well with no clinical concern/acting normal as per parent	PECARN 2, HIDATq	**2 (8%)**

The mix of clinical criteria used, and their outcomes were similar but not identical for any of the tools. In addition, the derivation studies used different populations and sample sizes and therefore the performance accuracy of the tools relating to the potential to correctly rule out clinically significant brain injury in head-injured children were variable *(*table 6).

For peer review only - http://bmjopen.bmj.com/site/about/guidelines.xhtml

For peer review only

Table 6. Performance accuracy of each tool.

Name of tool	Internal validity	External validity	Implementation/ evaluation	Performance accuracy
**PECARN (under 2)**	Positive evidence	Positive evidence	Mixed evidence supporting potential effect.<br><br>Mixed evidence supporting positive conclusion for implementation.	Sensitivity: 100%<br><br>Specificity: between 53% and 60%
**PECARN (2-18)**	Positive evidence	Positive evidence	Mixed evidence supporting potential effect.<br><br>Mixed evidence supporting positive conclusion for implementation.	Sensitivity: 96.8%<br><br>Specificity: between 53% and 60%
**CHALICE**	Positive evidence	Positive evidence	Mixed evidence supporting positive conclusion for implementation.	Sensitivity: 98%<br><br>Specificity: 86%
**CATCH**	Positive evidence	Positive evidence	Not evaluated for usability, potential effect, or post implementation impact.	Sensitivity: 98%<br><br>Specificity: 50%
**NEXUS II**	Positive evidence	Mixed evidence supporting positive conclusion	Not evaluated for usability, potential effect, or post implementation impact.	Sensitivity: 99%<br><br>Specificity: 15%

For peer review only

For peer review only - http://bmjopen.bmj.com/site/about/guidelines.xhtml

**HIDATq**	Positive evidence	Not reported	Not evaluated in a published study after development	Negative predictive value for CT was 100% for intracranial injury. The positive predictive value of the tool was low.
**PALCHACK**	Positive evidence	Positive evidence	Not evaluated in a published study after development	Sensitivity: 100%<br><br>Specificity: 46%
**HAYDEL**	Positive evidence	Not reported	Not evaluated in a published study after development	Sensitivity: 100%<br><br>Specificity: 24%
**ATABAKI**	Positive evidence	Not reported	Not evaluated in a published study after development	Sensitivity: 100%<br><br>Specificity: 46%
**BUCHANICH**	Positive evidence	Not reported	Not evaluated in a published study after development	Sensitivity: 100%<br><br>Specificity: 40%
**DA DALT**	Developed but not tested for internal validity	Not reported	Not evaluated in a published study after development	Sensitivity: 100%<br><br>Specificity: 87%
**GREENES**	Developed but not tested for internal validity	Not reported	Not evaluated in a published study after development	Sensitivity: 53%<br><br>Specificity: 72%
**KLEMETTI**	Developed but not tested for internal validity	Not reported	Not evaluated in a published study after development	Sensitivity: 94%<br><br>Specificity: 29%

For peer review only - http://bmjopen.bmj.com/site/about/guidelines.xhtml

**QUAYLE**	Developed but not tested for internal validity	Not reported	Not evaluated in a published study after development	Sensitivity: 44%<br><br>Specificity: 85%
**DIETRICH**	Developed but not tested for internal validity	Not reported	Not evaluated in a published study after development	Sensitivity: 100%<br><br>Specificity: 17%
**GUZEL**	Developed but not tested for internal validity	Not reported	Not evaluated in a published study after development	Sensitivity: 69%<br><br>Specificity: 43%
**PredAHT 2**	Positive evidence	Positive evidence	Not evaluated for usability, potential effect, or post implementation impact.	Sensitivity: 72%<br><br>Specificity: 86%
**CHIDA**	Negative evidence	Not reported	Not evaluated in a published study after development	Sensitivity: 94%<br><br>Specificity: 69%
**CIDSS2**	Positive evidence	Not reported	Not evaluated in a published study after development	Sensitivity: 51%<br><br>Specificity: 96%
**PediBIRN**	Positive evidence	Positive evidence	Not evaluated in a published study after development	Sensitivity: 96%<br><br>Specificity: 29%

For peer review only - http://bmjopen.bmj.com/site/about/guidelines.xhtml

For peer review only

**Head CT Choice**	Positive evidence	Not reported	Not evaluated in a published study after development	Not reported
**SNC guideline**	Positive evidence	Positive evidence	Not evaluated in a published study after development	Sensitivity: 95%<br><br>Specificity: 59%
**BIG-1**	Positive evidence	Not reported	Not evaluated in a published study after development	Not reported
**Novel simplified CDR**	Positive evidence	Not reported	Not evaluated in a published study after development	Sensitivity: 80%<br><br>Specificity: 51%
**Head trauma EBG algorithm**	Positive evidence	Not reported	Not evaluated in a published study after development	Not reported
**KIIDS-TBI CDS**	Positive evidence	Positive evidence	Not evaluated in a published study after development	Sensitivity: 100%<br><br>Specificity: 26%

For peer review only - http://bmjopen.bmj.com/site/about/guidelines.xhtml

For peer review only

Most tools displayed high sensitivities (>90%), while specificities ranged from 15 to 87%. PECARN was the most widely externally validated, with positive conclusions drawn from research examining post-implementation of the tool [36]. However, PECARN results in a considerable number of normal scans being performed to identify one intracranial injury [37]. The HIDATq tool was designed for nurses to use in triage to safely discharge children with head injury before admission, which more closely resembles the aim of a pre-hospital tool designed to inform the need for hospital conveyance. Table 7 displays a narrative synthesis of each clinical criteria within the tools.

Table 7*. *Narrative synthesis of clinical criteria:

	**Narrative description of findings**
**Signs of skull fracture**	This was the most frequently used clinical decision-making criterion (21/25) (84%). Variability of terms included: open or depressed skull fracture, basal skull fracture, tense fontanelle, palpable and unclear skull fracture. The only tools that do not include signs of skull fracture are HAYDEL, DIETRICH, PredAHT and Novel Simplified CDR. All three of PECARN, CHALICE and CATCH (the three most widely validated tools) included signs of skull fracture. PredAHT does not include signs of skull fracture despite the aim of this tool being to identify abusive head trauma. In contrast, PediBIRN which aims to identify children with abusive head trauma does include signs of skull fracture as a clinical predictor [38]. The KIIDS-TBI CDS Tool concluded that a depressed skull fracture was a major risk factor for significant brain injury [35]. Babl, *et al.* (2014) [39] and Da Dalt *et al.* (2006) [33] found that a basal, open or depressed skull fracture was most likely to trigger a CT response out of any other clinical criteria. Similarly, Quayle *et al.* (1997) [21] concluded that the relative risk for intracranial injury is increased almost fourfold in the presence of a skull fracture, although the absence of skull fracture does not rule out intracranial injury. The $CIDSS_2$ score [26], a tool to help physicians to identify the minority of children with minor traumatic brain injury at increased risk for extended inpatient stay, uses a scoring system; all clinical criteria score 1, apart from signs of skull fracture which scores 2, suggesting it is deemed the most significant clinical predictor of significant brain injury. Many of the tools, such as NEXUS 2, describe signs and symptoms of a skull fracture, rather than explicitly stating 'signs of a skull fracture'. For example: periorbital or periauricular ecchymoses, haemotympanum, and clear fluid from ears or nose [40].
**Scalp haematoma**	Scalp haematoma is used in 17 of the tools identified. However, many specify 'non-frontal' haematomas only, such as the NEXUS 2 tool [40]. Others (GREENES) support inclusion of non-frontal haematomas only; the authors found that non-frontal scalp haematomas had the strongest association with presence of intracranial haemorrhage [19]. The authors of PALCHAK concluded a scalp haematoma is more significant in children under 2 years [16]. The HIDATq tool does not stipulate location of haematoma, however the authors do specify a size (over 5cm) [5]. Similarly, CHALICE only specifies size, which is also over 5cm, but this is for under 1-year olds only [13]. PECARN clearly specifies 'temporal, occipital or parietal haematoma', and this is for the under 2-year-old tool [6]. The CATCH tool includes 'large boggy scalp haematoma', however it is unclear what constitutes 'large' as it does not specify what size or the location. Most excluded haematomas to the face.
**Loss of consciousness**	Loss of consciousness (LOC) has different cut points across the identified clinical decision tools, including 'any loss of consciousness', 'loss of consciousness for over 5 seconds' and 'loss of consciousness for over 5 minutes'. It is the second most frequently used clinical predictor, appearing in 17 of the 25 clinical decision tools. The authors of PALCHAK [16] found that the risk of a traumatic brain injury was higher in those with a history of loss of consciousness. However, in those children with an isolated loss of consciousness there were no traumatic brain injuries. This is recognised in Kupperman *et als.* (2009) derivation study for PECARN [6]; the risk of clinically important traumatic brain injury was substantially lower than 1% in children with isolated loss of consciousness. PECARN includes loss of consciousness for over 5

For peer review only - http://bmjopen.bmj.com/site/about/guidelines.xhtml

	seconds in the under 2 years of age cohort and loss of consciousness of an unspecified duration for the over 2 cohort. Differing to PECARN, CHALICE uses a duration of over 5 minutes of loss of consciousness in any child under 16 years of age, and HIDATq does not specify a duration of loss of consciousness.
**Vomiting**	This clinical criterion is a predictor in 12/25 tools, with four varying descriptors including 'any post injury vomiting', 'over 1 vomit', 'over 2 vomits' or 'over 3 vomits'. PECARN includes 'any vomiting' for the over 2-year-old cohort, as does the HIDATq tool, whereas CHALICE takes a different, less risk averse approach including '3 or more' [13]. Both CHALICE and PECARN have a high sensitivity and acceptable specificity for recognising clinically important traumatic brain injury, suggesting that both tools are appropriate for assessing mild head injury in children in the ED, despite the varying descriptions of vomiting. NEXUS 2 includes 'over 1 vomit' and CATCH stipulates 'over 2 vomits', but as eligibility criterion
**Neurological deficit**	For the purposes of this narrative description, neurological deficit will also include focal neurology, Glasgow Coma Scale score (GCS), altered mental status, behavioural change, drowsiness, and vertigo.<br>The research evidence that underpinned the DA DALT tool concluded that neurological deficit is one of the predictors that significantly increases the likelihood of intracranial injury [33]. Similarly, the CIDSS2 score concluded that neurological deficit was amongst the most important predictors for identifying whether paediatric patients should be admitted to hospital with a head injury [26]. There are varying descriptions to define a neurological deficit across the 25 tools, and there is some cross over with altered mental status/behavioural change, drowsiness, and GCS. Some terms include vision changes, gait changes, repetitive questioning/speech, delayed response to external stimuli, sensory deficit, disorientation or just stating 'neurological deficit'. Authors of the SNC guidelines suggest that the clinical criteria 'altered mental status' is too complicated to use effectively, with potential to lead to unacceptable increases in CT rates [29]. Both PECARN (under 2) and HIDATq include 'patient well with no clinical concern/acting normal as per parent', which suggests no neurological deficit.<br>Many tools opt to use 'abnormal mental status' with varying descriptors, such as confusion, somnolence, repetitive or slow to respond to verbal communication. A GCS under 15 is associated with the presence of intracranial acute pathologies in young children with minor blunt head trauma [41]. GCS is a clinical criterion in 15 of the 25 identified tools, including PECARN, CHALICE and CATCH.<br>KLEMETTI is one of the only tools to include 'vertigo' as a clinical criterion; the authors found this variable was amongst one of the most significant to predict severely complicated head trauma in their multivariate logistic regression analysis [20]. ATABAKI is the only other tool to include vertigo as a clinical criterion, however both these tools have low specificity and were not evaluated in a published validation study after development.
**Headache**	Headache is a clinical predictor in only half of the 25 in-hospital tools. This may be because it is challenging to assess for a headache in younger children; they may express pain differently with signs such as irritability and persistent crying [42]. The authors of the BUCHANIC tool use headache as a clinical predictor, despite an inclusion criterion of aged under 3 years only, however, this tool is not validated. PECARN includes headache for the aged 2- 18 years cohort, but not the aged under 2 years. For children who can verbalise or show that they have a headache, it is important to consider severity and timing of the headache. Some tools specify 'severe headache', 'prolonged headache' or 'worsening headache'. Worsening headache is associated with intracranial injury, being deemed a 'high risk' criterion in CATCH [14]. The HIDATq tool does not include headache as a clinical predictor, despite presenting the tool as 'ultra-safe', although it does include 'irritability' which could indicate pain in a younger child.
**Seizure**	Just under half of the 25 in-hospital tools included seizure as a clinical predictor despite a post traumatic seizure being a high determinant for an intracranial injury [43]. The CIDSS2 score

For peer review only - http://bmjopen.bmj.com/site/about/guidelines.xhtml

	found seizure to be one of the most significant criteria to identify whether patients should be admitted with head injury. PECARN is the most widely validated and implemented tool, however it does not include seizure as a specific clinical predictor, compared with CHALICE and HIDATq, which do.
**Severe mechanism of injury**	Nine of the 25 identified clinical decision support tools include 'severe or dangerous mechanism of injury' as a clinical predictor. The description of what constitutes a 'severe' mechanism of injury varies across the tools, from different levels of fall from height, road traffic collisions, to specific considerations such as a bicyclist not wearing a helmet who is struck by a car, however; fall from height is the most common mechanism of injury in children. The SNC guideline [29] does not include high-energy trauma mechanism as a strict risk factor, as these patients are relatively uncommon in Scandinavia and are managed according to separate clinical trauma protocols. The authors and creators of the SNC guidelines judged 'mechanism of injury' as a complicated clinical predictor to use, due to having a specific definition and often including assessment of fall height, vehicle speed and number of stairs [29]. Similarly, the Novel Simplified Tool [31] suggests that mechanism of injury is not considered reliable, and that it may be an overestimation to emphasise the injury mechanism as a predictor. The aim of this clinical decision tool is to provide a simplified version of PECARN, CATCH and CHALICE (all of which include varying descriptors for mechanism of injury), so that it can be easily and quickly used for paediatric patients in busy emergency settings. Therefore, one distinguishing feature is that that this tool includes only one predictor for mechanism of injury, however; the Novel CDR misclassified eight patients as low risk.
**Amnesia**	The authors of PALCHAK conclude that elimination of amnesia as a clinical predictor may decrease unnecessary CT scanning [16]. However, amnesia is a clinical predictor in eight of the 25 hospital-based tools identified within this review. It is not possible to assess amnesia in non-verbal children and it is difficult to assess in young children [33] which is perhaps why PECARN does not include amnesia as a clinical predictor, however all of CHALICE, CATCH, NEXUS 2 and HIDATq do. One study adapted the PECARN tool by introducing amnesia alongside other clinical predictors [44]. The authors concluded that the adapted PECARN rule was successfully implemented in an Italian Paediatric ED; its use determined a low CT scan rate that was unchanged compared to previous clinical practice and showed an optimal safety and high efficacy profile.
**Non-accidental injury**	Non-accidental injury is only included as a clinical predictor in two of the in-hospital clinical decision support tools (HIDATq and CHALICE), although some tools include non-accidental injury as an exclusion criterion, such as the Head Injury EBG algorithm and CATCH. Despite this, non-accidental injury is one of the biggest predictors of brain injury, particularly in children under the age of 2 years [45]. PECARN is not especially sensitive for identifying physically abused children [46]. There is no gold standard diagnostic test to identify abusive head trauma though PredAHT and PediBIRN are specifically designed tools that can help clinicians to recognise cases of abusive head trauma [46].
**Clotting impairment**	Only three of the 25 in-hospital tools include clotting/impairment as a clinical predictor (HIDATq, the SNC guideline and NEXUS 2), since it is uncommon for a child to be on these types of drugs or have bleeding diatheses. The HIDATq tool is derived using the NICE head injury guidance and is designed to be 'ultra-safe' since the aim of the tool is to discharge children with head injury at triage, and therefore includes 'anticoagulant use/clotting impairment. None of the three most widely validated tools (PECARN, CATCH and CHALICE) include 'anticoagulant use/clotting disorder' as a clinical predictor, because it was too rare to be of use.
**Drug and alcohol use**	It is well known that alcohol increases the risk of suffering a head injury due to its effects on balance and coordination [47]. Children with suspected drug or alcohol intoxication may be more difficult to assess; clinicians should assume conscious level and amnesia relates to injury and have a lower threshold for referral and neuroimaging, since blunt head injuries in

For peer review only - http://bmjopen.bmj.com/site/about/guidelines.xhtml

	intoxicated patients is associated with a higher mortality [48]. Alcohol can also lead to headaches, nausea, and irritability, making examination challenging. Five of the identified clinical decision support tools included ‘drug/alcohol use’ (HIDATq, HAYDEL, ATABAKI, CIDSS2, BIG-1). None of the most validated tools, PECARN, CHALICE, or CATCH include this as a clinical predictor.
**Age**	All the identified clinical decision support tools in this review specify an age in which the tool can be used, e.g., under 18 years, and this varies from aged under 2-years to under 21-year-olds. However, only two tools exclude younger age groups as a clinical predictor (HIDATq excludes under 1s and ATABAKI excludes under 2’s). HAYDEL is only for five–17-year-olds and PECARN has a separate tool for under 2’s, with different questions, more specific to that age group [6]. BUCHANIC, PredAHT and PediBIRN are designed specifically for under 3’s, and GREENES is for under 2’s only, however despite these tools aiming at the same age group, their clinical predictors still vary considerably. The authors of GREENES found that infants are at greater risk of intracranial hypertension due to their open fontanels and that under 1’s with intracranial injuries are frequently asymptomatic, making management inherently difficult for clinicians [19]. It may be for this reason (as well as the increased likelihood of non-accidental injury) that children under 3 months were significantly more likely to receive a CT scan using the PECARN tool.

DISCUSSION:

This systematic mapping review identified and examined clinical criteria within existing hospital-based clinical decision support tools for children with minor head injury to determine their utility in paramedic practice, to inform the need for hospital conveyance. There are additional factors which require consideration in operationalising a tool for pre-hospital use, including the purpose of existing clinical decision rules, and additional relevant clinical factors.

Challenges of selecting a tool for use in pre-hospital care:

None of the existing hospital-based clinical decision support tools can be translated directly into paramedic practice. No single hospital based clinical decision support tool included all 18 clinical criteria, and there are potentially additional criteria that are not included in any of the tools that may be useful specifically in pre-hospital care; however, this needs to be explored in subsequent work. Most hospital based clinical decision support tools are designed to identify children who need a CT scan; although, this is not actually the clinical end point of interest. The reason for doing a CT scan is to seek intracranial injury that requires neurosurgery. Arguably, if a child receives no active treatment, then a CT scan is unnecessary, even if it does show intracranial injury [49] especially as paediatric CT scanning results in a significantly increased lifetime risk of radiation induced fatal malignancy [50]. The point of an out-of-hospital clinical decision support tool designed for use by paramedics is to identify children who would benefit from hospital conveyance for CT scanning to seek intracranial injury requiring neurosurgical intervention. This is accepting that there are complicating issues specific to pre-hospital care such as local ambulance policy, time of day, time since injury, complex wound closure, safeguarding, parental concern, and parental capability, that need to be considered and may warrant conveyance [51].

All the existing hospital-based tools address diverse questions, age groups, injury severities, and outcomes and have been derived, evaluated, and validated in a different way. In comparison to CHALICE, CATCH and other hospital-based tools, PECARN identifies children with a very low risk of brain injury who *do not* need a scan, which is an important concept, because it considers who a clinician *does not* need to scan, rather than who they *do* [6]. PECARN was derived using two large

For peer review only - http://bmjopen.bmj.com/site/about/guidelines.xhtml

multicentre cohorts with over 40,000 children and is extremely sensitive, particularly for infants. A tool designed for use by paramedics in the pre-hospital setting could follow this approach and identify children who *do not* need conveyance, due to sustaining a trivial or minor head injury that is very low risk of clinically significant brain injury, similarly to Kupperman's PECARN [6] rather than identifying those who *do* need conveyance, though this needs further consideration. PECARN is also the only tool that considers the age of the child from the beginning and thereafter asks different questions relevant to the child's age [6]. Paramedics have reported difficulty using the NICE head injury guidance [4] for this reason, as they could not ask younger children about amnesia, headache, and alcohol use [51].

What clinical criteria should be included:

All 18 clinical criteria identified in this systematic mapping review are important to consider for a pre-hospital tool designed to support paramedics in their assessment and management of children with head injury, however some may be difficult to operationalise in the pre-hospital setting. In addition, the hospital-based tools have varying cut off points for different clinical criteria and which descriptor a pre-hospital tool should use requires further consideration.

Certainty of use:

It is clear from the evidence that some clinical predictors are significant for identifying a potentially clinically important traumatic brain injury following a head injury and should be included in a pre-hospital tool, such as a post-traumatic seizure [43]. Signs of a skull fracture was most likely to trigger a CT response over any other clinical criteria, since the risk of intracranial injury is significantly increased in the presence of a skull fracture. This is reflected in the NICE guidance for the management of head injury, which suggests a CT scan should be performed within an hour if there are signs of a skull fracture [4]. The tools indicated that specifically a 'large', or 'non-frontal' (particularly parietal) [19] or 'boggy' haematoma, especially in infants, is more indicative of significant intracranial injury, which is apparent in other research [52]. Under 6-month-olds have a higher likelihood of significant intracranial haemorrhage with haematomas [52]. Greenes and Schutzman (2001) [19] suggest that among asymptomatic head-injured infants, the risk of skull fracture and associated intracranial haemorrhage is correlated with both scalp haematoma size and location. In most ambulance trusts across the United Kingdom, local policy stipulates that any under 2-year-old should be conveyed to the ED, no matter how mild the presenting injury [51]. Abusive head trauma occurs more frequently in under 1's [53]; paramedics are in a unique position to identify suspected non accidental injury since they attend the patient's home environment often in a time of crisis and have a professional duty to protect vulnerable persons [54]. This evidence supports the recommendation of including 'infants' as a clinical predictor for a pre-hospital tool designed to aid decision making regarding conveyance, or to exclude under 1's entirely. Non accidental injury is only a clinical predictor in two in-hospital clinical decision tools, however; perhaps this is because it is seen as a "no-brainer" and children were often excluded from the study cohort, as they would always need a scan. A child with suspected non accidental injury should always be conveyed to the ED by paramedics. Abuse tools such as those derived by Pfeiffer [27] and Cowley [24] include certain clinical predictors that can help paramedics in recognising non accidental injury.

Clinical predictors that may need intervention:

The pre-hospital environment is very different to in-hospital and there are some clinical predictors that may warrant conveyance as they may need interventions that cannot be done pre-hospitably, such as observation and complex wound closure. In addition, there are factors that are outside of a

For peer review only - http://bmjopen.bmj.com/site/about/guidelines.xhtml

paramedic's scope of practice that presents too much risk to discharge at scene, and therefore warrants conveyance.

The number of vomits was variable across the tools; research suggests that children who vomit after a head injury is less significant than in adults and they do not necessarily have a serious brain injury [55]. Other literature supports this, suggesting that clinically important traumatic brain injuries are uncommon in children presenting with head injury with vomiting and a management approach of observation without immediate CT may be appropriate [56]. However, a period of observation is not always practical or possible for paramedics in the pre-hospital setting. This was reported by paramedics in a survey exploring reasons why paramedics convey children with minor head injury to the ED [51]. However, most children with head injury (90%) who are admitted for observation are discharged without any further treatment being given [57]. Notably, *persistent* vomiting post head injury is indicative of a subdural haematoma or skull fracture; Harper *et al.* (2020) [58] supports this, concluding that discreet vomiting over four times was a significant risk factor for intracranial injury. Additionally, vomiting combined with other clinical criteria such as headache and altered mental status, can indicate a more serious head injury [56]. This theme is clear with other clinical predictors such as loss of consciousness; children with minor blunt head trauma presenting to the ED with *isolated* loss of consciousness are at very low risk for clinically significant brain injury and do not routinely require CT scanning [59], however if the loss of consciousness was associated with other clinical predictors, the risk is increased. Any clinical predictor present in isolation is less likely to indicate clinically significant traumatic brain injury than the presence of multiple risk criteria [56]. This is similar for mechanism of injury; evidence implies that mechanism of injury alone is less predictive of an abnormal brain CT, compared with other clinical predictors [60]. Despite this, most paramedics would convey a head-injured child based solely on mechanism, despite them appearing well with no clinical concern [51].

Fowler *et al.* (2018) [61] found that attending to paediatric patients evokes anxiety and discomfort amongst paramedics, which often led to reluctance to initiate treatment and poorer care. This includes unnecessary transport to the ED. Approximately half of paramedic respondents in a survey reported feeling 'not at all confident' with completing a neurological assessment on a child following a head injury [51], despite a focal neurological deficit being a strong indicator of intracranial injury [33]. GCS is inherently difficult to assess in a younger child, however a reduced GCS is a high-risk factor for clinically significant intracranial injury [41].

Clinical predictors that infrequently result in intracranial injury:

There is limited evidence to suggest vertigo as a standalone clinical predictor should be included in a decision tool for conveyance of children with head injury, and this needs further investigation [20]. Additionally, evidence is still conflicting regarding the risk of intracranial haemorrhage in children with a clotting impairment/anticoagulation, [62], despite this being a research priority since 2011 [63]. Bressan *et al.* (2020) [63] found that although children with a bleeding disorder who have sustained a head injury more often received a CT scan, their risk of intracranial haemorrhage was low. Their findings are echoed in other literature [64]; CTs are obtained twice as often in children with bleeding disorders, although intracranial haemorrhage occurred in only 1.1% of these patients, and they had symptoms that would have prompted the need for a CT anyway. New guidance from NICE [4] advocates a CT head scan within eight hours for children on anticoagulant treatment, or within an hour if they have a bleeding/clotting disorder, independently of other indications. However, only one out of 31 children on anticoagulants in Bressen *et als.* (2020) [62] and Lee *et als*. (2011) [64] study combined, was diagnosed with an intracranial haemorrhage, and that patient very obviously needed

For peer review only - http://bmjopen.bmj.com/site/about/guidelines.xhtml

a CT from the outset. These clinical criteria need further consideration before being included in a pre-hospital tool for paramedic use.

LIMITATIONS:

The role of mapping reviews is to provide a descriptive account of the published literature; therefore, this should be considered when assessing the findings of the overall evidence synthesis. A direct comparison of the clinical decision tools is not possible because they address different questions, age groups, injury severities, and outcomes. None of the evidence is from the pre-hospital environment. Although rigorous methods of identifying all in-hospital tools have been followed, it is possible that some studies have been overlooked. This is particularly the case for international studies since this review included English language articles only.

CONCLUSIONS:

Clinical predictors that markedly increase the likelihood of neurosurgical intervention and should be considered for inclusion in a clinical decision support tool for use by paramedics to assess the need for conveyance are: signs of a skull fracture; a large, boggy or non-frontal haematoma (particularly in an infant); persistent discreet episodes of vomiting; a focal neurological deficit; GCS less than 15; a prolonged or worsening headache; a prolonged loss of consciousness; a post traumatic seizure; amnesia in older children; suspicion of non-accidental injury; drug or alcohol use and being under 1 year old. Clinical criteria that require further investigation include mechanism of injury; clotting impairment/anticoagulated; vertigo; length of time of loss of consciousness; number of vomits, and description of what constitutes a neurological deficit in younger children. Any clinical predictor present in isolation is unlikely to indicate clinically significant traumatic brain injury.

There are likely to be additional clinical criteria that are relevant to paramedic assessment and practice, which are not included in any of these tools; such criteria would need to be identified and explored in further work. None of the existing hospital-based clinical decision support tools can be directly implemented into paramedic practice; however, elements from each of the tools can be adapted to create a new tool specific for paramedics in pre-hospital care. Future qualitative research is needed to investigate paramedics' thoughts on a clinical decision tool designed to support them in their assessment and management of head-injured children.

REFERENCES:

1. NHS (2019) The NHS Long term Plan. Available at: https://www.longtermplan.nhs.uk/ [Accessed 30th January 2023].
2. Arora, R., White, E.N., Niedbala, D., Ravichandran, Y., Sethuraman, U. and Radovic, N. (2020) Reducing Computed Tomography Scan Utilization for Paediatric Minor Head Injury in the Emergency Department: A Quality Improvement Initiative. *Academic Emergency Medicine,* 28(6), pp. 655-665.
3. Healthcare Quality Improvement Partnership (2015) Traumatic head injury in children and young people: a national overview. Available at: https://www.hqip.org.uk/wp-content/uploads/2018/02/traumatic-head-injury-in-children-and-young-people-a-national-overview.pdf [Accessed 30th January 2023].
4. National Institute for Health and Care Excellence (2023) *Head Injury Management.* Available at: https://cks.nice.org.uk/topics/head-injury/management/ [Accessed 23 May 2023].

For peer review only - http://bmjopen.bmj.com/site/about/guidelines.xhtml

5. Aldridge, P., Castle, H., Phillips, C., Russel, E., Guerrero-Luduena, R. and Rout, R. (2020). Head home: a prospective cohort study of a nurse led paediatric head injury clinical decision tool at a district general hospital, Emergency Medical Journal, 37(11), pp. 680-685.
6. Kuppermann, N., *et al.* (2009) Identification of children at very low risk of clinically-important brain injuries after head trauma: A prospective cohort study. *The Lancet,* 374(9696), pp. 1160-1170.
7. Campbell, F., Tricco, A.C., Munn, Z., Pollock, D., Saran, A., Sutton, A., White, H. and Khalil, H. (2023) Mapping reviews, scoping reviews, and evidence and gap maps: the same but different – the big picture review family, *Systematic reviews*, 12(45).
8. Grant, M.J. and Booth, A. (2009) A typology of reviews: an analysis of 14 review types and associated methodologies. *Health Info Library Journal,* 26(91), pp.108-9.
9. Clapton, J., Rutter, D. and Sharif, N. (2009) *Social Care Institute for Excellence (SCIE) systematic mapping guidance.* Available at: https://www.scie.org.uk/publications/researchresources/rr03.pdf [Accessed 30 January 2023].
10. Page, M.J., Mckenzie, J.E., Bossuyt, P.M., Hoffman, T.C. and Mulrow, C.D. (2020) The PRISMA 2020 statement: an updated guideline for reporting systematic reviews. BMJ, 172(71).
11. Covidence systematic review software, Veritas Health Innovation, Melbourne, Australia. Available at www.covidence.org [Accessed 18th April 2023].
12. Cooper, D. (2016). What is a "mapping study?" *Journal of the Medical Library Association,* 104(1), pp. 76-78.
13. Dunning, J., Daly, J., Lomas, J., Lecky, F., Batchelor, J. and Mackway-Jones (2006). Derivation of the children's head injury algorithm for the prediction of important clinical events decision rule for head injury in children. *Archives of Disease in Childhood,* 91(11), pp. 885-91.
14. Osmond, M.H (2010) CATCH: a clinical decision rule for the use of computed tomography in children with minor head injury, *Canadian Medical Association Journal,* 182(4).
15. Oman, J. A., Cooper, R. J., Holmes, J. F., Viccellio, P., Nyce, A., Ross, S. E., ... & Mower, W. R. (2006). Predictive performance of a decision rule to predict need for computed tomography among children with blunt head trauma. *Pediatrics,* 117(2), pp. 238-246.
16. Palchak, M.J *et al.* (2003). A decision rule for identifying children at low risk for brain injuries after blunt head trauma, *Annals of Emergency Medicine*, 42(4), pp. 492-506
17. Haydel, M.J. and Shembekar, A. D. (2003) Prediction of intracranial injury in children aged five years and older with loss of consciousness after minor head injury due to nontrivial mechanisms, *Annals of Emergency Medicine,* 42(4).
18. Atabaki, S. M., Stiell, I. G., Bazarian, J. J., Sadow, K. E., Vu, T. T., Camarca, M. A., ... & Chamberlain, J. M. (2008). A clinical decision rule for cranial computed tomography in minor pediatric head trauma. *Archives of pediatrics and adolescent medicine,* 162(5), pp. 439-445
19. Greenes, D. and Schutzman, S. (2001) Clinical significance of scalp abnormalities in asymptomatic head-injured infants. *Paediatric Emergency Care,* 17(2), pp. 88-92.
20. Klemetti, S., Uhari, M., Pokka, T., & Rantala, H. (2009). Evaluation of decision rules for identifying serious consequences of traumatic head injuries in pediatric patients. Pediatric emergency care, 25(12), 811-815
21. Quayle, K. S., Jaffe, D. M., Kuppermann, N., Kaufman, B. A., Lee, B. C., Park, T. S., & McAlister, W. H. (1997). Diagnostic testing for acute head injury in children: when are head computed tomography and skull radiographs indicated?. *Pediatrics,* 99(5).

For peer review only - http://bmjopen.bmj.com/site/about/guidelines.xhtml

22. Dietrich, A. M., Bowman, M. J., Ginn-Pease, M. E., Kosnik, E., & King, D. R. (1993). Pediatric head injuries: can clinical factors reliably predict an abnormality on computed tomography?. *Annals of emergency medicine, 22*(10), pp. 1535-1540.
23. Güzel, A., Hiçdönmez, T., Temizöz, O., Aksu, B., Aylanç, H., & Karasalihoglu, S. (2009). Indications for brain computed tomography and hospital admission in pediatric patients with minor head injury: how much can we rely upon clinical findings?. *Pediatric neurosurgery,* 45(4), pp. 262-270.
24. Cowley, Laura E *et al.* (2018) Acceptability of the Predicting Abusive Head Trauma (PredAHT) clinical prediction tool: A qualitative study with child protection professionals, *Child Abuse & Neglect* 81():pp. 192-205.
25. Neumayer, Katie *et al.* (2020) Validation of the "CHIIDA" and application for PICU triage in children with complicated mild traumatic brain injury. *Journal of pediatric surgery*, 55(7), pp. 1255-1259
26. Greenberg, J., Yan, Y., Carpenter, C., Lumba-Brown, A., Keller, M., Pineda, J., Brownson, R. and Limbrick, D. (2018) Development of the CIDSS2 Score for Children with Mild Head Trauma without Intracranial Injury. *Journal of Neurotrauma,* 35(22), pp. 2699-2707.
27. Pfeiffer, H., Smith, A., Kemp, M., Cowley, E., Cheek, A., Dalziel, S., Borland, S., O'Brien, S., Bonisch, M., Neutze, J., Oakley, E., Crowe, L., Hearps, S., Lyttle, M., Bressan, S. and Babl, F. (2018) External validation of the PediBIRN clinical prediction rule for abusive head trauma. *Paediatrics,* 141(5).
28. Hess, Erik *et al.*(2014) Effectiveness of the head CT choice decision aid in parents of children with minor head trauma: study protocol for a multicentre randomized trial. *Trials,* 15(1), pp. 253-253.
29. Unden, J. *et al.* (2018) External validation of the Scandinavian guidelines for management of minimal, mild and moderate head injuries in children, *BMC Medicine,* 16(176).
30. Schwartz, Jamie *et al.* (2020) Applying Pediatric Brain Injury Guidelines at a Level I Adult/Pediatric Safety-Net Trauma Center. *The Journal of surgical research,* 255, pp. 106-110
31. Yogo, N., Toida, C., Muguruma, T., Gakumazawa, M., Shinohara, M. and Takeuchi, I. (2021) Simplified Clinical Decision Rule Using Clinically Important Events for Risk Prediction in Pediatric Head Injury: A Retrospective Cohort Study, *Journal of Clinical Medicine,* 10(22).
32. Brittany M. Stopa*, et al.* (2019) Comparison of minor head trauma management in the emergency departments of a United States and Italian Children's hospital, *Italian Journal of Pediatrics,* 45(24).
33. Da Dalt, L. *et al*.(2006) Predictors of intracranial injuries in children after blunt head trauma. *European Journal of Paediatrics,* 165(3), pp. 142–8.
34. Buchanich, J. M. (2007). A clinical decision-making rule for mild head injury in children less than three years old (Doctoral dissertation, University of Pittsburgh).
35. Greenberg *et al*. (2021) Development and external validation of the KIIDS-TBI tool for managing children with mild traumatic brain injury and intracranial injuries. *Academic Emergency Medicine,* 28(12).
36. Bressan, Silvia *et al.* (2012) Implementation of adapted PECARN decision rule for children with minor head injury in the paediatric emergency department. *Academic emergency medicine: official journal of the Society for Academic Emergency Medicine*, 19(7), pp. 801-807.
37. Babl, F. E., Oakley, E., Dalziel, S.R., Crowe, L., Bressan, S. and Lyttle, M. (2018) Accuracy of clinician practice compared with three head injury decision rules in children: a prospective cohort study, Annals of Emergency Medicine, 71 (6).

For peer review only - http://bmjopen.bmj.com/site/about/guidelines.xhtml

38. Pfeiffer, H., Smith, A., Kemp, M., Cowley, E., Cheek, A., Dalziel, S., Borland, S., O'Brien, S., Bonisch, M., Neutze, J., Oakley, E., Crowe, L., Hearps, S., Lyttle, M., Bressan, S. and Babl, F. (2018) External validation of the PediBIRN clinical prediction rule for abusive head trauma. Paediatrics, 141(5).
39. Babl, F.E et al. (2014) Head injury decision rules in children. Lancet, 390, pp. 1488.
40. Gupta, M., William, M., Rodriguez, R. and Hendey, G. (2018) Validation of the Pediatric NEXUS II Head Computed Tomography Decision Instrument for Selective Imaging of Pediatric Patients with Blunt Head Trauma. *Academic Emergency Medicine,* 25(7), pp. 729-737.
41. Pickering, A., Harnan, S., Fitzgerald, P., Pandor, A. and Goodacre, S. (2011) Clinical decision rules for children with minor head injury: a systematic review, Archives of Disease in Childhood, 95(5).
42. Doll, E., Gong, P., Sowell, M. and Evanczyk, L. (2021) Post-traumatic Headache in Children and Adolescents, Current Pain and Headache Reports, 25(51).
43. Fordington, S. and Manford, M. (2020) A review of seizures and epilepsy following traumatic brain injury, Journal of Neurology, 267(10).
44. Bressan, S.B., Romanato, S., Mion, T., Zanconato, S. and Da Dalt, L. (2012) Implementation of adapted PECARN decision rule for children with minor head injury in the paediatric emergency department, Academic Emergency Medicine, 19 (7).
45. Hymel, K.P., Fingarson, A.K., Pierce, M.C., Kaczor, K., Makoroff, K.L. and Wang, M. (2022) External Validation of the PediBIRN Screening Tool for Abusive Head Trauma in Paediatric Emergency Department Settings, Paediatric Emergency Medicine, 38(6).
46. Ide, K., Uematsu, S., Tetsuhara, K., Yoshimura, S. and Kato, T. (2016) External Validation of the PECARN Head Trauma Prediction Rules in Japan, Academic Emergency Medicine, 24(3), pp. 308-314.
47. Weil, Z.M., Corrigan, J.D., Karelina, K. (2018) Alcohol use disorder and traumatic brain injury, Alcohol Research, 39(2), pp. 171-180.
48. Lin, H., Lin, T., Soo, K., Chen, C., Kuo, L., Lin, Y., Lee, W. and Lin, C. (2014) The Effect of Alcohol Intoxication on Mortality of Blunt Head Injury, Biomed Research International.
49. Kambal, M. A., Abou, E.M., Gadi, I, A., Boukai, A. A., Jurayyan, N.A. and Babiker, A.M. (2014). Managing traumatic brain injury in children: When do we need a computed tomography of the head? Sudanese Journal of Paediatrics, 14(1).
50. Meulepas, J.D. et al. (2019) Radiation Exposure from Paediatric CT scans and Subsequent Cancer Risk in the Netherlands. Journal of the National Cancer Institute, 111(3), pp. 256-263.
51. Proctor, A., Voss, S., Lyttle, M.D. and Benger, J. (2023) Assessment and management of children with head injury: practice and opinion, *Journal of Paramedic Practice,* 15(2).
52. Burns, E. (2016) Scalp Hematoma Characteristics Associated with Intracranial Injury in Paediatric Minor Head Injury. Academic Emergency Medicine, 23(5), pp. 577-583.
53. Araki, T., Yokota, H., and Morita, A. (2017) Paediatric traumatic brain injury: characteristic features, diagnosis, and management, Neurologia medico-chirurgica, 57(2), pp. 82-93.
54. Moritz, D., Ebbs, P. and Carver, H. (2020) Paramedic ethics, capacity and the treatment of vulnerable patients. Journal of Paramedic Practice.
55. Dayan, P.S., Holmes, J.F., Atabaki, S., Miskin, M. and Kupperman, N. (2014) Association of Traumatic Brain Injuries with Vomiting in Children with Blunt Head Trauma, Annals of Emergency Medicine, 63(6), pp. 657-665.
56. Borland, M.L., et al. (2018) Delayed presentations to emergency departments of children with head injury: a predict study. Annals of Emergency Medicine, 74(1), pp. 1-10.
57. Crowe, L., et al., The epidemiology of paediatric head injuries: data from a referral centre in Victoria, Australia. Journal of paediatrics and child health, 2009. 45(6): p. 346-350.

For peer review only - http://bmjopen.bmj.com/site/about/guidelines.xhtml

58. Harper, J.A., Klassen, T.P., Balshaw, R., Dyck, J. and Osmond, M.H. (2020) Characteristics of vomiting as a predictor of intracranial injury in paediatric minor head injury, Canadian Journal of Emergency Medicine, 22(6).
59. Lee, L.K., Monroe, D., Bachman, M.C., Glass, T.F., Mahajan, P.V., Cooper, A., Stanley, R.M., Miskin, M., Dayan, P.S., Holmes, J.F., Kuppermann, N. (2014) Traumatic Brain Injury (TBI) Working Group of Pediatric Emergency Care Applied Research Network (PECARN). Isolated loss of consciousness in children with minor blunt head trauma. JAMA Pediatr, 168(9), pp. 837-43.
60. Lockie et al. (2013) Triggers for head computed tomography following paediatric head injury: Comparison of physicians' reported practice and clinical decision rules, Emergency Medicine Australia, 25, pp. 75-82.
61. Fowler, J., Beovich, B. and Williams, B. (2018) Improving paramedic confidence with paediatric patients: A scoping review. 15(1).
62. Bressan, S., Monagle, P., Dalziel, S., Borland, M., Phillips, N., Kochar, A., Lyttle, M., Cheek, J., Neutze, j., Oakley, E., Dalton, S., Gilhotra, Y., Hearps, S., Furyk, J. and Babl, F. (2020) Risk of traumatic intracranial haemorrhage in children with bleeding disorders, Journal of Paediatrics and Child Health, 56(12), pp. 1839-2003.
63. Pandor, A., Goodacre, S., Harnan, S., Holmes, M., Pickering, A., Fitzgerald, P., Rees, A. and Stevenson, M. (2011) Diagnostic management strategies for adults and children with minor head injury: a systematic review and an economic evaluation, Health Technology Assessment. 15(27).
64. Lee, L., Dayan, P. Gerardi, M., Borgialli, d., Badawy, M., Callahan, J., Lillis, K., Stanley, R., Gorelick, M., Dong, L., Zuspan, S., Holmes, J. and Kupperman, N. (2011) Intracranial haemorrhage after blunt head trauma in children with bleeding disorders, The Journal of Paediatrics, 158(6), pp. 1003-1008.

WORD COUNT (excluding tables and references):

3364

KEYWORDS:

Systematic review; head injuries; paramedic; children.

PATIENT AND PUBLIC INVOLVEMENT:

Patients and public have been involved throughout this research. Both a Young Persons Advisory Group (YPAG) (children aged 9-16 years) and Parent Advisory Group (PAG) have supported this study and confirmed its importance for children, parents, and the wider health and care system.

CONFLICT OF INTEREST:

None

No financial relationships with any organisations that might have an interest in the submitted work in the previous three years, no other relationships or activities that could appear to have influenced the submitted work.

COPYRIGHT:

The Corresponding Author has the right to grant on behalf of all authors and does grant on behalf of all authors, an exclusive licence (or non-exclusive for government employees) on a worldwide basis to the BMJ Publishing Group Ltd to permit this article (if accepted) to be published in BMJ editions

For peer review only - http://bmjopen.bmj.com/site/about/guidelines.xhtml

and any other BMJPGL products and sublicenses such use and exploit all subsidiary rights, as set out in our licence

TRANSPARENCY STATEMENT:

This manuscript is an honest, accurate and transparent account of the study being reported. No important aspects of the study have been omitted and any discrepancies from the study as originally planned have been explained.

FUNDING SOURCE:

This work is supported by Health Education England/National Institute for Health Research Integrated Clinical and Practitioner (ICA) Programme's Doctoral Clinical and Practitioner Academic Fellowship (DCAF) Scheme. The views expressed are those of the authors and do not necessarily represent those of Health Education England, the National Institute for Health Research or Department of Health.

ETHICS APPROVAL:

Not required

DATA SHARING:

No additional data available

AUTOR CONTRIBUTOR STATEMENT:

JB, SV and ML supervised the work. AP wrote the protocol, conducted the review and drafted the manuscript with input from JB, SV and ML. JeB double screened the abstracts and full texts. PS helped to design the search terms and conduct the search. All authors interpreted and analysed the results. All authors discussed the results, contributed to and approved the final manuscript.

For peer review only

Figure 1.0 PRISMA flow diagram

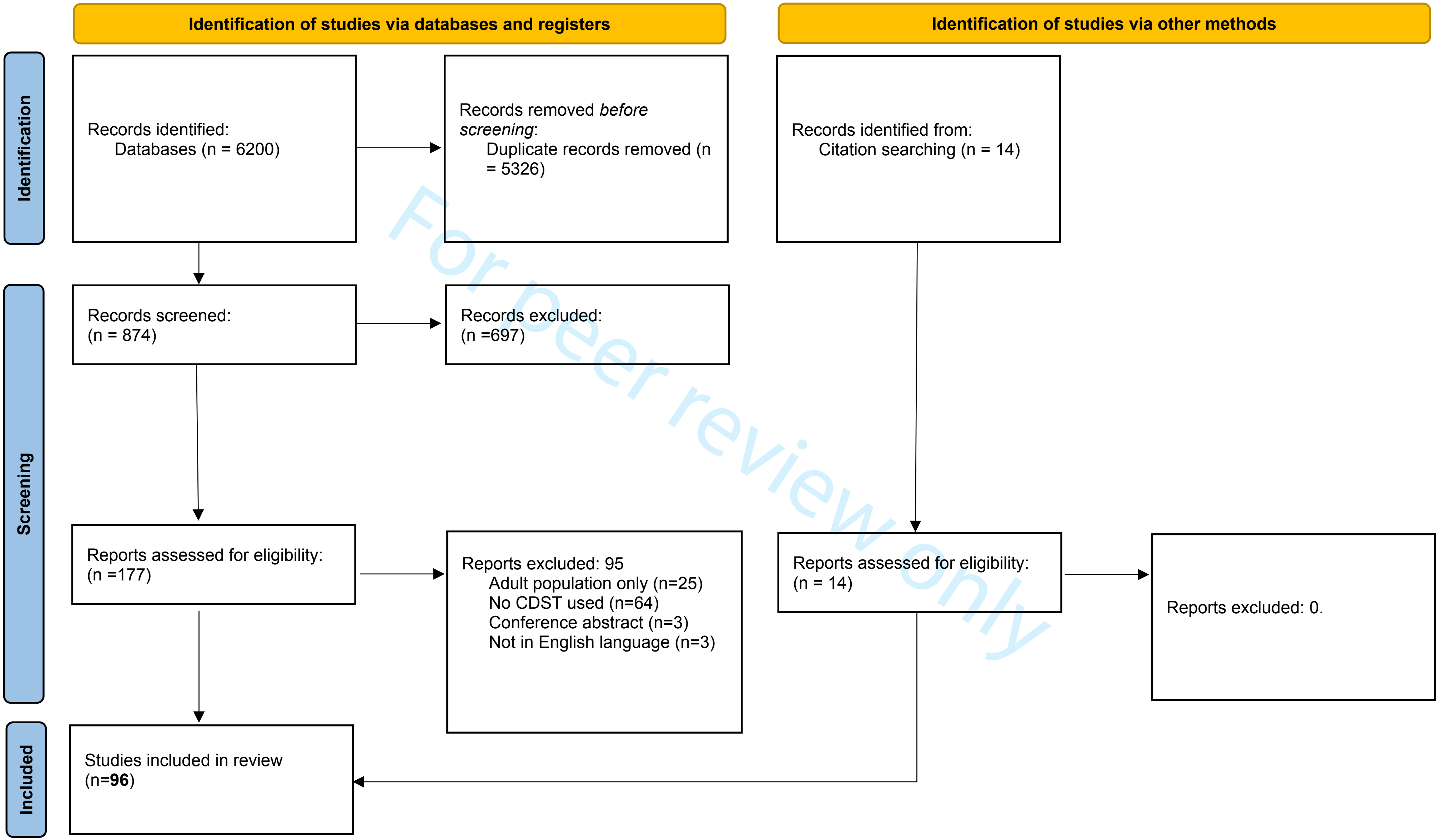

From: Page MJ, McKenzie JE, Bossuyt PM, Boutron I, Hoffmann TC, Mulrow CD, et al. The PRISMA 2020 statement: an updated guideline for reporting systematic reviews. BMJ 2021;372:n71. doi: 10.1136/bmj.n71. For more information, visit: http://www.prisma-statement.org/

For peer review only - http://bmjopen.bmj.com/site/about/guidelines.xhtml

CDRF Systematic Review Protocol V 3.0

Review Title:

Which elements of hospital-based clinical decision support tools for the assessment and management of children with head injury can be adapted for use by paramedics in pre-hospital care? A systematic mapping review and formal narrative synthesis.

Anticipated start date:

12 September 2022

Anticipated completion date:

12 March 2023

For peer review only

Named contact:

Alyesha Proctor

Alyesha.proctor@uwe.ac.uk

Organisational affiliation of the review:

University of the West of England

Review team members:

Alyesha Proctor, University of the West of England

Professor Jonathan Benger, University of the West of England

Professor Sarah Voss, University of the West of England

Dr Mark Lyttle, University of the West of England

Jedd Billing, University of the West of England

Julian Simpson, University of the West of England

Pauline Shaw, University of the West of England

Funding source:

HEE/NIHR Integrated Clinical and Practitioner (ICA) Programme’s Doctoral Clinical and Practitioner Academic Fellowship (DCAF) Scheme.

Conflicts of interest:

None

Review Question:

Which elements of hospital-based clinical decision support tools for the assessment and management of children with head injury can be adapted for use by paramedics in pre-hospital care?

Background:

The NHS (National Health Service) is under substantial pressure due to rising demand. A leading priority is to reduce the number of patients who are unnecessarily conveyed to an Emergency Department (ED) by ambulance. Children are frequent users of the ambulance service and ED, and head injury is common in children. However very few children with head injury who are conveyed to the ED need specialist treatment, and the vast majority could be managed safely at scene. Several decision tools exist to support clinical decision-making when a child presents to the ED with a minor head injury, yet there is no pre-hospital decision tool designed for use by paramedics to reduce unnecessary conveyance to hospital. None of the existing hospital-based tools can be implemented directly in pre-hospital care, because they are designed to advise clinicians of the need for a computed tomography (CT) scan, rather than ED conveyance. This gap is recognised in existing national guidance, and a pre-hospital tool to support effective decision-making by paramedics is required urgently.

Searches and information sources:

We will identify relevant studies by searching a selection of bibliographic databases, grey literature resources and clinical trial registries, checking reference lists of included studies and conducting forwards citation searching using included studies.

Databases:

- MEDLINE
- EMBASE
- PsycINFO
- CINAHL
- Cochrane Library
- AMED
- Open Grey
- Google scholar
- Scopus

The following clinical trial registries will be searched for unpublished studies:
ClinicalTrials.gov (https://ClinicalTrials.gov/)
ICTRP Search Portal (http://apps.who.int/trialsearch/)

To identify additional published, unpublished, and ongoing studies, the reference lists of all relevant studies (including existing systematic reviews) will be checked.

Experts:

For peer review only

CDRF Systematic Review Protocol V 3.0

Colleagues and experts in the field will be contacted for relevant 'grey literature' and unpublished reports, including:

- Dr Mark Lyttle (Consultant and Senior Research Fellow in Paediatric Emergency Medicine)
- Professor Franz Babl (Principal Research Fellow in Paediatric Emergency Medicine)
- Dr Patrick Aldridge (Consultant in Paediatric Emergency Medicine)

Justification of information sources:

The database resources have been selected since they include the key medical databases. OpenGrey will be used as the source for grey literature as it covers the relevant subject areas for this review and has open access to over 700,000 bibliographic references.
Expert colleagues will be identified and contacted to review the results of the systematic review literature searching and provide expert opinion on any relevant additional resources that have not been identified. Any eligible literature will be hand searched to ensure additional relevant literature are identified.

Search terms:

- A combination of MeSH terms and synonym text-strings/phrases will be used in the search strategy
- To be combined using Boolean operators
- Search terms have been developed iteratively by discussion with the research team and librarian

**Clinical decision support tool**	**Head injury**	**Children**	**Hospital**
Clinical decision tool	Minor head injury	Child	ED
Clinical decision rule	TBI (Traumatic brain injury)	Pediatric	Emergency Department
Diagnostic accuracy tool	Head trauma	Paediatric	A+E
Triage tool	Head wound	Baby	Accident and Emergency
Hospital-based tool	Intracranial injury	Babies	Emergency room
CDRs	CiTBI (clinical important traumatic brain injury)	Infant	Trauma centre
Intervention	ICI (intracranial injury)	Schoolchild	
Clinical decision score	CSII (clinically significant intracranial injury)	Adolescent	
Paediatric head injury predictive tool	Trivial head injury	Teenager	
Clinical Prediction Rule		Young person	
Clinical Prediction Tool			

- Search dates: January 1980 to the date of the search

For peer review only

- Must be in the English Language
- Searches will be re-run before final analysis

Search:

("Clinical decision support tool" OR "Clinical decision tool" OR "Clinical decision rule" OR "Diagnostic accuracy tool" OR "Triage tool" OR "Hospital-based tool" OR CDRs OR Intervention OR "Clinical decision score" OR "Decision Support Technique*" OR "paediatric head injury predictive tool") AND ("Head injury" OR "Minor head injury" OR "Traumatic brain injury" OR "Head trauma" OR "Head wound" OR "Intracranial injury" OR TBI OR CiTBI OR ICI OR CSII OR "trivial head injury") AND (Children OR Child OR Pediatric OR Paediatric OR Baby OR Babies OR Infant OR Schoolchild* OR Adolescent OR Teenage* OR "Young person") AND ("emergency department" OR "trauma centre" OR "ED" OR "A&E" OR "accident and emergency" OR "Emergency Service, Hospital" OR "emergency room")

Condition or domain being studied:

Systematic reviews that have considered paediatric head injury to date focus on identifying which tools are the most accurate in predicting the need for CT scanning in hospital, or the likelihood that a CT scan will show an abnormality that may need further intervention. This review will systematically identify and examine the existing in-hospital clinical decision tools for children with head injury to determine their ability to predict the need for hospital conveyance. This review will add to the evidence base with a focus on conveyance to hospital, rather than the need for imaging, and will provide an opportunity to complete a detailed and contemporary search of all available sources.

Participants/population:

**Inclusion Criteria**	**Exclusion Criteria**
Studies in the English Language	Non- English language
Primary quantitative, qualitative, and mixed methods research	
Children (under 19 years of age)	Adults (over 19 years of age)
Grey literature	
Date of publication 1980-present	
Any study that includes discussion of a hospital based clinical decision support rule/tool for assessing and managing children with head injury	
Worldwide	

- The papers reviewed will be limited to English language studies due to resource restrictions and the cost of translation. The systematic review will include a wide range of primary research, to capture all relevant evidence. A systematic search of the grey literature will allow any relevant unpublished research to be included for analysis. Limiting the search period to 1980 is likely to identify all, but a small minority of research completed before this time.

Comparator/Control:

Not applicable

URL to search strategy:

For peer review only

CDRF Systematic Review Protocol V 3.0

https://ezproxy.uwe.ac.uk/login?url=https://search.ebscohost.com/login.aspx?direct=true&db=cmedm&db=amed&db=jlh&db=psyh&bquery=(%26quot%3bClinical+decision+support+tool%26quot%3b+OR+%26quot%3bClinical+decision+tool%26quot%3b+OR+%26quot%3bClinical+decision+rule%26quot%3b+OR+%26quot%3bDiagnostic+accuracy+tool%26quot%3b+OR+%26quot%3bTriage+tool%26quot%3b+OR+%26quot%3bHospital-based+tool%26quot%3b+OR+CDRs+OR+Intervention+OR+%26quot%3bClinical+decision+score%26quot%3b+OR+%26quot%3bDecision+Support+Technique*%26quot%3b+OR+%26quot%3bpaediatric+head+injury+predictive+tool%26quot%3b)+AND+(%26quot%3bHead+injury%26quot%3b+OR+%26quot%3bMinor+head+injury%26quot%3b+OR+%26quot%3bTraumatic+brain+injury%26quot%3b+OR+%26quot%3bHead+trauma%26quot%3b+OR+%26quot%3bHead+wound%26quot%3b+OR+%26quot%3bIntracranial+injury%26quot%3b+OR+TBI+OR+CiTBI+OR+ICI+OR+CSII+OR+%26quot%3btrivial+head+injury%26quot%3b)+AND+(Children+OR+Child+OR+Pediatric+OR+Paediatric+OR+Baby+OR+Babies+OR+Infant+OR+Schoolchild*+OR+Adolescent+OR+Teenage*+OR+%26quot%3bYoung+person%26quot%3b)+AND+(%26quot%3bemergency+department%26quot%3b+OR+%26quot%3btrauma+center%26quot%3b+OR+%26quot%3bED%26quot%3b+OR+%26quot%3bA%26amp%3bE%26quot%3b+OR+%26quot%3baccident+and+emergency%26quot%3b+OR+%26quot%3bEmergency+Service%2c+Hospital%26quot%3b+OR+%26quot%3bemergency+room%26quot%3b)&cli0=DT1&clv0=198001-202212&type=1&searchMode=Standard&site=ehost-live

Types of study to be included:

Primary quantitative, qualitative, and mixed methods research will all be considered for inclusion. Other published research such as opinion pieces will be reviewed on a case-by-case basis.

Context:

All studies involving clinical decision support tools for the assessment and management of children with head injury will be considered.

Main outcome:

Phenomena of interest: to determine what elements of in-hospital clinical decision support tools for the assessment and management of children with head injury can be adapted for use in pre-hospital care. The findings will inform a new clinical decision support tool for use by paramedics in the pre-hospital setting, to support them in safely assessing and managing children with minor head injury at scene.

Additional outcome:

Not applicable

Data extraction:

Titles and abstracts will be double screened for relevance, and papers assessed against inclusion criteria by two researchers independently, using Covidence software. Any disagreements will be resolved through discussion with a senior researcher. The same process will be followed for full text screening. The output of the searches and screening will be reported in a PRISMA study flow diagram.

For peer review only

Extraction of data will analyse the ability to predict the need for hospital conveyance and determine applicability to paramedics in pre-hospital care. It is predicted that many of the clinical criteria within the tools will equally be applicable to both environments, however gaps in the criteria for hospital conveyance will be identified.

Extraction of data will involve systematically identifying all the in-hospital clinical decision support tools and then extract from these all the factors that are used in decision making. This will be presented in a detailed table. The researchers will then decide which of these criteria can be applied in pre-hospital care. It is predicted that some will clearly be applicable, some will not be applicable, and some will be potentially (or conditionally) applicable. We will then return to the table to identify which tools have the most applicable and the least applicable factors to identify the tool with the best fit for pre-hospital use. If none of the tools is a good fit, then something new will be created. Regardless, specific adaptation to pre-hospital care will also be required (in subsequent stages in this project).

Risk of bias/ Quality appraisal process:

n/a mapping review

Strategy for data synthesis:

All studies will be screened in Covidence, which is the primary screening and data extraction tool. Firstly, we plan to produce a descriptive 'map' of what elements/criteria from each hospital-based clinical decision support tool for the assessment and management of children with head injury can be adapted for use by paramedics in pre-hospital care, to predict the need for conveyance. Characteristics will be presented as descriptive variables identified through systematic coding. The analysis will be performed at the level of frequencies and simple cross-tabulations.

Secondly, to describe which criteria are suitable for paramedic use in pre-hospital care and which are not, and why, we will conduct a narrative synthesis. This will enable investigation of similarities and differences between the criteria in the tools, as well as exploration of the diagnostic accuracy of each tool for predicting the need for neurosurgical intervention, as this can be directly related to the need for conveyance. This will result in a summary of knowledge which will be used towards creating a new tool specific for use by paramedics to assess and manage children with head injury in pre-hospital care.

Dissemination plans:

This systematic review will be disseminated in a relevant open access peer reviewed journal. The results will also be communicated via social media, at research conferences, symposiums, and congresses to ensure access to a wide range of interested parties.

Keywords:

Systematic review; head injury; paramedic; children; clinical decision support tool

For peer review only

KIN 4400 Independent Research Study in Kinesiology | PRISMA 2020 Checklist

Section and Topic	Item #	Checklist item	Location where item is reported
**TITLE**			
Title	1	Identify the report as a literature review.	Page 1
**ABSTRACT**			
Abstract	2	Provide a structured summary including, as applicable: background; objectives; data sources; study eligibility criteria, participants, and interventions; study appraisal and synthesis methods; results; limitations; conclusions and implications of key findings.<br>See the PRISMA 2020 for Abstracts checklist for the complete list.	1 and 2
**INTRODUCTION**			
Rationale	3	Describe the rationale for the review in the context of existing knowledge, i.e., what is already known about your topic.	2 and 3
Objectives	4	Provide an explicit statement of the objective(s) or question(s) the review addresses with reference to participants, interventions, comparisons, outcomes, and study design (PICOS).	3
**METHODS**			
Eligibility criteria	5	Specify the inclusion and exclusion criteria for the review and how studies were grouped for the syntheses with study characteristics (e.g., PICOS, length of follow-up) and report characteristics (e.g., years considered, language, publication status) used as criteria for eligibility, giving rationale.	4
Information sources	6	Specify all databases, registers, websites, organisations, reference lists and other sources searched or consulted to identify studies. Specify the date when each source was last searched or consulted.	3
Search strategy	7	Present the full search strategies for all databases, registers and websites, including any filters and limits used.	3,4,5,6
Selection process	8	State the process for selecting studies (i.e., screening, eligibility).<br>Specify the methods used to decide whether a study met the inclusion criteria of the review, including how many reviewers screened each record and each report retrieved, whether they worked independently, and if applicable, details of automation tools used in the process.	3,4,5,6
Study risk of bias assessment	11	Specify the methods used to assess risk of bias in the included studies, including details of the tool(s) used, how many reviewers assessed each study and whether they worked independently, and if applicable, details of automation tools used in the process.	3,4,5
**RESULTS**			
Study selection	16a	Describe the results of the search and selection process, from the number of records identified in the search to the number of studies included in the review, ideally using a flow diagram.	6

The checklist has been adapted for KIN 4400 Independent Research Study in Kinesiology at the University of Guelph-Humber. Last updated: Dec 9, 2021

Adapted From: Page MJ, McKenzie JE, Bossuyt PM, Boutron I, Hoffmann TC, Mulrow CD, et al. The PRISMA 2020 statement: an updated guideline for reporting systematic reviews. BMJ 2021;372:n71. doi: 10.1136/bmj.n71 *Adapted From:* Page MJ, McKenzie JE, Bossuyt PM, Boutron I, Hoffmann TC, Mulrow CD, et al. The PRISMA 2020 statement: an updated guideline for reporting systematic reviews. BMJ 2021;372:n71. doi: 10.1136/bmj.n71

For more information, visit: http://www.prisma-statement.org/

For peer review only - http://bmjopen.bmj.com/site/about/guidelines.xhtml

KIN 4400 Independent Research Study in Kinesiology | PRISMA 2020 Checklist

Section and Topic	Item #	Checklist item	Location where item is reported
	16b	Cite studies that might appear to meet the inclusion criteria, but which were excluded, and explain why they were excluded.	6
Study characteristics	17	Cite each included study and present its characteristics (e.g., study size, PICOS, follow-up period).	6
Risk of bias in studies	18	Present assessments of risk of bias for each included study.	N/A
Results of individual studies	19	For all outcomes, present, for each study: (a) summary statistics for each group (where appropriate) and (b) an effect estimate and its precision (e.g. confidence/credible interval), ideally using structured tables or plots.	6,7,8,9,13,14,15,16
**DISCUSSION**			
Discussion	23a	Provide a general interpretation of the results in the context of other evidence.	20
	23b	Discuss any limitations of the evidence included in the review.	23
	23c	Discuss any limitations of the review processes used.	23
	23d	Discuss implications of the results for practice, policy, and future research.	23
**OTHER INFORMATION**			
Registration and protocol	24a	Provide registration information for the review, including register name and registration number, or state that the review was not registered.	3
	24b	Indicate where the review protocol can be accessed, or state that a protocol was not prepared.	3
	24c	Describe and explain any amendments to information provided at registration or in the protocol.	N/A
Support	25	Describe sources of financial or non-financial support for the review, and the role of the funders or sponsors in the review.	28
Competing interests	26	Declare any competing interests of review authors.	27
Availability of data, code, and other materials	27	Report which of the following are publicly available and where they can be found: template data collection forms; data extracted from included studies; data used for all analyses; analytic code; any other materials used in the review.	n/a

For peer review only

The checklist has been adapted for KIN 4400 Independent Research Study in Kinesiology at the University of Guelph-Humber. Last updated: Dec 9, 2021

Adapted From: Page MJ, McKenzie JE, Bossuyt PM, Boutron I, Hoffmann TC, Mulrow CD, et al. The PRISMA 2020 statement: an updated guideline for reporting systematic reviews. BMJ 2021;372:n71. doi: 10.1136/bmj.n71 *Adapted From:* Page MJ, McKenzie JE, Bossuyt PM, Boutron I, Hoffmann TC, Mulrow CD, et al. The PRISMA 2020 statement: an updated guideline for reporting systematic reviews. BMJ 2021;372:n71. doi: 10.1136/bmj.n71

For more information, visit: http://www.prisma-statement.org/

For peer review only - http://bmjopen.bmj.com/site/about/guidelines.xhtml

BMJ Open

Which elements of hospital-based clinical decision support tools for the assessment and management of children with head injury can be adapted for use by paramedics in pre-hospital care? A systematic mapping review and narrative synthesis.

Journal:	*BMJ Open*
Manuscript ID	bmjopen-2023-078363.R1
Article Type:	Original research
Date Submitted by the Author:	24-Oct-2023
Complete List of Authors:	Proctor, Alyesha; University of the West of England,<br>Lyttle, Mark; University of the West of England Bristol, Faculty of Health and Applied Sciences<br>Billing, Jedd; University of the West of England<br>Shaw, Pauline; University of the West of England<br>Simpson, Julian; University of the West of England<br>Voss, Sarah; University of the West of England, Health and Life Sciences<br>Benger, Jonathan; The University Hospitals NHS Foundation trust, Academic Department of Emergency care; The University of the West of England, Faculty of Health & Life Sciences
**Primary Subject Heading**:	Emergency medicine
Secondary Subject Heading:	Paediatrics
Keywords:	Systematic Review, PAEDIATRICS, ACCIDENT & EMERGENCY MEDICINE

SCHOLARONE™
Manuscripts

I, the Submitting Author has the right to grant and does grant on behalf of all authors of the Work (as defined in the below author licence), an exclusive licence and/or a non-exclusive licence for contributions from authors who are: i) UK Crown employees; ii) where BMJ has agreed a CC-BY licence shall apply, and/or iii) in accordance with the terms applicable for US Federal Government officers or employees acting as part of their official duties; on a worldwide, perpetual, irrevocable, royalty-free basis to BMJ Publishing Group Ltd ("BMJ") its licensees and where the relevant Journal is co-owned by BMJ to the co-owners of the Journal, to publish the Work in this journal and any other BMJ products and to exploit all rights, as set out in our licence.

The Submitting Author accepts and understands that any supply made under these terms is made by BMJ to the Submitting Author unless you are acting as an employee on behalf of your employer or a postgraduate student of an affiliated institution which is paying any applicable article publishing charge ("APC") for Open Access articles. Where the Submitting Author wishes to make the Work available on an Open Access basis (and intends to pay the relevant APC), the terms of reuse of such Open Access shall be governed by a Creative Commons licence – details of these licences and which Creative Commons licence will apply to this Work are set out in our licence referred to above.

Other than as permitted in any relevant BMJ Author's Self Archiving Policies, I confirm this Work has not been accepted for publication elsewhere, is not being considered for publication elsewhere and does not duplicate material already published. I confirm all authors consent to publication of this Work and authorise the granting of this licence.

For peer review only

TITLE:

Which elements of hospital-based clinical decision support tools for the assessment and management of children with head injury can be adapted for use by paramedics in pre-hospital care? A systematic mapping review and narrative synthesis.

AUTHORS:

Corresponding author: Alyesha Proctor[1]

Dr Mark D Lyttle[1]

Jedd Billing[1]

Pauline Shaw[1]

Julian Simpson[1]

Professor Sarah Voss[1]

Professor Jonathan Benger[1]

[1]Faculty of Health and Applied Sciences, University of the West of England, Glenside Campus (1H30), Blackberry Hill, Bristol, BS16 1DD, England

Alyesha.proctor@uwe.ac.uk (NIHR Clinical Doctoral Research Fellow, Advanced Paramedic Practitioner)

Mark.Lyttle@uwe.ac.uk (Senior Research Fellow, Consultant in Paediatric Emergency Medicine)

Jedd.billing@uwe.ac.uk (Senior Lecturer in Paramedic Science)

Pauline.shaw@uwe.ac.uk (Subject Librarian)

Julian.simpson@uwe.ac.uk (Programme Lead, Senior Lecturer in Paramedic Science)

Sarah.voss@uwe.ac.uk (Professor of Emergency Care)

Jonathan.benger@uwe.ac.uk (Professor of Emergency Care, Consultant in Emergency Medicine)

*Corresponding author address: Alyesha Proctor, Glenside Campus, Blackberry Hill, Stapleton, Bristol, BS16 1DD.

ABSTRACT:

Objective:

Hospital-based clinical decision tools support clinician decision making when a child presents to the Emergency Department with a head injury, particularly regarding computed tomography scanning. However, there is no decision tool to support pre-hospital clinicians in deciding which head injured children require conveyance to hospital. This study aims to identify clinical decision tools, or constituent elements, which may be adapted for use in pre-hospital care.

Design:

Systematic mapping review and narrative synthesis.

Data sources:

For peer review only

Searches were conducted using MEDLINE, EMBASE, PsycINFO, CINAHL and AMED.

Eligibility criteria:

Any quantitative, qualitative, mixed methods or systematic review research that included a clinical decision support tool for assessing and managing children with head injury.

Data extraction and synthesis:
We systematically identified all in-hospital clinical decision support tools and extracted from these the clinical criteria used in decision making. We complemented this with a narrative synthesis.

Results:
Following de-duplication 887 articles were identified. After screening titles and abstracts, 710 articles were excluded, leaving 177 full text articles. Of these, 95 were excluded, yielding 82 studies. A further 14 studies were identified in the literature after cross checking, totalling 96 analysed studies. 25 relevant in-hospital clinical decision tools were identified, encompassing 67 different clinical criteria, which grouped into 18 categories.

Conclusion:
Factors that should be considered for use in a clinical decision tool designed to support paramedics in the assessment and management of children with head injury, are: signs of skull fracture; a large, boggy or non-frontal scalp haematoma neurological deficit; Glasgow Coma Scale score less than 15; prolonged or worsening headache; prolonged loss of consciousness; post traumatic seizure; amnesia in older children; non-accidental injury; drug or alcohol use; less than one year old. Clinical criteria that require further investigation include mechanism of injury; clotting impairment/anticoagulation; vertigo; length of time of unconsciousness; number of vomits.

Strengths and limitations of the study:

- The review is highly inclusive, with a range of global study settings, including qualitative, quantitative, and mixed methods research.
- The review provided an opportunity to complete a detailed and contemporary search of all available sources and identified 25 in-hospital tools, compared to 14 in a previous systematic review.
- The role of mapping reviews is to provide a descriptive account of the published literature; therefore, this should be considered when assessing the findings of the overall evidence synthesis.
- A direct comparison of the clinical decision support tools is not possible because they address different questions, age groups, injury severities, and outcomes.

INTRODUCTION:

Healthcare systems internationally are under substantial pressure due to rising patient demand. A leading priority is to reduce the number of patients who are unnecessarily conveyed to an Emergency Department (ED) by ambulance [1]. Children frequently use the ambulance service and ED, and childhood head injury is common; approximately 800,000 head-injured children attend EDs each year in the UK [2]. Very few children with head injury who are conveyed to the ED by ambulance need specialist treatment, and the vast majority could be managed safely at scene.

For peer review only - http://bmjopen.bmj.com/site/about/guidelines.xhtml

Despite this, a national overview of head injury in children found that one-third of children in hospital with head injury were transported by ambulance, and 74% of those conveyed were non-serious, requiring no intervention [3].

Head injury guidance produced by the National Institute for Health and Care Excellence (NICE) [4] is of limited utility to paramedics for reasons including: a lack of validation in pre-hospital care; reference to patient observation over time which is not always possible; challenges in the assessment of amnesia in younger children; limited research evidence on which to base recommendations relating to ambulance non-conveyance [4].

There are several existing validated hospital-based decision support tools that clinicians use when a child presents to the ED with a minor head injury, and studies have demonstrated their effectiveness in aiding decision-making for both immediate discharge and computed tomography (CT) scanning [5] [6]. Currently, there are no pre-hospital decision tools designed to support paramedics in reducing unnecessary conveyance to the ED for head-injured children. None of the existing hospital-based tools can be implemented directly in pre-hospital care, since they are designed to support decision making on performing CT scanning, rather than ED conveyance.

AIM:

To systematically identify and examine clinical criteria within existing hospital-based clinical decision support tools for children with minor head injury, with a focus on determining their potential to aid decision making regarding hospital conveyance by paramedics.

METHODS:

PATIENT AND PUBLIC INVOLVEMENT:

Patients and public have been involved throughout this research. Both a Young Persons Advisory Group (YPAG) (children aged 9-16 years) and Parent Advisory Group (PAG) have supported this study and confirmed its importance for children, parents, and the wider health and care system.

Introduction:

Systematic mapping aims to collate, explain, and catalogue available evidence (primary and secondary) relating to a topic of interest, to identify a focus for more specific investigation [7]. It is a review methodology that is often used in healthcare research that aims to detail and categorise literature on a particular topic, with the intention of developing this into more comprehensive work [8]. This systematic mapping review was conducted in accordance with published methods [9] and is reported in line with the PRISMA statement [10]. It has not been registered with Prospero as it is a mapping review.

Patients and public have been involved throughout this research. Both a Young Persons Advisory Group (YPAG) (children aged 9-16 years) and Parent Advisory Group (PAG) have supported this study and confirmed its importance for children, parents, and the wider health and care system.

Search strategy:

Searches were conducted in the following databases, for articles published between January 1980 and August 2022 to ensure that all relevant contemporary research was included: MEDLINE, EMBASE, PsycINFO, CINAHL and AMED. A Google Scholar and a Web of Science search were undertaken to identify reports or proceedings not indexed in the above. A literature advisory group,

For peer review only - http://bmjopen.bmj.com/site/about/guidelines.xhtml

including experts in the field, was consulted to identify relevant grey literature and unpublished reports. Furthermore, to find additional published, unpublished, and ongoing studies, the reference lists of all relevant studies were examined.

Search terms were developed iteratively by discussion among the research team, patient and public representatives and a librarian, seeking a balance between comprehensiveness and focus. A combination of MeSH terms and synonym text-strings/phrases were used in the search strategy and were combined using Boolean operators. Updated searches were re-run before the final analysis. The search terms used can be seen in Table 1.

The search was limited to English language studies due to resource restrictions and the cost of translation, and only articles with full texts available were included. Studies that may have been relevant but did not include or refer to a clinical decision support tool for the assessment and management of head-injured children, were excluded; however, there were no restrictions on the types of study included. Eligibility criteria are presented in full in Table 2.

For peer review only

Table 1. Search terms:

Clinical decision support tool	Head injury	Children	Hospital
Clinical decision tool	Minor head injury	Child	ED
Clinical decision rule	TBI (Traumatic brain injury)	Pediatric	Emergency Department
Diagnostic accuracy tool	Head trauma	Paediatric	A+E
Triage tool	Head wound	Baby	Accident and Emergency
Hospital-based tool	Intracranial injury	Babies	Emergency room
CDRs	CiTBI (clinical important traumatic brain injury)	Infant	Trauma centre
Intervention	ICI (intracranial injury)	Schoolchild	
Clinical decision score	CSII (clinically significant intracranial injury)	Adolescent	
Paediatric head injury predictive tool	Trivial head injury	Teenager	
Clinical Prediction Rule		Young person	
Clinical Prediction Tool			
Clinical decision support tool (CDST)			

Table 2. Inclusion and exclusion criteria:

Inclusion Criteria	Exclusion Criteria
Studies in the English Language	Non- English language
Quantitative, qualitative, mixed methods research, systematic reviews	
Children (under 19 years of age)	Adult population (19 years or older)

For peer review only - http://bmjopen.bmj.com/site/about/guidelines.xhtml

Date of publication 1980-present	
Any study that includes a clinical decision support tool for assessing and managing children with head injury	Any study that did not include a clinical decision support tool for the assessment and management of head-injured children
International as long as in the English language	

Extracting, Coding, and Synthesising the Data:

Titles and abstracts were double screened for relevance and assessed for eligibility using Covidence software [11], following which full texts of included articles underwent review for eligibility. Any disagreements were resolved through discussion with a senior researcher. Reasons for exclusion were documented, a topic expert reviewed the final set of included papers to ensure relevant studies and tools had not been overlooked. Figure 1 shows the PRISMA flow diagram [10].

Data extraction included systematically identifying the title of each tool, the aim, derivation reference, supporting literature, conclusion, and key points relating to the content and format of a clinical decision support tool for paramedic use. An inductive coding frame was developed to map emerging concepts, which involved deriving codes from the data. Key messages/concepts in relation to predicting conveyance from all studies were extracted from the methods, results, and conclusion sections. This resulted in a visual synthesis and classification of available studies and tools. The data extraction tool was piloted using two included studies, and data were extracted by hand and cross-checked by another researcher.

Clinical criteria used in decision making were extracted from all tools; a summary map was produced providing a visual display of clinical criteria included in each tool. After independently producing pilot categories based on a sample of clinical decision support tools, the research team formed consensus on categories. The frequency of each clinical criterion was tabulated, and performance accuracy was recorded where reported. A narrative synthesis was subsequently developed using this framework.

Assessment of Quality:

No formal assessment of quality assessment was undertaken, as this is not required in mapping reviews [12]. Data extraction in mapping reviews describes the studies and their main methods but does not attempt a full data extraction and quality assessment [12].

<u>RESULTS:</u>

A total of 887 articles were identified after duplicates were removed. After screening titles and abstracts, 710 articles were excluded, which left 177 full text articles to be assessed for eligibility. Of these, 95 were excluded for reasons including: adult population only; not including a clinical decision support tool; not in the English language; and full text not available. Three articles were not included, as they were conference abstracts. Fourteen additional studies were identified through citation searching, resulting in 96 included studies (Figure 1).

[Figure 1. PRISMA flow diagram]

In total, 25 relevant in-hospital clinical decision support tools for the assessment and management of children with head injury were identified, displayed in Supplementary Table 1.

[Supplementary Table 1. Hospital based clinical decision support tools for the assessment and management of children with head injury].

For peer review only - http://bmjopen.bmj.com/site/about/guidelines.xhtml

Sixty-seven decision-making criteria were extracted across all tools. Several criteria related to the same clinical feature (for example, vomiting), however there were many different cut points (e.g., vomiting once only, vomiting twice, or vomiting three times). These 67 criteria were grouped into 18 clinical categories: vomiting; headache; loss of consciousness (LOC); seizure; amnesia; dizziness/vertigo; clotting impairment/anticoagulated; non-accidental injury; age; severe mechanism of injury; drug/alcohol use; signs of a skull fracture; haematoma; neurological deficit; altered mental status; Glasgow come score (GCS); drowsiness and well with no clinical concern.

Supplementary Table 2 shows which clinical categories are included within each hospital-based tool.

[Supplementary Table 2. Categories in each tool]

For peer review only

For peer review only - http://bmjopen.bmj.com/site/about/guidelines.xhtml

Of these clinical categories, the most frequent were skull fracture, loss of consciousness, and scalp haematoma. The most infrequently used included vertigo, suspicion of non-accidental injury and patient acting normal as per parent. Table 3 tabulates the frequency of each clinical criterion.

[Table 3. Frequency of each clinical category across all the tools].

Categories	CDST	Frequency (n=25)
Signs of any description of skull fracture<br><br>(Depressed, basal skull, palpable)	PECARN 18, PECARN 2, NEXUS 2, HIDATq, PALCHACK, CHALICE, CATCH, GUZEL, BUCHANICH, DA DALT, KLEMETTI, QUAYLE, ATABAKI, SNC Guideline, BIG-1, Head trauma EBG algorithm, GREENES, CHIDA, CIDSS2, PediBIRN, KIIDS-TBI CDS	**21 (84%)**
LOC	PECARN 18, PECARN 2, CHALICE, CATCH, NEXUS 2, ATABAKI, KLEMETTI, QUAYLE, HIDATq, GUZEL, DIETRICH, DA DALT, BUCHANICH, SNC Guideline, BIG-1, Novel simplified CDR, Head trauma EBG algorithm	**17 (68%)**
Scalp haematoma/trauma	PECARN 2, CHALICE, PALCHAK, KLEMETTI, NEXUS 2, GUZEL, HAYDEL, GREENES, CATCH, HIDATq, BUCHANICH, ATABAKI, SNC Guideline, Head trauma EBG algorithm, PredAHT, PediBIRN, KIIDS-TBI CDS	**17 (68%)**
Neurological deficit	CHALICE, NEXUS 2, HIDATq, GUZEL, DIETRICH, DA DALT, BUCHANICH, KLEMETTI, ATABAKI, QUAYLE, Head CT Choice, SNC Guidelines, BIG-1, Head trauma EBG algorithm, CIDSS2	**15 (60%)**
Behaviour change/ altered mental status	PECARN 18, PECARN 2, CATCH, HIDATq, PALCHAK, ATABAKI, BUCHANICH, DA DALT, KLEMETI, QUAYLE, NEXUS 2, Head CT Choice, SNC Guidelines, Novel simplified CDR, Head trauma EBG algorithm	**15 (60%)**
GCS	PECARN 18, PECARN 2, CHALICE, NEXUS 2, CATCH, HIDATq, ATABAKI, CHIDA, CIDSS2, Head CT Choice, SNC	**15 (60%)**

For peer review only - http://bmjopen.bmj.com/site/about/guidelines.xhtml

	Guideline, Novel simplified CDR, Head trauma EBG algorithm, DA DALT, KIIDS-TBI CDT	
Vomiting	CHALICE, NEXUS 2, PECARN 18, PALCHACK, HIDATq, HAYDEL, DIETRICH, BUCHANICH, DA DALT, Head CT Choice, SNC Guidelines, Head trauma EBG algorithm	**12 (48%)**
Headache	PECARN 18, CATCH, PALCHAK, GUZEL, DIETRICH, HAYDEL, ATABAKI, BUCHANICH, DA DALT, Head CT Choice, SNC Guidelines, Novel simplified CDR	**12 (48%)**
Seizure	CHALICE, HIDATq, HAYDEL, ATABAKI, QUAYLE, DIETRICH, GUZEL, PredAHT, CIDSS2, SNC Guideline, Novel simplified CDR, DA DALT.	**12 (48%)**
Severe mechanism of injury	PECARN 18, PECARN 2, GUZEL CHALICE, CATCH, HIDATq, ATABAKI, Novel simplified CDR, Head trauma EBG algorithm	**9 (36%)**
Drowsiness	CHALICE, HIDATq, DA DALT, PECARN 18, PECARN 2, NEXUS 2, ATABAKI, Head CT Choice	**8 (32%)**
Amnesia	CHALICE, CATCH, NEXUS 2, HIDATq, HAYDEL, ATABAKI, GUZEL, DA DALT	**8 (32%)**
Drug or alcohol use	HIDATq, HAYDEL, ATABAKI, CIDSS2, BIG-1	**5 (20%)**
Age	PECARN, HIDATq, ATABAKI, HAYDEL	**4 (16%)**
Clotting impairment/anticoagulated	NEXUS 2, HIDATq, SNC Guideline	**3 (12%)**
Dizziness/vertigo	ATABAKI, KLEMETI	**2 (8%)**
Non accidental injury	CHALICE, HIDATq,	**2 (8%)**
Patient alert and well with no clinical concern/acting normal as per parent	PECARN 2, HIDATq	**2 (8%)**

The mix of clinical criteria used, and their outcomes were similar but not identical for any of the tools. In addition, the derivation studies used different populations and sample sizes and therefore the performance accuracy of the tools relating to the potential to correctly rule out clinically significant brain injury in head-injured children were variable *(*Supplementary Table 3).

For peer review only - http://bmjopen.bmj.com/site/about/guidelines.xhtml

[Supplementary Table 3. Performance accuracy of each tool].

Most tools displayed high sensitivities (>90%), while specificities ranged from 15 to 87%. PECARN was the most widely externally validated, with positive conclusions drawn from research examining post-implementation of the tool [36]. However, PECARN results in a considerable number of normal scans being performed to identify one intracranial injury [37]. The HIDATq tool was designed for nurses to use in triage to safely discharge children with head injury before admission, which more closely resembles the aim of a pre-hospital tool designed to inform the need for hospital conveyance. Supplementary Table 4 displays a narrative synthesis of each clinical criteria within the tools.

[Supplementary Table 4. Narrative synthesis of clinical criteria].

DISCUSSION:

This systematic mapping review identified and examined clinical criteria within existing hospital-based clinical decision support tools for children with minor head injury to determine their utility in paramedic practice, to inform the need for hospital conveyance. There are additional factors which require consideration in operationalising a tool for pre-hospital use, including the purpose of existing clinical decision rules, and additional relevant clinical factors.

Challenges of selecting a tool for use in pre-hospital care:

None of the existing hospital-based clinical decision support tools can be translated directly into paramedic practice. No single hospital based clinical decision support tool included all 18 clinical criteria, and there are potentially additional criteria that are not included in any of the tools that may be useful specifically in pre-hospital care; however, this needs to be explored in subsequent work. Most hospital based clinical decision support tools are designed to identify children who need a CT scan; although, this is not actually the clinical end point of interest. The reason for doing a CT scan is to seek intracranial injury that requires neurosurgery. Arguably, if a child receives no active treatment, then a CT scan is unnecessary, even if it does show intracranial injury [49] especially as paediatric CT scanning results in a significantly increased lifetime risk of radiation induced fatal malignancy [50]. The point of an out-of-hospital clinical decision support tool designed for use by paramedics is to identify children who would benefit from hospital conveyance for CT scanning to seek intracranial injury requiring neurosurgical intervention. This is accepting that there are complicating issues specific to pre-hospital care such as local ambulance policy, time of day, time since injury, complex wound closure, safeguarding, parental concern, and parental capability, that need to be considered and may warrant conveyance [51].

All the existing hospital-based tools address diverse questions, age groups, injury severities, and outcomes and have been derived, evaluated, and validated in a different way. In comparison to CHALICE, CATCH and other hospital-based tools, PECARN identifies children with a very low risk of brain injury who *do not* need a scan, which is an important concept, because it considers who a clinician *does not* need to scan, rather than who they *do* [6]. PECARN was derived using two large multicentre cohorts with over 40,000 children and is extremely sensitive, particularly for infants. A tool designed for use by paramedics in the pre-hospital setting could follow this approach and identify children who *do not* need conveyance, due to sustaining a trivial or minor head injury that is very low risk of clinically significant brain injury, similarly to Kupperman's PECARN [6] rather than identifying those who *do* need conveyance, though this needs further consideration. The role of ambulance paramedics includes the ability to make autonomous decisions on the safe discharge of patients at scene to prevent unnecessary conveyance to the ED. This is similar to in-hospital clinicians whose role

For peer review only - http://bmjopen.bmj.com/site/about/guidelines.xhtml

it is to discharge patients from the ED when it is safe to do so. Therefore, the in-hospital clinical decision support tools for the assessment and management of children with head injury will share many similarities, particularly if the tools are being used in triage. PECARN is also the only tool that considers the age of the child from the beginning and thereafter asks different questions relevant to the child's age [6]. Paramedics have reported difficulty using the NICE head injury guidance [4] for this reason, as they could not ask younger children about amnesia, headache, and alcohol use [51].

What clinical criteria should be included:

All 18 clinical criteria identified in this systematic mapping review are important to consider for a pre-hospital tool designed to support paramedics in their assessment and management of children with head injury, however some may be difficult to operationalise in the pre-hospital setting. In addition, the hospital-based tools have varying cut off points for different clinical criteria and which descriptor a pre-hospital tool should use requires further consideration. It is important to acknowledge that all clinical decision tools should be used alongside clinical judgment.

Certainty of use:

It is clear from the evidence that some clinical predictors are significant for identifying a potentially clinically important traumatic brain injury following a head injury and should be included in a pre-hospital tool, such as a post-traumatic seizure [43]. Signs of a skull fracture was most likely to trigger a CT response over any other clinical criteria, since the risk of intracranial injury is significantly increased in the presence of a skull fracture. This is reflected in the NICE guidance for the management of head injury, which suggests a CT scan should be performed within an hour if there are signs of a skull fracture [4]. The tools indicated that specifically a 'large', or 'non-frontal' (particularly parietal) [19] or 'boggy' haematoma, especially in infants, is more indicative of significant intracranial injury, which is apparent in other research [52]. Under 6-month-olds have a higher likelihood of significant intracranial haemorrhage with haematomas [52]. Greenes and Schutzman (2001) [19] suggest that among asymptomatic head-injured infants, the risk of skull fracture and associated intracranial haemorrhage is correlated with both scalp haematoma size and location. In most ambulance trusts across the United Kingdom, local policy stipulates that any under 2-year-old should be conveyed to the ED, no matter how mild the presenting injury [51]. Abusive head trauma occurs more frequently in under 1's [53]; paramedics are in a unique position to identify suspected non accidental injury since they attend the patient's home environment often in a time of crisis and have a professional duty to protect vulnerable persons [54]. This evidence supports the recommendation of including 'infants' as a clinical predictor for a pre-hospital tool designed to aid decision making regarding conveyance, or to exclude under 1's entirely. Non accidental injury is only a clinical predictor in two in-hospital clinical decision tools, however; perhaps this is because it is seen as a "no-brainer" and children were often excluded from the study cohort, as they would always need a scan. A child with suspected non accidental injury should always be conveyed to the ED by paramedics. Abuse tools such as those derived by Pfeiffer [27] and Cowley [24] include certain clinical predictors that can help paramedics in recognising non accidental injury.

Clinical predictors that may need intervention:

The pre-hospital environment is very different to in-hospital and there are some clinical predictors that may warrant conveyance as they may need interventions that cannot be done pre-hospitably, such as observation and complex wound closure. In addition, there are factors that are outside of a paramedic's scope of practice that presents too much risk to discharge at scene, and therefore warrants conveyance.

For peer review only - http://bmjopen.bmj.com/site/about/guidelines.xhtml

The number of vomits was variable across the tools; research suggests that children who vomit after a head injury is less significant than in adults and they do not necessarily have a serious brain injury [55]. Other literature supports this, suggesting that clinically important traumatic brain injuries are uncommon in children presenting with head injury with vomiting and a management approach of observation without immediate CT may be appropriate [56]. However, a period of observation is not always practical or possible for paramedics in the pre-hospital setting. This was reported by paramedics in a survey exploring reasons why paramedics convey children with minor head injury to the ED [51]. However, most children with head injury (90%) who are admitted for observation are discharged without any further treatment being given [57]. Notably, *persistent* vomiting post head injury is indicative of a subdural haematoma or skull fracture; Harper *et al.* (2020) [58] supports this, concluding that discreet vomiting over four times was a significant risk factor for intracranial injury. Additionally, vomiting combined with other clinical criteria such as headache and altered mental status, can indicate a more serious head injury [56]. This theme is clear with other clinical predictors such as loss of consciousness; children with minor blunt head trauma presenting to the ED with *isolated* loss of consciousness are at very low risk for clinically significant brain injury and do not routinely require CT scanning [59], however if the loss of consciousness was associated with other clinical predictors, the risk is increased. Any clinical predictor present in isolation is less likely to indicate clinically significant traumatic brain injury than the presence of multiple risk criteria [56]. This is similar for mechanism of injury; evidence implies that mechanism of injury alone is less predictive of an abnormal brain CT, compared with other clinical predictors [60]. Despite this, most paramedics would convey a head-injured child based solely on mechanism, despite them appearing well with no clinical concern [51].

Fowler *et al.* (2018) [61] found that attending to paediatric patients evokes anxiety and discomfort amongst paramedics, which often led to reluctance to initiate treatment and poorer care. This includes unnecessary transport to the ED. Approximately half of paramedic respondents in a survey reported feeling 'not at all confident' with completing a neurological assessment on a child following a head injury [51], despite a focal neurological deficit being a strong indicator of intracranial injury [33]. GCS is inherently difficult to assess in a younger child, however a reduced GCS is a high-risk factor for clinically significant intracranial injury [41].

Clinical predictors that infrequently result in intracranial injury:

There is limited evidence to suggest vertigo as a standalone clinical predictor should be included in a decision tool for conveyance of children with head injury, and this needs further investigation [20]. Additionally, evidence is still conflicting regarding the risk of intracranial haemorrhage in children with a clotting impairment/anticoagulation, [62], despite this being a research priority since 2011 [63]. Bressan *et al.* (2020) [63] found that although children with a bleeding disorder who have sustained a head injury more often received a CT scan, their risk of intracranial haemorrhage was low. Their findings are echoed in other literature [64]; CTs are obtained twice as often in children with bleeding disorders, although intracranial haemorrhage occurred in only 1.1% of these patients, and they had symptoms that would have prompted the need for a CT anyway. New guidance from NICE [4] advocates a CT head scan within eight hours for children on anticoagulant treatment, or within an hour if they have a bleeding/clotting disorder, independently of other indications. However, only one out of 31 children on anticoagulants in Bressen *et als.* (2020) [62] and Lee *et als*. (2011) [64] study combined, was diagnosed with an intracranial haemorrhage, and that patient very obviously needed a CT from the outset. These clinical criteria need further consideration before being included in a pre-hospital tool for paramedic use.

<u>LIMITATIONS:</u>

For peer review only - http://bmjopen.bmj.com/site/about/guidelines.xhtml

The role of mapping reviews is to provide a descriptive account of the published literature; therefore, this should be considered when assessing the findings of the overall evidence synthesis. A direct comparison of the clinical decision tools is not possible because they address different questions, age groups, injury severities, and outcomes. It is recognised that none of the evidence is from the pre-hospital environment, since there are no existing out-of-hospital clinical decision support tools designed to support the assessment and management children with head injury, hence the need for this research. Therefore, the in-hospital publications of tools considering childhood injury is the only research available to draw conclusions from at this point. Elements of in-hospital research can be attributed to pre-hospital care since there are similarities, however it is acknowledged that further research is required with pre-hospital staff in the pre-hospital environment following this work. Although rigorous methods of identifying all in-hospital tools have been followed, it is possible that some studies have been overlooked. This is particularly the case for international studies since this review included English language articles only.

CONCLUSIONS AND FUTURE RESEARCH:

Clinical predictors that markedly increase the likelihood of neurosurgical intervention and should be considered for inclusion in a clinical decision support tool for use by paramedics to assess the need for conveyance are: signs of a skull fracture; a large, boggy or non-frontal haematoma (particularly in an infant); persistent discreet episodes of vomiting; a focal neurological deficit; GCS less than 15; a prolonged or worsening headache; a prolonged loss of consciousness; a post traumatic seizure; amnesia in older children; suspicion of non-accidental injury; drug or alcohol use and being under 1 year old. Clinical criteria that require further investigation include mechanism of injury; clotting impairment/anticoagulated; vertigo; length of time of loss of consciousness; number of vomits, and description of what constitutes a neurological deficit in younger children. Any clinical predictor present in isolation is unlikely to indicate clinically significant traumatic brain injury.

There are likely to be additional clinical criteria that are relevant to paramedic assessment and practice, which are not included in any of these tools. Such criteria would need to be identified and explored in future qualitative work involving interviewing paramedics about what influences them when deciding to convey children with minor head injury to the hospital. None of the existing hospital-based clinical decision support tools can be directly implemented into paramedic practice; however, elements from each of the tools can be adapted to create a new tool specific for paramedics in pre-hospital care. Future qualitative research is needed to investigate paramedics' thoughts on a clinical decision tool designed to support them in their assessment and management of head-injured children. Additionally, future research utilising Delphi methods is required to build consensus amongst study matter experts and users to enable the development of a new clinical decision tool to support paramedics in safely assessing and managing children with minor head injury.

CONFLICT OF INTEREST:

None

No financial relationships with any organisations that might have an interest in the submitted work in the previous three years, no other relationships or activities that could appear to have influenced the submitted work.

COPYRIGHT:

For peer review only - http://bmjopen.bmj.com/site/about/guidelines.xhtml

The Corresponding Author has the right to grant on behalf of all authors and does grant on behalf of all authors, an exclusive licence (or non-exclusive for government employees) on a worldwide basis to the BMJ Publishing Group Ltd to permit this article (if accepted) to be published in BMJ editions and any other BMJPGL products and sublicenses such use and exploit all subsidiary rights, as set out in our licence

TRANSPARENCY STATEMENT:

This manuscript is an honest, accurate and transparent account of the study being reported. No important aspects of the study have been omitted and any discrepancies from the study as originally planned have been explained.

FUNDING SOURCE:

This work is supported by Health Education England/National Institute for Health Research Integrated Clinical and Practitioner (ICA) Programme's Doctoral Clinical and Practitioner Academic Fellowship (DCAF) Scheme, award number 25215. The views expressed are those of the authors and do not necessarily represent those of Health Education England, the National Institute for Health Research or Department of Health.

ETHICS APPROVAL:

Not required

DATA SHARING:

No additional data available

AUTOR CONTRIBUTOR STATEMENT:

JB, SV and ML supervised the work. AP wrote the protocol, conducted the review, and drafted the manuscript with input from JB, SV, ML, JeB and JS. JeB double screened the abstracts and full texts. PS helped to design the search terms and conduct the search. All authors interpreted and analysed the results. All authors discussed the results, contributed to and approved the final manuscript.

REFERENCES:

1. NHS (2019) The NHS Long term Plan. Available at: https://www.longtermplan.nhs.uk/ [Accessed 30th January 2023].
2. Arora, R., White, E.N., Niedbala, D., Ravichandran, Y., Sethuraman, U. and Radovic, N. (2020) Reducing Computed Tomography Scan Utilization for Paediatric Minor Head Injury in the Emergency Department: A Quality Improvement Initiative. *Academic Emergency Medicine,* 28(6), pp. 655-665.
3. Healthcare Quality Improvement Partnership (2015) Traumatic head injury in children and young people: a national overview. Available at: https://www.hqip.org.uk/wp-content/uploads/2018/02/traumatic-head-injury-in-children-and-young-people-a-national-overview.pdf [Accessed 30th January 2023].
4. National Institute for Health and Care Excellence (2023) *Head Injury Management.* Available at: https://cks.nice.org.uk/topics/head-injury/management/ [Accessed 23 May 2023].
5. Aldridge, P., Castle, H., Phillips, C., Russel, E., Guerrero-Luduena, R. and Rout, R. (2020). Head home: a prospective cohort study of a nurse led paediatric head injury clinical decision tool at a district general hospital, Emergency Medical Journal, 37(11), pp. 680-685.

6. Kuppermann, N., *et al.* (2009) Identification of children at very low risk of clinically-important brain injuries after head trauma: A prospective cohort study. *The Lancet,* 374(9696), pp. 1160-1170.
7. Campbell, F., Tricco, A.C., Munn, Z., Pollock, D., Saran, A., Sutton, A., White, H. and Khalil, H. (2023) Mapping reviews, scoping reviews, and evidence and gap maps: the same but different – the big picture review family, *Systematic reviews*, 12(45).
8. Grant, M.J. and Booth, A. (2009) A typology of reviews: an analysis of 14 review types and associated methodologies. *Health Info Library Journal,* 26(91), pp.108-9.
9. Clapton, J., Rutter, D. and Sharif, N. (2009) *Social Care Institute for Excellence (SCIE) systematic mapping guidance.* Available at: https://www.scie.org.uk/publications/researchresources/rr03.pdf [Accessed 30 January 2023].
10. Page, M.J., Mckenzie, J.E., Bossuyt, P.M., Hoffman, T.C. and Mulrow, C.D. (2020) The PRISMA 2020 statement: an updated guideline for reporting systematic reviews. BMJ, 172(71).
11. Covidence systematic review software, Veritas Health Innovation, Melbourne, Australia. Available at www.covidence.org [Accessed 18th April 2023].
12. Cooper, D. (2016). What is a "mapping study?" *Journal of the Medical Library Association,* 104(1), pp. 76-78.
13. Dunning, J., Daly, J., Lomas, J., Lecky, F., Batchelor, J. and Mackway-Jones (2006). Derivation of the children's head injury algorithm for the prediction of important clinical events decision rule for head injury in children. *Archives of Disease in Childhood,* 91(11), pp. 885-91.
14. Osmond, M.H (2010) CATCH: a clinical decision rule for the use of computed tomography in children with minor head injury, *Canadian Medical Association Journal,* 182(4).
15. Oman, J. A., Cooper, R. J., Holmes, J. F., Viccellio, P., Nyce, A., Ross, S. E., ... & Mower, W. R. (2006). Predictive performance of a decision rule to predict need for computed tomography among children with blunt head trauma. *Pediatrics,* 117(2), pp. 238-246.
16. Palchak, M.J *et al.* (2003). A decision rule for identifying children at low risk for brain injuries after blunt head trauma, *Annals of Emergency Medicine*, 42(4), pp. 492-506
17. Haydel, M.J. and Shembekar, A. D. (2003) Prediction of intracranial injury in children aged five years and older with loss of consciousness after minor head injury due to nontrivial mechanisms, *Annals of Emergency Medicine,* 42(4).
18. Atabaki, S. M., Stiell, I. G., Bazarian, J. J., Sadow, K. E., Vu, T. T., Camarca, M. A., ... & Chamberlain, J. M. (2008). A clinical decision rule for cranial computed tomography in minor pediatric head trauma. *Archives of pediatrics and adolescent medicine,* 162(5), pp. 439-445
19. Greenes, D. and Schutzman, S. (2001) Clinical significance of scalp abnormalities in asymptomatic head-injured infants. *Paediatric Emergency Care,* 17(2), pp. 88-92.
20. Klemetti, S., Uhari, M., Pokka, T., & Rantala, H. (2009). Evaluation of decision rules for identifying serious consequences of traumatic head injuries in pediatric patients. Pediatric emergency care, 25(12), 811-815
21. Quayle, K. S., Jaffe, D. M., Kuppermann, N., Kaufman, B. A., Lee, B. C., Park, T. S., & McAlister, W. H. (1997). Diagnostic testing for acute head injury in children: when are head computed tomography and skull radiographs indicated?. *Pediatrics,* 99(5).
22. Dietrich, A. M., Bowman, M. J., Ginn-Pease, M. E., Kosnik, E., & King, D. R. (1993). Pediatric head injuries: can clinical factors reliably predict an abnormality on computed tomography?. *Annals of emergency medicine, 22*(10), pp. 1535-1540.
23. Güzel, A., Hiçdönmez, T., Temizöz, O., Aksu, B., Aylanç, H., & Karasalihoglu, S. (2009). Indications for brain computed tomography and hospital admission in pediatric patients

For peer review only - http://bmjopen.bmj.com/site/about/guidelines.xhtml

with minor head injury: how much can we rely upon clinical findings?. *Pediatric neurosurgery,* 45(4), pp. 262-270.

24. Cowley, Laura E *et al.* (2018) Acceptability of the Predicting Abusive Head Trauma (PredAHT) clinical prediction tool: A qualitative study with child protection professionals, *Child Abuse & Neglect* 81():pp. 192-205.
25. Neumayer, Katie *et al.* (2020) Validation of the "CHIIDA" and application for PICU triage in children with complicated mild traumatic brain injury. *Journal of pediatric surgery*, 55(7), pp. 1255-1259
26. Greenberg, J., Yan, Y., Carpenter, C., Lumba-Brown, A., Keller, M., Pineda, J., Brownson, R. and Limbrick, D. (2018) Development of the CIDSS2 Score for Children with Mild Head Trauma without Intracranial Injury. *Journal of Neurotrauma,* 35(22), pp. 2699-2707.
27. Pfeiffer, H., Smith, A., Kemp, M., Cowley, E., Cheek, A., Dalziel, S., Borland, S., O'Brien, S., Bonisch, M., Neutze, J., Oakley, E., Crowe, L., Hearps, S., Lyttle, M., Bressan, S. and Babl, F. (2018) External validation of the PediBIRN clinical prediction rule for abusive head trauma. *Paediatrics,* 141(5).
28. Hess, Erik *et al.*(2014) Effectiveness of the head CT choice decision aid in parents of children with minor head trauma: study protocol for a multicentre randomized trial. *Trials,* 15(1), pp. 253-253.
29. Unden, J. *et al.* (2018) External validation of the Scandinavian guidelines for management of minimal, mild and moderate head injuries in children, *BMC Medicine,* 16(176).
30. Schwartz, Jamie *et al.* (2020) Applying Pediatric Brain Injury Guidelines at a Level I Adult/Pediatric Safety-Net Trauma Center. *The Journal of surgical research,* 255, pp. 106-110
31. Yogo, N., Toida, C., Muguruma, T., Gakumazawa, M., Shinohara, M. and Takeuchi, I. (2021) Simplified Clinical Decision Rule Using Clinically Important Events for Risk Prediction in Pediatric Head Injury: A Retrospective Cohort Study, *Journal of Clinical Medicine,* 10(22).
32. Brittany M. Stopa*, et al.* (2019) Comparison of minor head trauma management in the emergency departments of a United States and Italian Children's hospital, *Italian Journal of Pediatrics,* 45(24).
33. Da Dalt, L. *et al*.(2006) Predictors of intracranial injuries in children after blunt head trauma. *European Journal of Paediatrics,* 165(3), pp. 142–8.
34. Buchanich, J. M. (2007). A clinical decision-making rule for mild head injury in children less than three years old (Doctoral dissertation, University of Pittsburgh).
35. Greenberg *et al*. (2021) Development and external validation of the KIIDS-TBI tool for managing children with mild traumatic brain injury and intracranial injuries. *Academic Emergency Medicine,* 28(12).
36. Bressan, Silvia *et al.* (2012) Implementation of adapted PECARN decision rule for children with minor head injury in the paediatric emergency department. *Academic emergency medicine: official journal of the Society for Academic Emergency Medicine*, 19(7), pp. 801-807.
37. Babl, F. E., Oakley, E., Dalziel, S.R., Crowe, L., Bressan, S. and Lyttle, M. (2018) Accuracy of clinician practice compared with three head injury decision rules in children: a prospective cohort study, Annals of Emergency Medicine, 71 (6).
38. Pfeiffer, H., Smith, A., Kemp, M., Cowley, E., Cheek, A., Dalziel, S., Borland, S., O'Brien, S., Bonisch, M., Neutze, J., Oakley, E., Crowe, L., Hearps, S., Lyttle, M., Bressan, S. and Babl, F. (2018) External validation of the PediBIRN clinical prediction rule for abusive head trauma. Paediatrics, 141(5).
39. Babl, F.E et al. (2014) Head injury decision rules in children. Lancet, 390, pp. 1488.

For peer review only

For peer review only - http://bmjopen.bmj.com/site/about/guidelines.xhtml

40. Gupta, M., William, M., Rodriguez, R. and Hendey, G. (2018) Validation of the Pediatric NEXUS II Head Computed Tomography Decision Instrument for Selective Imaging of Pediatric Patients with Blunt Head Trauma. *Academic Emergency Medicine,* 25(7), pp. 729-737.
41. Pickering, A., Harnan, S., Fitzgerald, P., Pandor, A. and Goodacre, S. (2011) Clinical decision rules for children with minor head injury: a systematic review, Archives of Disease in Childhood, 95(5).
42. Doll, E., Gong, P., Sowell, M. and Evanczyk, L. (2021) Post-traumatic Headache in Children and Adolescents, Current Pain and Headache Reports, 25(51).
43. Fordington, S. and Manford, M. (2020) A review of seizures and epilepsy following traumatic brain injury, Journal of Neurology, 267(10).
44. Bressan, S.B., Romanato, S., Mion, T., Zanconato, S. and Da Dalt, L. (2012) Implementation of adapted PECARN decision rule for children with minor head injury in the paediatric emergency department, Academic Emergency Medicine, 19 (7).
45. Hymel, K.P., Fingarson, A.K., Pierce, M.C., Kaczor, K., Makoroff, K.L. and Wang, M. (2022) External Validation of the PediBIRN Screening Tool for Abusive Head Trauma in Paediatric Emergency Department Settings, Paediatric Emergency Medicine, 38(6).
46. Ide, K., Uematsu, S., Tetsuhara, K., Yoshimura, S. and Kato, T. (2016) External Validation of the PECARN Head Trauma Prediction Rules in Japan, Academic Emergency Medicine, 24(3), pp. 308-314.
47. Weil, Z.M., Corrigan, J.D., Karelina, K. (2018) Alcohol use disorder and traumatic brain injury, Alcohol Research, 39(2), pp. 171-180.
48. Lin, H., Lin, T., Soo, K., Chen, C., Kuo, L., Lin, Y., Lee, W. and Lin, C. (2014) The Effect of Alcohol Intoxication on Mortality of Blunt Head Injury, Biomed Research International.
49. Kambal, M. A., Abou, E.M., Gadi, I, A., Boukai, A. A., Jurayyan, N.A. and Babiker, A.M. (2014). Managing traumatic brain injury in children: When do we need a computed tomography of the head? Sudanese Journal of Paediatrics, 14(1).
50. Meulepas, J.D. et al. (2019) Radiation Exposure from Paediatric CT scans and Subsequent Cancer Risk in the Netherlands. Journal of the National Cancer Institute, 111(3), pp. 256-263.
51. Proctor, A., Voss, S., Lyttle, M.D. and Benger, J. (2023) Assessment and management of children with head injury: practice and opinion, *Journal of Paramedic Practice,* 15(2).
52. Burns, E. (2016) Scalp Hematoma Characteristics Associated with Intracranial Injury in Paediatric Minor Head Injury. Academic Emergency Medicine, 23(5), pp. 577-583.
53. Araki, T., Yokota, H., and Morita, A. (2017) Paediatric traumatic brain injury: characteristic features, diagnosis, and management, Neurologia medico-chirurgica, 57(2), pp. 82-93.
54. Moritz, D., Ebbs, P. and Carver, H. (2020) Paramedic ethics, capacity and the treatment of vulnerable patients. Journal of Paramedic Practice.
55. Dayan, P.S., Holmes, J.F., Atabaki, S., Miskin, M. and Kupperman, N. (2014) Association of Traumatic Brain Injuries with Vomiting in Children with Blunt Head Trauma, Annals of Emergency Medicine, 63(6), pp. 657-665.
56. Borland, M.L., et al. (2018) Delayed presentations to emergency departments of children with head injury: a predict study. Annals of Emergency Medicine, 74(1), pp. 1-10.
57. Crowe, L., et al., The epidemiology of paediatric head injuries: data from a referral centre in Victoria, Australia. Journal of paediatrics and child health, 2009. 45(6): p. 346-350.
58. Harper, J.A., Klassen, T.P., Balshaw, R., Dyck, J. and Osmond, M.H. (2020) Characteristics of vomiting as a predictor of intracranial injury in paediatric minor head injury, Canadian Journal of Emergency Medicine, 22(6).
59. Lee, L.K., Monroe, D., Bachman, M.C., Glass, T.F., Mahajan, P.V., Cooper, A., Stanley, R.M., Miskin, M., Dayan, P.S., Holmes, J.F., Kuppermann, N. (2014) Traumatic Brain Injury (TBI)

For peer review only - http://bmjopen.bmj.com/site/about/guidelines.xhtml

Working Group of Pediatric Emergency Care Applied Research Network (PECARN). Isolated loss of consciousness in children with minor blunt head trauma. JAMA Pediatr, 168(9), pp. 837-43.

60. Lockie et al. (2013) Triggers for head computed tomography following paediatric head injury: Comparison of physicians' reported practice and clinical decision rules, Emergency Medicine Australia, 25, pp. 75-82.
61. Fowler, J., Beovich, B. and Williams, B. (2018) Improving paramedic confidence with paediatric patients: A scoping review. 15(1).
62. Bressan, S., Monagle, P., Dalziel, S., Borland, M., Phillips, N., Kochar, A., Lyttle, M., Cheek, J., Neutze, j., Oakley, E., Dalton, S., Gilhotra, Y., Hearps, S., Furyk, J. and Babl, F. (2020) Risk of traumatic intracranial haemorrhage in children with bleeding disorders, Journal of Paediatrics and Child Health, 56(12), pp. 1839-2003.
63. Pandor, A., Goodacre, S., Harnan, S., Holmes, M., Pickering, A., Fitzgerald, P., Rees, A. and Stevenson, M. (2011) Diagnostic management strategies for adults and children with minor head injury: a systematic review and an economic evaluation, Health Technology Assessment. 15(27).
64. Lee, L., Dayan, P. Gerardi, M., Borgialli, d., Badawy, M., Callahan, J., Lillis, K., Stanley, R., Gorelick, M., Dong, L., Zuspan, S., Holmes, J. and Kupperman, N. (2011) Intracranial haemorrhage after blunt head trauma in children with bleeding disorders, The Journal of Paediatrics, 158(6), pp. 1003-1008.

FIGURE LEGEND:

Figure 1- Prisma flow chart

WORD COUNT (excluding tables and references):

3,630

KEYWORDS:

Systematic review; head injuries; paramedic; children.

For peer review only - http://bmjopen.bmj.com/site/about/guidelines.xhtml

Figure 1.0 PRISMA flow diagram

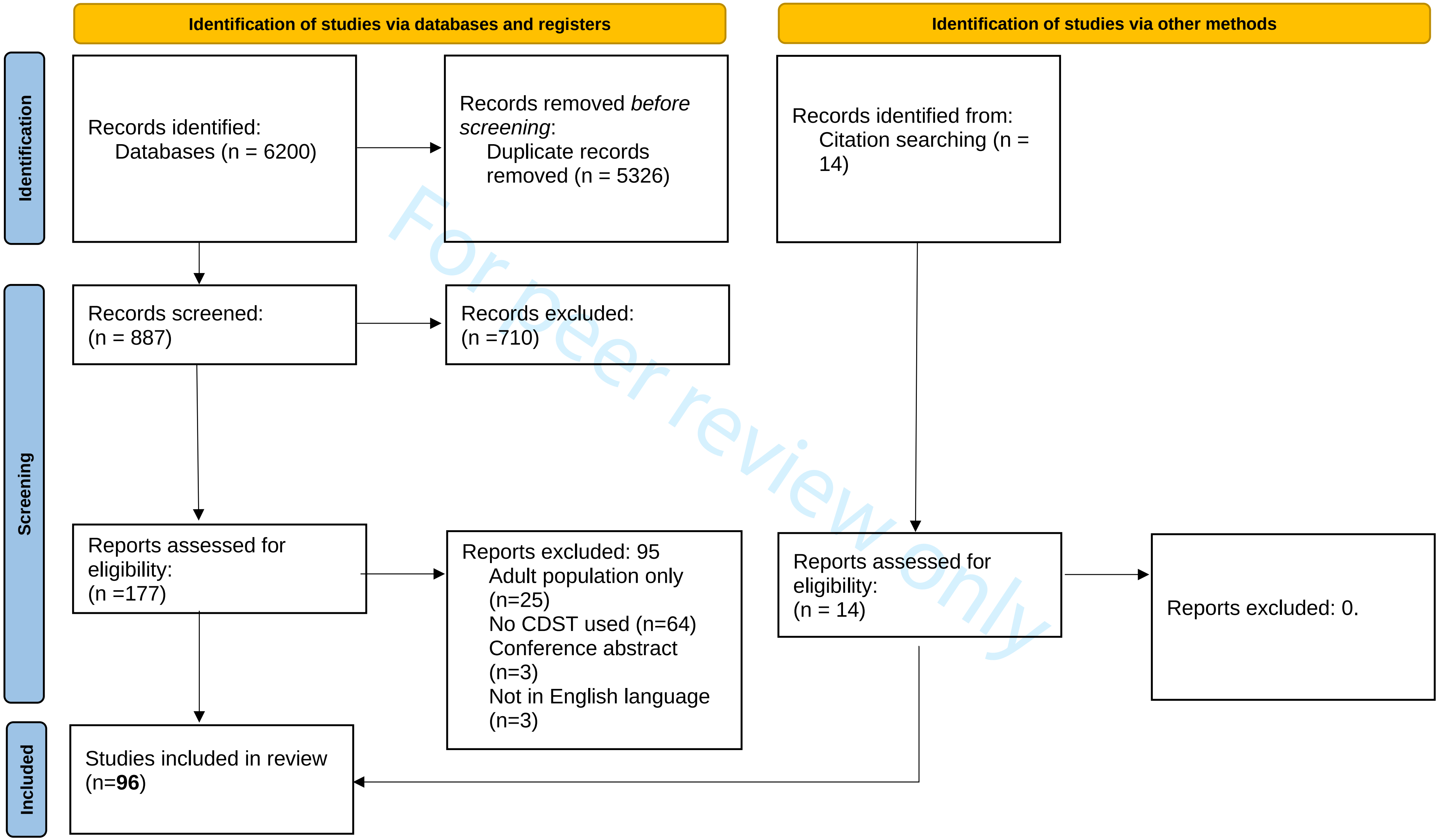

From: Page MJ, McKenzie JE, Bossuyt PM, Boutron I, Hoffmann TC, Mulrow CD, et al. The PRISMA 2020 statement: an updated guideline for reporting systematic reviews. BMJ 2021;372:n71. doi: 10.1136/bmj.n71. For more information, visit: http://www.prisma-statement.org/

For peer review only - http://bmjopen.bmj.com/site/about/guidelines.xhtml

Supplementary Table 1. Hospital based clinical decision support tools for the assessment and management of children with head injury:

Clinical Decision Support Tools	Full title	Reference
1. PECARN	Paediatric Emergency Care Applied Research Network rule.	[6] Kuppermann, N., *et al.* (2009) Identification of children at very low risk of clinically important brain injuries after head trauma: A prospective cohort study. *The Lancet,* 374(9696), pp. 1160-1170.
2. CHALICE	Children's Head injury Algorithm for the prediction of Important Clinical Events Rule.	[13] Dunning, J., Daly, J., Lomas, J., Lecky, F., Batchelor, J. and Mackway-Jones (2006). Derivation of the children's head injury algorithm for the prediction of important clinical events decision rule for head injury in children. *Archives of Disease in Childhood,* 91(11), pp. 885-91.
3. CATCH	Canadian Assessment of Tomography for Childhood Head injury Rule.	[14] Osmond, M.H. (2010) CATCH: a clinical decision rule for the use of computed tomography in children with minor head injury, *Canadian Medical Association Journal,* 182(4).
4. NEXUS II	National Emergency X-Ray Utilization Study.	[15] Oman, J. A., Cooper, R. J., Holmes, J. F., Viccellio, P., Nyce, A., Ross, S. E., ... & Mower, W. R. (2006). Predictive performance of a decision rule to predict need for computed tomography among children with blunt head trauma. *Pediatrics,* 117(2), pp. 238-246.
5. HIDATq	Head Injury Discharge At Triage questionnaire.	[5] Aldridge, P., Castle, H., Phillips, C., Russel, E., Guerrero-Luduena, R. and Rout, R. (2020). Head home: a prospective cohort study of a nurse led paediatric head injury clinical decision tool at a district general hospital, Emergency Medical Journal, 37(11), pp. 680-685.
6. PALCHAK	UC Davis Rule for Paediatric Head Trauma.	[16] Palchak, M.J *et al.* (2003). A decision rule for identifying children at low risk for brain injuries after blunt head trauma, *Annals of Emergency Medicine*, 42(4), pp. 492-506
7. HAYDEL	New Orleans/Charity Head Trauma/Injury Rule.	[17] Haydel, M.J. and Shembekar, A. D. (2003) Prediction of intracranial injury in children aged five years and older with loss of consciousness after minor head injury due to nontrivial mechanisms, *Annals of Emergency Medicine,* 42(4).
8. ATABAKI	No title	[18] Atabaki, S. M., Stiell, I. G., Bazarian, J. J., Sadow, K. E., Vu, T. T., Camarca, M. A., ... & Chamberlain, J. M. (2008). A clinical decision rule for cranial computed tomography in minor pediatric head trauma. *Archives of pediatrics and adolescent medicine,* 162(5), pp. 439-445

For peer review only

Supplementary Table 1. Hospital based clinical decision support tools for the assessment and management of children with head injury:

9. GREENES	No title	[19] Greenes, D. S., & Schutzman, S. A. (1999). Clinical indicators of intracranial injury in head-injured infants. *Pediatrics,* 104(4), pp. 861-867.
10. KLEMETTI	No title	[20] Klemetti, S., Uhari, M., Pokka, T., & Rantala, H. (2009). Evaluation of decision rules for identifying serious consequences of traumatic head injuries in pediatric patients. Pediatric emergency care, 25(12), 811-815
11. QUAYLE	No title	[21] Quayle, K. S., Jaffe, D. M., Kuppermann, N., Kaufman, B. A., Lee, B. C., Park, T. S., & McAlister, W. H. (1997). Diagnostic testing for acute head injury in children: when are head computed tomography and skull radiographs indicated? *Pediatrics,* 99(5).
12. DIETRICH	No title	[22] Dietrich, A. M., Bowman, M. J., Ginn-Pease, M. E., Kosnik, E., & King, D. R. (1993). Pediatric head injuries: can clinical factors reliably predict an abnormality on computed tomography? *Annals of emergency medicine, 22*(10), pp. 1535-1540.
13. GUZEL	No title	[23] Güzel, A., Hiçdönmez, T., Temizöz, O., Aksu, B., Aylanç, H., & Karasalihoglu, S. (2009). Indications for brain computed tomography and hospital admission in pediatric patients with minor head injury: how much can we rely upon clinical findings?. *Pediatric neurosurgery,* 45(4), pp. 262-270
14. PredAHT	Predicting Abusive Head Trauma	[24] Cowley, Laura E *et al.* (2018) Acceptability of the Predicting Abusive Head Trauma (PredAHT) clinical prediction tool: A qualitative study with child protection professionals, *Child Abuse & Neglect* 81, pp. 192-205.
15. CHIDA	Children's Intracranial Injury Decision Aid.	[25] Neumayer, Katie *et al.* (2020) Validation of the "CHIIDA" and application for PICU triage in children with complicated mild traumatic brain injury. *Journal of pediatric surgery*, 55(7), pp. 1255-1259
16. CIDSS2	Stands for:<br>Coma scale<br>Intoxication<br>Deficit (neurological)<br>Seizure<br>Skull fracture 2	[26] Greenberg, J., Yan, Y., Carpenter, C., Lumba-Brown, A., Keller, M., Pineda, J., Brownson, R. and Limbrick, D. (2018) Development of the $CIDSS_2$ Score for Children with Mild Head Trauma without Intracranial Injury. *Journal of Neurotrauma,* 35(22), pp. 2699-2707.
17. PediBIRN	Paediatric Brain Injury Research Network	[27] Pfeiffer, H., Smith, A., Kemp, M., Cowley, E., Cheek, A., Dalziel, S., Borland, S., O'Brien, S., Bonisch, M., Neutze, J., Oakley, E., Crowe, L., Hearps, S., Lyttle, M., Bressan, S. and Babl, F. (2018) External validation of the PediBIRN clinical prediction rule for abusive head trauma. *Paediatrics,* 141(5).

For peer review only

Supplementary Table 1. Hospital based clinical decision support tools for the assessment and management of children with head injury:

18. Head CT Choice	Head Computed Tomography Choice	[28] Hess, Erik *et al.* (2014) Effectiveness of the head CT choice decision aid in parents of children with minor head trauma: study protocol for a multicentre randomized trial. *Trials,* 15(1), pp. 253-253
19. SNC Tool	The Scandinavian Neurotrauma Committee Guidance	[29] Unden, J. *et al.* (2018) External validation of the Scandinavian guidelines for management of minimal, mild and moderate head injuries in children, *BMC Medicine,* 16(176).
20. BIG-1	Brain Injury Guidelines	[30] Schwartz, Jamie *et al.* (2020) Applying Pediatric Brain Injury Guidelines at a Level I Adult/Pediatric Safety-Net Trauma Center. *The Journal of surgical research,* 255, pp. 106-110
21. Novel Simplified CDR	Novel Simplified Clinical Decision Rule	[31] Yogo, N., Toida, C., Muguruma, T., Gakumazawa, M., Shinohara, M. and Takeuchi, I. (2021) Simplified Clinical Decision Rule Using Clinically Important Events for Risk Prediction in Pediatric Head Injury: A Retrospective Cohort Study, *Journal of Clinical Medicine,* 10(22).
22. Head Trauma EBG Algorithm	Head Trauma EBG Algorithm	[32] Brittany M. Stopa*, et al.* (2019) Comparison of minor head trauma management in the emergency departments of a United States and Italian Children's hospital, *Italian Journal of Pediatrics,* 45(24).
23. Da Dalt	No title	[33] Da Dalt, L. *et al*. (2006) Predictors of intracranial injuries in children after blunt head trauma. *European Journal of Paediatrics,* 165(3), pp. 142–8.
24. Buchanich	No title	[34] Buchanich, J. M. (2007). A clinical decision-making rule for mild head injury in children less than three years old (Doctoral dissertation, University of Pittsburgh).
25. KIIDS-TBI- CDS	The Kids Intracranial Injury Decision Support tool for Traumatic Brain Injury	[35] Greenberg *et al*. (2021) Development and external validation of the KIIDS-TBI tool for managing children with mild traumatic brain injury and intracranial injuries. *Academic Emergency Medicine,* 28(12).

For peer review only

Supplementary Table 2. Categories in each tool

	Signs of Skull fracture	Scalp Haematoma	Vomiting	Neuro Deficit	Headache	LOC	AMS	GCS	Seizure	Severe mechanism of injury	Drowsy	Amnesia	Vertigo	Non accidental injury	Clotting impairment/ anticoagulated	Drug/ alcohol use	Patient alert and well	Age
**PECARN (2-18)**	YES	NO	YES	NO	YES	YES	YES	YES	NO	YES	YES	NO	NO	NO	NO	NO	NO	NO
**PECARN (<2)**	YES	YES	NO	NO	NO	YES	YES	YES	NO	YES	YES	NO	NO	NO	NO	NO	YES	YES
**CHALICE**	YES	YES	YES	YES	NO	YES	NO	YES	YES	YES	YES	YES	NO	YES	NO	NO	NO	NO
**CATCH**	YES	YES	NO	NO	YES	YES	YES	YES	NO	YES	NO	YES	NO	NO	NO	NO	NO	NO
**NEXUS 2**	YES	YES	YES	YES	NO	YES	YES	YES	NO	NO	YES	YES	NO	NO	YES	NO	NO	NO
**HIDATq**	YES	YES	YES	YES	NO	YES	YES	YES	YES	YES	YES	YES	NO	YES	YES	NO	YES	YES
**PALCHACK**	YES	YES	YES	NO	YES	NO	YES	NO	NO	NO	NO	NO	NO	NO	NO	NO	NO	NO
**HAYDEL**	NO	YES	YES	NO	YES	NO	NO	NO	YES	NO	NO	YES	NO	NO	NO	YES	NO	YES
**ATABAKI**	YES	YES	YES	YES	YES	YES	YES	YES	YES	YES	YES	YES	YES	NO	NO	YES	NO	YES
**GREENES**	YES	YES	NO	NO	NO	NO	NO	NO	NO	NO	NO	NO	NO	NO	NO	NO	NO	NO
**KLEMETTI**	YES	YES	NO	YES	NO	YES	YES	NO	NO	NO	NO	NO	YES	NO	NO	NO	NO	NO
**QUAYLE**	YES	NO	NO	YES	NO	YES	YES	NO	YES	NO	NO	NO	NO	NO	NO	NO	NO	NO
**DIETRICH**	NO	NO	YES	YES	YES	YES	NO	NO	YES	NO	NO	NO	NO	NO	NO	NO	NO	NO
**GUZEL**	YES	YES	NO	YES	YES	YES	NO	NO	YES	YES	NO	YES	NO	NO	NO	NO	NO	NO
**PredAHT**	NO	YES	NO	NO	NO	NO	NO	NO	YES	NO	NO	NO	NO	NO	NO	NO	NO	NO
**CHIDA**	YES	NO	NO	NO	NO	NO	NO	YES	NO	NO	NO	NO	NO	NO	NO	NO	NO	NO
**CIDSS2**	YES	NO	NO	YES	NO	NO	NO	YES	YES	NO	NO	NO	NO	NO	NO	YES	NO	NO
**PediBIRN**	YES	YES	NO	NO	NO	NO	NO	NO	NO	NO	NO	NO	NO	NO	NO	NO	NO	NO
**HEAD CT CHOICE**	NO	NO	YES	YES	YES	NO	YES	YES	NO	NO	YES	NO	NO	NO	NO	NO	NO	NO
**SNC GUIDELINE**	YES	YES	YES	YES	YES	YES	YES	YES	YES	NO	NO	NO	NO	NO	YES	NO	NO	NO
**BIG-1**	YES	NO	NO	YES	NO	YES	NO	NO	NO	NO	NO	NO	NO	NO	NO	YES	NO	NO
**NOVEL CDR**	NO	NO	NO	NO	YES	YES	YES	YES	YES	YES	NO	NO	NO	NO	NO	NO	NO	NO
**HEAD TRAUMA EBG**	YES	YES	YES	YES	NO	YES	YES	YES	NO	YES	NO	NO	NO	NO	NO	NO	NO	YES
**DA DALT**	YES	NO	YES	YES	YES	YES	YES	YES	YES	NO	YES	YES	NO	NO	NO	NO	NO	NO
**BUCHANIC**	YES	YES	YES	YES	YES	NO	YES	NO	NO	NO	NO	NO	NO	NO	NO	NO	NO	NO
**KIIDS-TBI CDS**	YES	YES	NO	NO	NO	NO	NO	YES	NO	NO	NO	NO	NO	NO	NO	NO	NO	NO

Supplementary Table 3. Performance accuracy of each tool.

Name of tool	Internal validity	External validity	Implementation/ evaluation	Performance accuracy
**PECARN (under 2)**	Positive evidence	Positive evidence	Mixed evidence supporting potential effect.<br><br>Mixed evidence supporting positive conclusion for implementation.	Sensitivity: 100%<br><br>Specificity: between 53% and 60%
**PECARN (2-18)**	Positive evidence	Positive evidence	Mixed evidence supporting potential effect.<br><br>Mixed evidence supporting positive conclusion for implementation.	Sensitivity: 96.8%<br><br>Specificity: between 53% and 60%
**CHALICE**	Positive evidence	Positive evidence	Mixed evidence supporting positive conclusion for implementation.	Sensitivity: 98%<br><br>Specificity: 86%
**CATCH**	Positive evidence	Positive evidence	Not evaluated for usability, potential effect, or post implementation impact.	Sensitivity: 98%<br><br>Specificity: 50%
**NEXUS II**	Positive evidence	Mixed evidence supporting positive conclusion	Not evaluated for usability, potential effect, or post implementation impact.	Sensitivity: 99%<br><br>Specificity: 15%
**HIDATq**	Positive evidence	Not reported	Not evaluated in a published study after development	Negative predictive value for CT was 100% for intracranial injury. The

For peer review only

Supplementary Table 3. Performance accuracy of each tool.

				positive predictive value of the tool was low.
**PALCHACK**	Positive evidence	Positive evidence	Not evaluated in a published study after development	Sensitivity: 100%<br><br>Specificity: 46%
**HAYDEL**	Positive evidence	Not reported	Not evaluated in a published study after development	Sensitivity: 100%<br><br>Specificity: 24%
**ATABAKI**	Positive evidence	Not reported	Not evaluated in a published study after development	Sensitivity: 100%<br><br>Specificity: 46%
**BUCHANICH**	Positive evidence	Not reported	Not evaluated in a published study after development	Sensitivity: 100%<br><br>Specificity: 40%
**DA DALT**	Developed but not tested for internal validity	Not reported	Not evaluated in a published study after development	Sensitivity: 100%<br><br>Specificity: 87%
**GREENES**	Developed but not tested for internal validity	Not reported	Not evaluated in a published study after development	Sensitivity: 53%<br><br>Specificity: 72%
**KLEMETTI**	Developed but not tested for internal validity	Not reported	Not evaluated in a published study after development	Sensitivity: 94%<br><br>Specificity: 29%
**QUAYLE**	Developed but not tested for internal validity	Not reported	Not evaluated in a published study after development	Sensitivity: 44%<br><br>Specificity: 85%

For peer review only

Supplementary Table 3. Performance accuracy of each tool.

**DIETRICH**	Developed but not tested for internal validity	Not reported	Not evaluated in a published study after development	Sensitivity: 100%<br><br>Specificity: 17%
**GUZEL**	Developed but not tested for internal validity	Not reported	Not evaluated in a published study after development	Sensitivity: 69%<br><br>Specificity: 43%
**PredAHT 2**	Positive evidence	Positive evidence	Not evaluated for usability, potential effect, or post implementation impact.	Sensitivity: 72%<br><br>Specificity: 86%
**CHIDA**	Negative evidence	Not reported	Not evaluated in a published study after development	Sensitivity: 94%<br><br>Specificity: 69%
**CIDSS2**	Positive evidence	Not reported	Not evaluated in a published study after development	Sensitivity: 51%<br><br>Specificity: 96%
**PediBIRN**	Positive evidence	Positive evidence	Not evaluated in a published study after development	Sensitivity: 96%<br><br>Specificity: 29%

For peer review only

Supplementary Table 3. Performance accuracy of each tool.

**Head CT Choice**	Positive evidence	Not reported	Not evaluated in a published study after development	Not reported
**SNC guideline**	Positive evidence	Positive evidence	Not evaluated in a published study after development	Sensitivity: 95%<br><br>Specificity: 59%
**BIG-1**	Positive evidence	Not reported	Not evaluated in a published study after development	Not reported
**Novel simplified CDR**	Positive evidence	Not reported	Not evaluated in a published study after development	Sensitivity: 80%<br><br>Specificity: 51%
**Head trauma EBG algorithm**	Positive evidence	Not reported	Not evaluated in a published study after development	Not reported
**KIIDS-TBI CDS**	Positive evidence	Positive evidence	Not evaluated in a published study after development	Sensitivity: 100%<br><br>Specificity: 26%

For peer review only

Supplementary Table 3. Performance accuracy of each tool.

For peer review only

Supplementary Table 4. Narrative synthesis of clinical criteria:

	Narrative description of findings
**Signs of skull fracture**	This was the most frequently used clinical decision-making criterion (21/25) (84%). Variability of terms included: open or depressed skull fracture, basal skull fracture, tense fontanelle, palpable and unclear skull fracture. The only tools that do not include signs of skull fracture are HAYDEL, DIETRICH, PredAHT and Novel Simplified CDR. All three of PECARN, CHALICE and CATCH (the three most widely validated tools) included signs of skull fracture. PredAHT does not include signs of skull fracture despite the aim of this tool being to identify abusive head trauma. In contrast, PediBIRN which aims to identify children with abusive head trauma does include signs of skull fracture as a clinical predictor [38]. The KIIDS-TBI CDS Tool concluded that a depressed skull fracture was a major risk factor for significant brain injury [35]. Babl, *et al.* (2014) [39] and Da Dalt *et al.* (2006) [33] found that a basal, open or depressed skull fracture was most likely to trigger a CT response out of any other clinical criteria. Similarly, Quayle *et al.* (1997) [21] concluded that the relative risk for intracranial injury is increased almost fourfold in the presence of a skull fracture, although the absence of skull fracture does not rule out intracranial injury. The $CIDSS_2$ score [26], a tool to help physicians to identify the minority of children with minor traumatic brain injury at increased risk for extended inpatient stay, uses a scoring system; all clinical criteria score 1, apart from signs of skull fracture which scores 2, suggesting it is deemed the most significant clinical predictor of significant brain injury. Many of the tools, such as NEXUS 2, describe signs and symptoms of a skull fracture, rather than explicitly stating 'signs of a skull fracture'. For example: periorbital or periauricular ecchymoses, haemotympanum, and clear fluid from ears or nose [40].
**Scalp haematoma**	Scalp haematoma is used in 17 of the tools identified. However, many specify 'non-frontal' haematomas only, such as the NEXUS 2 tool [40]. Others (GREENES) support inclusion of non-frontal haematomas only; the authors found that non-frontal scalp haematomas had the strongest association with presence of intracranial haemorrhage [19]. The authors of PALCHAK concluded a scalp haematoma is more significant in children under 2 years [16]. The HIDATq tool does not stipulate location of haematoma, however the authors do specify a size (over 5cm) [5]. Similarly, CHALICE only specifies size, which is also over 5cm, but this is for under 1-year olds only [13]. PECARN clearly specifies 'temporal, occipital or parietal haematoma', and this is for the under 2-year-old tool [6]. The CATCH tool includes 'large boggy scalp haematoma', however it is unclear what constitutes 'large' as it does not specify what size or the location. Most excluded haematomas to the face.
**Loss of consciousness**	Loss of consciousness (LOC) has different cut points across the identified clinical decision tools, including 'any loss of consciousness', 'loss of consciousness for over 5 seconds' and 'loss of consciousness for over 5 minutes'. It is the second most frequently used clinical predictor, appearing in 17 of the 25 clinical decision tools. The authors of PALCHAK [16] found that the risk of a traumatic brain injury was higher in those with a history of loss of consciousness. However, in those children with an isolated loss of consciousness there were no traumatic brain injuries. This is recognised in Kupperman *et als.* (2009) derivation study for PECARN [6]; the risk of clinically important traumatic brain injury was substantially lower than 1% in children with isolated loss of consciousness. PECARN includes loss of consciousness for over 5 seconds in the under 2 years of age cohort and loss of consciousness of an unspecified duration for the over 2 cohort. Differing to PECARN, CHALICE uses a duration of over 5 minutes of loss of consciousness in any child under 16 years of age, and HIDATq does not specify a duration of loss of consciousness.
**Vomiting**	This clinical criterion is a predictor in 12/25 tools, with four varying descriptors including 'any post injury vomiting', 'over 1 vomit', 'over 2 vomits' or 'over 3 vomits'. PECARN includes 'any vomiting' for the over 2-year-old cohort, as does the HIDATq tool, whereas CHALICE takes a different, less risk averse approach including '3 or more' [13]. Both CHALICE and PECARN have a high sensitivity and acceptable specificity for recognising clinically important traumatic brain injury, suggesting that both tools are appropriate for assessing mild head injury in children in

For peer review only

Supplementary Table 4. Narrative synthesis of clinical criteria:

	the ED, despite the varying descriptions of vomiting. NEXUS 2 includes 'over 1 vomit' and CATCH stipulates 'over 2 vomits', but as eligibility criterion
**Neurological deficit**	For the purposes of this narrative description, neurological deficit will also include focal neurology, Glasgow Coma Scale score (GCS), altered mental status, behavioural change, drowsiness, and vertigo.<br>The research evidence that underpinned the DA DALT tool concluded that neurological deficit is one of the predictors that significantly increases the likelihood of intracranial injury [33]. Similarly, the CIDSS2 score concluded that neurological deficit was amongst the most important predictors for identifying whether paediatric patients should be admitted to hospital with a head injury [26]. There are varying descriptions to define a neurological deficit across the 25 tools, and there is some cross over with altered mental status/behavioural change, drowsiness, and GCS. Some terms include vision changes, gait changes, repetitive questioning/speech, delayed response to external stimuli, sensory deficit, disorientation or just stating 'neurological deficit'. Authors of the SNC guidelines suggest that the clinical criteria 'altered mental status' is too complicated to use effectively, with potential to lead to unacceptable increases in CT rates [29]. Both PECARN (under 2) and HIDATq include 'patient well with no clinical concern/acting normal as per parent', which suggests no neurological deficit.<br>Many tools opt to use 'abnormal mental status' with varying descriptors, such as confusion, somnolence, repetitive or slow to respond to verbal communication. A GCS under 15 is associated with the presence of intracranial acute pathologies in young children with minor blunt head trauma [41]. GCS is a clinical criterion in 15 of the 25 identified tools, including PECARN, CHALICE and CATCH.<br>KLEMETTI is one of the only tools to include 'vertigo' as a clinical criterion; the authors found this variable was amongst one of the most significant to predict severely complicated head trauma in their multivariate logistic regression analysis [20]. ATABAKI is the only other tool to include vertigo as a clinical criterion, however both these tools have low specificity and were not evaluated in a published validation study after development.
**Headache**	Headache is a clinical predictor in only half of the 25 in-hospital tools. This may be because it is challenging to assess for a headache in younger children; they may express pain differently with signs such as irritability and persistent crying [42]. The authors of the BUCHANIC tool use headache as a clinical predictor, despite an inclusion criterion of aged under 3 years only, however, this tool is not validated. PECARN includes headache for the aged 2- 18 years cohort, but not the aged under 2 years. For children who can verbalise or show that they have a headache, it is important to consider severity and timing of the headache. Some tools specify 'severe headache', 'prolonged headache' or 'worsening headache'. Worsening headache is associated with intracranial injury, being deemed a 'high risk' criterion in CATCH [14]. The HIDATq tool does not include headache as a clinical predictor, despite presenting the tool as 'ultra-safe', although it does include 'irritability' which could indicate pain in a younger child.
**Seizure**	Just under half of the 25 in-hospital tools included seizure as a clinical predictor despite a post traumatic seizure being a high determinant for an intracranial injury [43]. The CIDSS2 score found seizure to be one of the most significant criteria to identify whether patients should be admitted with head injury. PECARN is the most widely validated and implemented tool, however it does not include seizure as a specific clinical predictor, compared with CHALICE and HIDATq, which do.
**Severe mechanism of injury**	Nine of the 25 identified clinical decision support tools include 'severe or dangerous mechanism of injury' as a clinical predictor. The description of what constitutes a 'severe' mechanism of injury varies across the tools, from different levels of fall from height, road traffic collisions, to specific considerations such as a bicyclist not wearing a helmet who is struck by a car, however; fall from height is the most common mechanism of injury in children. The SNC guideline [29] does not include high-energy trauma mechanism as a strict risk factor,

For peer review only

Supplementary Table 4. Narrative synthesis of clinical criteria:

	as these patients are relatively uncommon in Scandinavia and are managed according to separate clinical trauma protocols. The authors and creators of the SNC guidelines judged 'mechanism of injury' as a complicated clinical predictor to use, due to having a specific definition and often including assessment of fall height, vehicle speed and number of stairs [29]. Similarly, the Novel Simplified Tool [31] suggests that mechanism of injury is not considered reliable, and that it may be an overestimation to emphasise the injury mechanism as a predictor. The aim of this clinical decision tool is to provide a simplified version of PECARN, CATCH and CHALICE (all of which include varying descriptors for mechanism of injury), so that it can be easily and quickly used for paediatric patients in busy emergency settings. Therefore, one distinguishing feature is that that this tool includes only one predictor for mechanism of injury, however; the Novel CDR misclassified eight patients as low risk.
**Amnesia**	The authors of PALCHAK conclude that elimination of amnesia as a clinical predictor may decrease unnecessary CT scanning [16]. However, amnesia is a clinical predictor in eight of the 25 hospital-based tools identified within this review. It is not possible to assess amnesia in non-verbal children and it is difficult to assess in young children [33] which is perhaps why PECARN does not include amnesia as a clinical predictor, however all of CHALICE, CATCH, NEXUS 2 and HIDATq do. One study adapted the PECARN tool by introducing amnesia alongside other clinical predictors [44]. The authors concluded that the adapted PECARN rule was successfully implemented in an Italian Paediatric ED; its use determined a low CT scan rate that was unchanged compared to previous clinical practice and showed an optimal safety and high efficacy profile.
**Non-accidental injury**	Non-accidental injury is only included as a clinical predictor in two of the in-hospital clinical decision support tools (HIDATq and CHALICE), although some tools include non-accidental injury as an exclusion criterion, such as the Head Injury EBG algorithm and CATCH. Despite this, non-accidental injury is one of the biggest predictors of brain injury, particularly in children under the age of 2 years [45]. PECARN is not especially sensitive for identifying physically abused children [46]. There is no gold standard diagnostic test to identify abusive head trauma though PredAHT and PediBIRN are specifically designed tools that can help clinicians to recognise cases of abusive head trauma [46].
**Clotting impairment**	Only three of the 25 in-hospital tools include clotting/impairment as a clinical predictor (HIDATq, the SNC guideline and NEXUS 2), since it is uncommon for a child to be on these types of drugs or have bleeding diatheses. The HIDATq tool is derived using the NICE head injury guidance and is designed to be 'ultra-safe' since the aim of the tool is to discharge children with head injury at triage, and therefore includes 'anticoagulant use/clotting impairment. None of the three most widely validated tools (PECARN, CATCH and CHALICE) include 'anticoagulant use/clotting disorder' as a clinical predictor, because it was too rare to be of use.
**Drug and alcohol use**	It is well known that alcohol increases the risk of suffering a head injury due to its effects on balance and coordination [47]. Children with suspected drug or alcohol intoxication may be more difficult to assess; clinicians should assume conscious level and amnesia relates to injury and have a lower threshold for referral and neuroimaging, since blunt head injuries in intoxicated patients is associated with a higher mortality [48]. Alcohol can also lead to headaches, nausea, and irritability, making examination challenging. Five of the identified clinical decision support tools included 'drug/alcohol use' (HIDATq, HAYDEL, ATABAKI, CIDSS2, BIG-1). None of the most validated tools, PECARN, CHALICE, or CATCH include this as a clinical predictor.
**Age**	All the identified clinical decision support tools in this review specify an age in which the tool can be used, e.g., under 18 years, and this varies from aged under 2-years to under 21-year-olds. However, only two tools exclude younger age groups as a clinical predictor (HIDATq excludes under 1s and ATABAKI excludes under 2's). HAYDEL is only for five–17-year-olds and PECARN has a separate tool for under 2's, with different questions, more specific to that age group [6]. BUCHANIC, PredAHT and PediBIRN are designed specifically for under 3's, and

For peer review only

Supplementary Table 4. Narrative synthesis of clinical criteria:

	GREENES is for under 2's only, however despite these tools aiming at the same age group, their clinical predictors still vary considerably. The authors of GREENES found that infants are at greater risk of intracranial hypertension due to their open fontanels and that under 1's with intracranial injuries are frequently asymptomatic, making management inherently difficult for clinicians [19]. It may be for this reason (as well as the increased likelihood of non-accidental injury) that children under 3 months were significantly more likely to receive a CT scan using the PECARN tool.

For peer review only

KIN 4400 Independent Research Study in Kinesiology | PRISMA 2020 Checklist

Section and Topic	Item #	Checklist item	Location where item is reported
**TITLE**			
Title	1	Identify the report as a literature review.	Page 1
**ABSTRACT**			
Abstract	2	Provide a structured summary including, as applicable: background; objectives; data sources; study eligibility criteria, participants, and interventions; study appraisal and synthesis methods; results; limitations; conclusions and implications of key findings.<br>See the PRISMA 2020 for Abstracts checklist for the complete list.	1 and 2
**INTRODUCTION**			
Rationale	3	Describe the rationale for the review in the context of existing knowledge, i.e., what is already known about your topic.	2 and 3
Objectives	4	Provide an explicit statement of the objective(s) or question(s) the review addresses with reference to participants, interventions, comparisons, outcomes, and study design (PICOS).	3
**METHODS**			
Eligibility criteria	5	Specify the inclusion and exclusion criteria for the review and how studies were grouped for the syntheses with study characteristics (e.g., PICOS, length of follow-up) and report characteristics (e.g., years considered, language, publication status) used as criteria for eligibility, giving rationale.	4
Information sources	6	Specify all databases, registers, websites, organisations, reference lists and other sources searched or consulted to identify studies. Specify the date when each source was last searched or consulted.	3
Search strategy	7	Present the full search strategies for all databases, registers and websites, including any filters and limits used.	3,4,5,6
Selection process	8	State the process for selecting studies (i.e., screening, eligibility).<br>Specify the methods used to decide whether a study met the inclusion criteria of the review, including how many reviewers screened each record and each report retrieved, whether they worked independently, and if applicable, details of automation tools used in the process.	3,4,5,6
Study risk of bias assessment	11	Specify the methods used to assess risk of bias in the included studies, including details of the tool(s) used, how many reviewers assessed each study and whether they worked independently, and if applicable, details of automation tools used in the process.	3,4,5
**RESULTS**			
Study selection	16a	Describe the results of the search and selection process, from the number of records identified in the search to the number of studies included in the review, ideally using a flow diagram.	6

The checklist has been adapted for KIN 4400 Independent Research Study in Kinesiology at the University of Guelph-Humber. Last updated: Dec 9, 2021

Adapted From: Page MJ, McKenzie JE, Bossuyt PM, Boutron I, Hoffmann TC, Mulrow CD, et al. The PRISMA 2020 statement: an updated guideline for reporting systematic reviews. BMJ 2021;372:n71. doi: 10.1136/bmj.n71 *Adapted From:* Page MJ, McKenzie JE, Bossuyt PM, Boutron I, Hoffmann TC, Mulrow CD, et al. The PRISMA 2020 statement: an updated guideline for reporting systematic reviews. BMJ 2021;372:n71. doi: 10.1136/bmj.n71

For more information, visit: http://www.prisma-statement.org/

For peer review only - http://bmjopen.bmj.com/site/about/guidelines.xhtml

KIN 4400 Independent Research Study in Kinesiology | PRISMA 2020 Checklist

Section and Topic	Item #	Checklist item	Location where item is reported
	16b	Cite studies that might appear to meet the inclusion criteria, but which were excluded, and explain why they were excluded.	6
Study characteristics	17	Cite each included study and present its characteristics (e.g., study size, PICOS, follow-up period).	6
Risk of bias in studies	18	Present assessments of risk of bias for each included study.	N/A
Results of individual studies	19	For all outcomes, present, for each study: (a) summary statistics for each group (where appropriate) and (b) an effect estimate and its precision (e.g. confidence/credible interval), ideally using structured tables or plots.	6,7,8,9,13,14,15,16
**DISCUSSION**			
Discussion	23a	Provide a general interpretation of the results in the context of other evidence.	20
	23b	Discuss any limitations of the evidence included in the review.	23
	23c	Discuss any limitations of the review processes used.	23
	23d	Discuss implications of the results for practice, policy, and future research.	23
**OTHER INFORMATION**			
Registration and protocol	24a	Provide registration information for the review, including register name and registration number, or state that the review was not registered.	3
	24b	Indicate where the review protocol can be accessed, or state that a protocol was not prepared.	3
	24c	Describe and explain any amendments to information provided at registration or in the protocol.	N/A
Support	25	Describe sources of financial or non-financial support for the review, and the role of the funders or sponsors in the review.	28
Competing interests	26	Declare any competing interests of review authors.	27
Availability of data, code, and other materials	27	Report which of the following are publicly available and where they can be found: template data collection forms; data extracted from included studies; data used for all analyses; analytic code; any other materials used in the review.	n/a

The checklist has been adapted for KIN 4400 Independent Research Study in Kinesiology at the University of Guelph-Humber. Last updated: Dec 9, 2021

Adapted From: Page MJ, McKenzie JE, Bossuyt PM, Boutron I, Hoffmann TC, Mulrow CD, et al. The PRISMA 2020 statement: an updated guideline for reporting systematic reviews. BMJ 2021;372:n71. doi: 10.1136/bmj.n71 *Adapted From:* Page MJ, McKenzie JE, Bossuyt PM, Boutron I, Hoffmann TC, Mulrow CD, et al. The PRISMA 2020 statement: an updated guideline for reporting systematic reviews. BMJ 2021;372:n71. doi: 10.1136/bmj.n71

For more information, visit: http://www.prisma-statement.org/

For peer review only - http://bmjopen.bmj.com/site/about/guidelines.xhtml

BMJ Open

Which elements of hospital-based clinical decision support tools for the assessment and management of children with head injury can be adapted for use by paramedics in pre-hospital care? A systematic mapping review and narrative synthesis.

Journal:	*BMJ Open*
Manuscript ID	bmjopen-2023-078363.R2
Article Type:	Original research
Date Submitted by the Author:	13-Dec-2023

Complete List of Authors:	Proctor, Alyesha; University of the West of England,<br>Lyttle, Mark; University of the West of England Bristol, Faculty of Health and Applied Sciences<br>Billing, Jedd; University of the West of England<br>Shaw, Pauline; University of the West of England<br>Simpson, Julian; University of the West of England<br>Voss, Sarah; University of the West of England, Health and Life Sciences<br>Benger, Jonathan; The University Hospitals NHS Foundation trust, Academic Department of Emergency care; The University of the West of England, Faculty of Health & Life Sciences

**Primary Subject Heading**:	Emergency medicine
Secondary Subject Heading:	Paediatrics
Keywords:	Systematic Review, PAEDIATRICS, ACCIDENT & EMERGENCY MEDICINE

SCHOLARONE™
Manuscripts

I, the Submitting Author has the right to grant and does grant on behalf of all authors of the Work (as defined in the below author licence), an exclusive licence and/or a non-exclusive licence for contributions from authors who are: i) UK Crown employees; ii) where BMJ has agreed a CC-BY licence shall apply, and/or iii) in accordance with the terms applicable for US Federal Government officers or employees acting as part of their official duties; on a worldwide, perpetual, irrevocable, royalty-free basis to BMJ Publishing Group Ltd ("BMJ") its licensees and where the relevant Journal is co-owned by BMJ to the co-owners of the Journal, to publish the Work in this journal and any other BMJ products and to exploit all rights, as set out in our licence.

The Submitting Author accepts and understands that any supply made under these terms is made by BMJ to the Submitting Author unless you are acting as an employee on behalf of your employer or a postgraduate student of an affiliated institution which is paying any applicable article publishing charge ("APC") for Open Access articles. Where the Submitting Author wishes to make the Work available on an Open Access basis (and intends to pay the relevant APC), the terms of reuse of such Open Access shall be governed by a Creative Commons licence – details of these licences and which Creative Commons licence will apply to this Work are set out in our licence referred to above.

Other than as permitted in any relevant BMJ Author's Self Archiving Policies, I confirm this Work has not been accepted for publication elsewhere, is not being considered for publication elsewhere and does not duplicate material already published. I confirm all authors consent to publication of this Work and authorise the granting of this licence.

For peer review only

TITLE:

Which elements of hospital-based clinical decision support tools for the assessment and management of children with head injury can be adapted for use by paramedics in pre-hospital care? A systematic mapping review and narrative synthesis.

AUTHORS:

Corresponding author: Alyesha Proctor[1]

Dr Mark D Lyttle[1]

Jedd Billing[1]

Pauline Shaw[1]

Julian Simpson[1]

Professor Sarah Voss[1]

Professor Jonathan Benger[1]

[1]Faculty of Health and Applied Sciences, University of the West of England, Glenside Campus (1H30), Blackberry Hill, Bristol, BS16 1DD, England

Alyesha.proctor@uwe.ac.uk (NIHR Clinical Doctoral Research Fellow, Advanced Paramedic Practitioner)

Mark.Lyttle@uwe.ac.uk (Senior Research Fellow, Consultant in Paediatric Emergency Medicine)

Jedd.billing@uwe.ac.uk (Senior Lecturer in Paramedic Science)

Pauline.shaw@uwe.ac.uk (Subject Librarian)

Julian.simpson@uwe.ac.uk (Programme Lead, Senior Lecturer in Paramedic Science)

Sarah.voss@uwe.ac.uk (Professor of Emergency Care)

Jonathan.benger@uwe.ac.uk (Professor of Emergency Care, Consultant in Emergency Medicine)

*Corresponding author address: Alyesha Proctor, Glenside Campus, Blackberry Hill, Stapleton, Bristol, BS16 1DD.

ABSTRACT:

Objective:

Hospital-based clinical decision tools support clinician decision making when a child presents to the Emergency Department with a head injury, particularly regarding computed tomography scanning. However, there is no decision tool to support pre-hospital clinicians in deciding which head injured children can safely remain at scene. This study aims to identify clinical decision tools, or constituent elements, which may be adapted for use in pre-hospital care.

Design:

Systematic mapping review and narrative synthesis.

Data sources:

For peer review only - http://bmjopen.bmj.com/site/about/guidelines.xhtml

Searches were conducted using MEDLINE, EMBASE, PsycINFO, CINAHL and AMED.

Eligibility criteria:

Quantitative, qualitative, mixed methods or systematic review research that included a clinical decision support tool for assessing and managing children with head injury.

Data extraction and synthesis:
We systematically identified all in-hospital clinical decision support tools and extracted from these the clinical criteria used in decision making. We complemented this with a narrative synthesis.

Results:
Following de-duplication 887 articles were identified. After screening titles and abstracts, 710 articles were excluded, leaving 177 full text articles. Of these, 95 were excluded, yielding 82 studies. A further 14 studies were identified in the literature after cross checking, totalling 96 analysed studies. 25 relevant in-hospital clinical decision tools were identified, encompassing 67 different clinical criteria, which grouped into 18 categories.

Conclusion:
Factors that should be considered for use in a clinical decision tool designed to support paramedics in the assessment and management of children with head injury, are: signs of skull fracture; a large, boggy or non-frontal scalp haematoma neurological deficit; Glasgow Coma Scale score less than 15; prolonged or worsening headache; prolonged loss of consciousness; post traumatic seizure; amnesia in older children; non-accidental injury; drug or alcohol use; less than one year old. Clinical criteria that require further investigation include mechanism of injury; clotting impairment/anticoagulation; vertigo; length of time of unconsciousness; number of vomits.

Strengths and limitations of the study:

- The review is highly inclusive, with a range of global study settings, including qualitative, quantitative, and mixed methods research.
- The review provided an opportunity to complete a detailed and contemporary search of all available sources and identified 25 in-hospital tools, compared to 14 in a previous systematic review.
- The role of mapping reviews is to provide a descriptive account of the published literature; therefore, this should be considered when assessing the findings of the overall evidence synthesis.
- A direct comparison of the clinical decision support tools is not possible because they address different questions, age groups, injury severities, and outcomes.

INTRODUCTION:

Healthcare systems internationally are under substantial pressure due to rising patient demand. A leading priority is to reduce the number of patients who are unnecessarily conveyed to an Emergency Department (ED) by ambulance [1]. Children frequently use the ambulance service and ED, and childhood head injury is common; approximately 800,000 head-injured children attend EDs each year in the UK [2]. Very few children with head injury who are conveyed to the ED by ambulance need specialist treatment, and the vast majority could be managed safely at scene.

For peer review only - http://bmjopen.bmj.com/site/about/guidelines.xhtml

Despite this, a national overview of head injury in children found that one-third of children in hospital with head injury were transported by ambulance, and 74% of those conveyed were non-serious, requiring no intervention [3].

Head injury guidance produced by the National Institute for Health and Care Excellence (NICE) [4] is of limited utility to paramedics for reasons including: a lack of validation in pre-hospital care; reference to patient observation over time which is not always possible; challenges in the assessment of amnesia in younger children; limited research evidence on which to base recommendations relating to ambulance non-conveyance [4].

There are several existing validated hospital-based decision support tools that clinicians use when a child presents to the ED with a minor head injury, and studies have demonstrated their effectiveness in aiding decision-making for both immediate discharge and computed tomography (CT) scanning [5] [6]. Currently, there are no pre-hospital decision tools designed to support paramedics in reducing unnecessary conveyance to the ED for head-injured children. None of the existing hospital-based tools can be implemented directly in pre-hospital care, since they are designed to support decision making on performing CT scanning, rather than ED conveyance.

AIM:

To systematically identify and examine clinical criteria within existing hospital-based clinical decision support tools for children with minor head injury, with a focus on determining their potential to aid decision making regarding hospital conveyance by paramedics.

METHODS:

PATIENT AND PUBLIC INVOLVEMENT:

Patients and public have been involved throughout this research. Both a Young Persons Advisory Group (YPAG) (children aged 9-16 years) and Parent Advisory Group (PAG) have supported this study and confirmed its importance for children, parents, and the wider health and care system.

Introduction:

Systematic mapping aims to collate, explain, and catalogue available evidence (primary and secondary) relating to a topic of interest, to identify a focus for more specific investigation [7]. It is a review methodology that is often used in healthcare research that aims to detail and categorise literature on a particular topic, with the intention of developing this into more comprehensive work [8]. This systematic mapping review was conducted in accordance with published methods [9] and is reported in line with the PRISMA statement [10]. It has not been registered with Prospero as it is a mapping review.

Patients and public have been involved throughout this research. Both a Young Persons Advisory Group (YPAG) (children aged 9-16 years) and Parent Advisory Group (PAG) have supported this study and confirmed its importance for children, parents, and the wider health and care system.

Search strategy:

Searches were conducted in the following databases, for articles published between January 1980 and August 2022 to ensure that all relevant contemporary research was included: MEDLINE, EMBASE, PsycINFO, CINAHL and AMED **[supplementary material search strategy].** A Google Scholar and a Web of Science search were undertaken to identify reports or proceedings not indexed in the

For peer review only - http://bmjopen.bmj.com/site/about/guidelines.xhtml

above. A literature advisory group, including experts in the field, was consulted to identify relevant grey literature and unpublished reports. Furthermore, to find additional published, unpublished, and ongoing studies, the reference lists of all relevant studies were examined.

Search terms were developed iteratively by discussion among the research team, patient and public representatives and a librarian, seeking a balance between comprehensiveness and focus. A combination of MeSH terms and synonym text-strings/phrases were used in the search strategy and were combined using Boolean operators. Updated searches were re-run before the final analysis. The search terms used can be seen in Table 1.

The search was limited to English language studies due to resource restrictions and the cost of translation, and only articles with full texts available were included. Studies that may have been relevant but did not include or refer to a clinical decision support tool for the assessment and management of head-injured children, were excluded; however, there were no restrictions on the types of study included. Eligibility criteria are presented in full in Table 2.

Table 1. Search terms:

Clinical decision support tool	Head injury	Children	Hospital
Clinical decision tool	Minor head injury	Child	ED
Clinical decision rule	TBI (Traumatic brain injury)	Pediatric	Emergency Department
Diagnostic accuracy tool	Head trauma	Paediatric	A+E
Triage tool	Head wound	Baby	Accident and Emergency
Hospital-based tool	Intracranial injury	Babies	Emergency room
CDRs	CiTBI (clinical important traumatic brain injury)	Infant	Trauma centre
Intervention	ICI (intracranial injury)	Schoolchild	
Clinical decision score	CSII (clinically significant intracranial injury)	Adolescent	
Paediatric head injury predictive tool	Trivial head injury	Teenager	
Clinical Prediction Rule		Young person	
Clinical Prediction Tool			
Clinical decision support tool (CDST)			

Table 2. Inclusion and exclusion criteria:

Inclusion Criteria	Exclusion Criteria
Studies in the English Language	Non- English language
Quantitative, qualitative, mixed methods research, systematic reviews	
Children (under 19 years of age)	Adult population (19 years or older)

For peer review only - http://bmjopen.bmj.com/site/about/guidelines.xhtml

For peer review only

Date of publication 1980-present	
Any study that includes a clinical decision support tool for assessing and managing children with head injury	Any study that did not include a clinical decision support tool for the assessment and management of head-injured children
International as long as in the English language	

Extracting, Coding, and Synthesising the Data:

Titles and abstracts were double screened for relevance and assessed for eligibility using Covidence software [11], following which full texts of included articles underwent review for eligibility. Any disagreements were resolved through discussion with a senior researcher. Reasons for exclusion were documented, a topic expert reviewed the final set of included papers to ensure relevant studies and tools had not been overlooked. Figure 1 shows the PRISMA flow diagram [10].

Data extraction included systematically identifying the title of each tool, the aim, derivation reference, supporting literature, conclusion, and key points relating to the content and format of a clinical decision support tool for paramedic use. An inductive coding frame was developed to map emerging concepts, which involved deriving codes from the data. Key messages/concepts in relation to predicting conveyance from all studies were extracted from the methods, results, and conclusion sections. This resulted in a visual synthesis and classification of available studies and tools. The data extraction tool was piloted using two included studies, and data were extracted by hand and cross-checked by another researcher.

Clinical criteria used in decision making were extracted from all tools; a summary map was produced providing a visual display of clinical criteria included in each tool. After independently producing pilot categories based on a sample of clinical decision support tools, the research team formed consensus on categories. The frequency of each clinical criterion was tabulated, and performance accuracy was recorded where reported. A narrative synthesis was subsequently developed using this framework.

Assessment of Quality:

No formal assessment of quality assessment was undertaken, as this is not required in mapping reviews [12]. Data extraction in mapping reviews describes the studies and their main methods but does not attempt a full data extraction and quality assessment [12].

RESULTS:

A total of 887 articles were identified after duplicates were removed. After screening titles and abstracts, 710 articles were excluded, which left 177 full text articles to be assessed for eligibility. Of these, 95 were excluded for reasons including: adult population only; not including a clinical decision support tool; not in the English language; and full text not available. Three articles were not included, as they were conference abstracts. Fourteen additional studies were identified through citation searching, resulting in 96 included studies (Figure 1).

[Figure 1. PRISMA flow diagram]

In total, 25 relevant in-hospital clinical decision support tools for the assessment and management of children with head injury were identified, which can be seen in Table 3.

Table 3. Hospital based clinical decision support tools for the assessment and management of children with head injury:

For peer review only - http://bmjopen.bmj.com/site/about/guidelines.xhtml

CDST	Full title	Reference
PECARN	Paediatric Emergency Care Applied Research Network rule.	[6] Kuppermann, N., *et al.* (2009) Identification of children at very low risk of clinically important brain injuries after head trauma: A prospective cohort study. *The Lancet,* 374(9696), pp. 1160-1170.
CHALICE	Children's Head injury Algorithm for the prediction of Important Clinical Events Rule.	[13] Dunning, J., Daly, J., Lomas, J., Lecky, F., Batchelor, J. and Mackway-Jones (2006). Derivation of the children's head injury algorithm for the prediction of important clinical events decision rule for head injury in children. *Archives of Disease in Childhood,* 91(11), pp. 885-91.
CATCH	Canadian Assessment of Tomography for Childhood Head injury Rule.	[14] Osmond, M.H. (2010) CATCH: a clinical decision rule for the use of computed tomography in children with minor head injury, *Canadian Medical Association Journal,* 182(4).
NEXUS II	National Emergency X-Ray Utilization Study.	[15] Oman, J. A., Cooper, R. J., Holmes, J. F., Viccellio, P., Nyce, A., Ross, S. E., ... & Mower, W. R. (2006). Predictive performance of a decision rule to predict need for computed tomography among children with blunt head trauma. *Pediatrics,* 117(2), pp. 238-246.
HIDATq	Head Injury Discharge At Triage questionnaire.	[5] Aldridge, P., Castle, H., Phillips, C., Russel, E., Guerrero-Luduena, R. and Rout, R. (2020). Head home: a prospective cohort study of a nurse led paediatric head injury clinical decision tool at a district general hospital, Emergency Medical Journal, 37(11), pp. 680-685.
PALCHAK	UC Davis Rule for Paediatric Head Trauma.	[16] Palchak, M.J *et al.* (2003). A decision rule for identifying children at low risk for brain injuries after blunt head trauma, *Annals of Emergency Medicine*, 42(4), pp. 492-506
HAYDEL	New Orleans/Charity Head Trauma/Injury Rule.	[17] Haydel, M.J. and Shembekar, A. D. (2003) Prediction of intracranial injury in children aged five years and older with loss of consciousness after minor head injury due to nontrivial mechanisms, *Annals of Emergency Medicine,* 42(4).
ATABAKI	No title	[18] Atabaki, S. M., Stiell, I. G., Bazarian, J. J., Sadow, K. E., Vu, T. T., Camarca, M. A., ... & Chamberlain, J. M. (2008). A clinical decision rule for cranial computed tomography in minor pediatric head trauma. *Archives of pediatrics and adolescent medicine,* 162(5), pp. 439-445
GREENES	No title	[19] Greenes, D. S., & Schutzman, S. A. (1999). Clinical indicators of intracranial injury in head-injured infants. *Pediatrics,* 104(4), pp. 861-867.
KLEMETTI	No title	[20] Klemetti, S., Uhari, M., Pokka, T., & Rantala, H. (2009). Evaluation of decision rules for identifying serious consequences of traumatic head injuries in pediatric patients. Pediatric emergency care, 25(12), 811-815
QUAYLE	No title	[21] Quayle, K. S., Jaffe, D. M., Kuppermann, N., Kaufman, B. A., Lee, B. C., Park, T. S., & McAlister, W. H. (1997). Diagnostic testing for acute head injury in children: when are head computed tomography and skull radiographs indicated? *Pediatrics,* 99(5).
DIETRICH	No title	[22] Dietrich, A. M., Bowman, M. J., Ginn-Pease, M. E., Kosnik, E., & King, D. R. (1993). Pediatric head injuries: can clinical factors reliably predict an abnormality on computed tomography? *Annals of emergency medicine, 22*(10), pp. 1535-1540.
GUZEL	No title	[23] Güzel, A., Hiçdönmez, T., Temizöz, O., Aksu, B., Aylanç, H., & Karasalihoglu, S. (2009). Indications for brain computed tomography and hospital admission in pediatric patients with minor head injury: how much can we rely upon clinical findings?. *Pediatric neurosurgery,* 45(4), pp. 262-270

For peer review only - http://bmjopen.bmj.com/site/about/guidelines.xhtml

PredAHT	Predicting Abusive Head Trauma	[24] Cowley, Laura E *et al.* (2018) Acceptability of the Predicting Abusive Head Trauma (PredAHT) clinical prediction tool: A qualitative study with child protection professionals, *Child Abuse & Neglect* 81, pp. 192-205.
CHIDA	Children's Intracranial Injury Decision Aid.	[25] Neumayer, Katie *et al.* (2020) Validation of the "CHIIDA" and application for PICU triage in children with complicated mild traumatic brain injury. *Journal of pediatric surgery*, 55(7), pp. 1255-1259
CIDSS2	Stands for:<br>Coma scale<br>Intoxication<br>Deficit (neurological)<br>Seizure<br>Skull fracture 2	[26] Greenberg, J., Yan, Y., Carpenter, C., Lumba-Brown, A., Keller, M., Pineda, J., Brownson, R. and Limbrick, D. (2018) Development of the $CIDSS_2$ Score for Children with Mild Head Trauma without Intracranial Injury. *Journal of Neurotrauma,* 35(22), pp. 2699-2707.
PediBIRN	Paediatric Brain Injury Research Network	[27] Pfeiffer, H., Smith, A., Kemp, M., Cowley, E., Cheek, A., Dalziel, S., Borland, S., O'Brien, S., Bonisch, M., Neutze, J., Oakley, E., Crowe, L., Hearps, S., Lyttle, M., Bressan, S. and Babl, F. (2018) External validation of the PediBIRN clinical prediction rule for abusive head trauma. *Paediatrics,* 141(5).
Head CT Choice	Head Computed Tomography Choice	[28] Hess, Erik *et al.* (2014) Effectiveness of the head CT choice decision aid in parents of children with minor head trauma: study protocol for a multicentre randomized trial. *Trials,* 15(1), pp. 253-253
SNC Tool	The Scandinavian Neurotrauma Committee Guidance	[29] Unden, J. *et al.* (2018) External validation of the Scandinavian guidelines for management of minimal, mild and moderate head injuries in children, *BMC Medicine,* 16(176).
BIG-1	Brain Injury Guidelines	[30] Schwartz, Jamie *et al.* (2020)<br>Applying Pediatric Brain Injury Guidelines at a Level I Adult/Pediatric Safety-Net Trauma Center.<br>*The Journal of surgical research,* 255, pp. 106-110
Novel Simplified CDR	Novel Simplified Clinical Decision Rule	[31] Yogo, N., Toida, C., Muguruma, T., Gakumazawa, M., Shinohara, M. and Takeuchi, I. (2021) Simplified Clinical Decision Rule Using Clinically Important Events for Risk Prediction in Pediatric Head Injury: A Retrospective Cohort Study, *Journal of Clinical Medicine,* 10(22).
Head Trauma EBG Algorithm	Head Trauma EBG Algorithm	[32] Brittany M. Stopa*, et al.* (2019) Comparison of minor head trauma management in the emergency departments of a United States and Italian Children's hospital, *Italian Journal of Pediatrics,* 45(24).
Da Dalt	No title	[33] Da Dalt, L. *et al*. (2006) Predictors of intracranial injuries in children after blunt head trauma. *European Journal of Paediatrics,* 165(3), pp. 142–8.
Buchanich	No title	[34] Buchanich, J. M. (2007). A clinical decision-making rule for mild head injury in children less than three years old (Doctoral dissertation, University of Pittsburgh).
KIIDS-TBI-CDS	The Kids Intracranial Injury Decision Support tool for Traumatic Brain Injury	[35] Greenberg *et al*. (2021) Development and external validation of the KIIDS-TBI tool for managing children with mild traumatic brain injury and intracranial injuries. *Academic Emergency Medicine,* 28(12).

Sixty-seven decision-making criteria were extracted across all tools. Several criteria related to the same clinical feature (for example, vomiting), however there were many different cut points (e.g.,

For peer review only - http://bmjopen.bmj.com/site/about/guidelines.xhtml

vomiting once only, vomiting twice, or vomiting three times). These 67 criteria were grouped into 18 clinical categories: vomiting; headache; loss of consciousness (LOC); seizure; amnesia; dizziness/vertigo; clotting impairment/anticoagulated; non-accidental injury; age; severe mechanism of injury; drug/alcohol use; signs of a skull fracture; haematoma; neurological deficit; altered mental status; Glasgow come score (GCS); drowsiness and well with no clinical concern.

Supplementary Table 1 shows which clinical categories are included within each hospital-based tool.

[Supplementary Table 1. Categories in each tool]

For peer review only

For peer review only - http://bmjopen.bmj.com/site/about/guidelines.xhtml

Of these clinical categories, the most frequent were skull fracture, loss of consciousness, and scalp haematoma. The most infrequently used included vertigo, suspicion of non-accidental injury and patient acting normal as per parent. Table 4 tabulates the frequency of each clinical criterion.

[Table 4. Frequency of each clinical category across all the tools].

Categories	CDST	Frequency (n=25)
Signs of any description of skull fracture<br><br>(Depressed, basal skull, palpable)	PECARN 18, PECARN 2, NEXUS 2, HIDATq, PALCHACK, CHALICE, CATCH, GUZEL, BUCHANICH, DA DALT, KLEMETTI, QUAYLE, ATABAKI, SNC Guideline, BIG-1, Head trauma EBG algorithm, GREENES, CHIDA, CIDSS2, PediBIRN, KIIDS-TBI CDS	**21 (84%)**
LOC	PECARN 18, PECARN 2, CHALICE, CATCH, NEXUS 2, ATABAKI, KLEMETTI, QUAYLE, HIDATq, GUZEL, DIETRICH, DA DALT, BUCHANICH, SNC Guideline, BIG-1, Novel simplified CDR, Head trauma EBG algorithm	**17 (68%)**
Scalp haematoma/trauma	PECARN 2, CHALICE, PALCHAK, KLEMETTI, NEXUS 2, GUZEL, HAYDEL, GREENES, CATCH, HIDATq, BUCHANICH, ATABAKI, SNC Guideline, Head trauma EBG algorithm, PredAHT, PediBIRN, KIIDS-TBI CDS	**17 (68%)**
Neurological deficit	CHALICE, NEXUS 2, HIDATq, GUZEL, DIETRICH, DA DALT, BUCHANICH, KLEMETTI, ATABAKI, QUAYLE, Head CT Choice, SNC Guidelines, BIG-1, Head trauma EBG algorithm, CIDSS2	**15 (60%)**
Behaviour change/ altered mental status	PECARN 18, PECARN 2, CATCH, HIDATq, PALCHAK, ATABAKI, BUCHANICH, DA DALT, KLEMETI, QUAYLE, NEXUS 2, Head CT Choice, SNC Guidelines, Novel simplified CDR, Head trauma EBG algorithm	**15 (60%)**
GCS	PECARN 18, PECARN 2, CHALICE, NEXUS 2, CATCH, HIDATq, ATABAKI, CHIDA, CIDSS2, Head CT Choice, SNC	**15 (60%)**

For peer review only - http://bmjopen.bmj.com/site/about/guidelines.xhtml

For peer review only

	Guideline, Novel simplified CDR, Head trauma EBG algorithm, DA DALT, KIIDS-TBI CDT	
Vomiting	CHALICE, NEXUS 2, PECARN 18, PALCHACK, HIDATq, HAYDEL, DIETRICH, BUCHANICH, DA DALT, Head CT Choice, SNC Guidelines, Head trauma EBG algorithm	**12 (48%)**
Headache	PECARN 18, CATCH, PALCHAK, GUZEL, DIETRICH, HAYDEL, ATABAKI, BUCHANICH, DA DALT, Head CT Choice, SNC Guidelines, Novel simplified CDR	**12 (48%)**
Seizure	CHALICE, HIDATq, HAYDEL, ATABAKI, QUAYLE, DIETRICH, GUZEL, PredAHT, CIDSS2, SNC Guideline, Novel simplified CDR, DA DALT.	**12 (48%)**
Severe mechanism of injury	PECARN 18, PECARN 2, GUZEL CHALICE, CATCH, HIDATq, ATABAKI, Novel simplified CDR, Head trauma EBG algorithm	**9 (36%)**
Drowsiness	CHALICE, HIDATq, DA DALT, PECARN 18, PECARN 2, NEXUS 2, ATABAKI, Head CT Choice	**8 (32%)**
Amnesia	CHALICE, CATCH, NEXUS 2, HIDATq, HAYDEL, ATABAKI, GUZEL, DA DALT	**8 (32%)**
Drug or alcohol use	HIDATq, HAYDEL, ATABAKI, CIDSS2, BIG-1	**5 (20%)**
Age	PECARN, HIDATq, ATABAKI, HAYDEL	**4 (16%)**
Clotting impairment/anticoagulated	NEXUS 2, HIDATq, SNC Guideline	**3 (12%)**
Dizziness/vertigo	ATABAKI, KLEMETI	**2 (8%)**
Non accidental injury	CHALICE, HIDATq,	**2 (8%)**
Patient alert and well with no clinical concern/acting normal as per parent	PECARN 2, HIDATq	**2 (8%)**

The mix of clinical criteria used, and their outcomes were similar but not identical for any of the tools. In addition, the derivation studies used different populations and sample sizes and therefore the performance accuracy of the tools relating to the potential to correctly rule out clinically significant brain injury in head-injured children were variable *(*Supplementary Table 2).

For peer review only - http://bmjopen.bmj.com/site/about/guidelines.xhtml

[Supplementary Table 2. Performance accuracy of each tool].

Most tools displayed high sensitivities (>90%), while specificities ranged from 15 to 87%. PECARN was the most widely externally validated, with positive conclusions drawn from research examining post-implementation of the tool [36]. However, PECARN results in a considerable number of normal scans being performed to identify one intracranial injury [37]. The HIDATq tool was designed for nurses to use in triage to safely discharge children with head injury before admission, which more closely resembles the aim of a pre-hospital tool designed to inform which children do not need to be conveyed to hospital. A narrative synthesis is presented below.

Signs of skull fracture:

This was the most frequently used clinical decision-making criterion (21/25) (84%). Variability of terms included: open or depressed skull fracture, basal skull fracture, tense fontanelle, palpable and unclear skull fracture. The only tools that do not include signs of skull fracture are HAYDEL, DIETRICH, PredAHT and Novel Simplified CDR. All three of PECARN, CHALICE and CATCH (the three most widely validated tools) included signs of skull fracture. PredAHT does not include signs of skull fracture despite the aim of this tool being to identify abusive head trauma. In contrast, PediBIRN which aims to identify children with abusive head trauma does include signs of skull fracture as a clinical predictor [38]. The KIIDS-TBI CDS Tool concluded that a depressed skull fracture was a major risk factor for significant brain injury [35]. Babl, et al. (2014) [39] and Da Dalt et al. (2006) [33] found that a basal, open or depressed skull fracture was most likely to trigger a CT response out of any other clinical criteria. Similarly, Quayle et al. (1997) [21] concluded that the relative risk for intracranial injury is increased almost fourfold in the presence of a skull fracture, although the absence of skull fracture does not rule out intracranial injury. The CIDSS2 score [26], a tool to help physicians to identify the minority of children with minor traumatic brain injury at increased risk for extended inpatient stay, uses a scoring system; all clinical criteria score 1, apart from signs of skull fracture which scores 2, suggesting it is deemed the most significant clinical predictor of significant brain injury. Many of the tools, such as NEXUS 2, describe signs and symptoms of a skull fracture, rather than explicitly stating 'signs of a skull fracture'. For example: periorbital or periauricular ecchymoses, haemotympanum, and clear fluid from ears or nose [40].

Scalp haematoma:

Scalp haematoma is used in 17 of the tools identified. However, many specify 'non-frontal' haematomas only, such as the NEXUS 2 tool [40]. Others (GREENES) support inclusion of non-frontal haematomas only; the authors found that non-frontal scalp haematomas had the strongest association with presence of intracranial haemorrhage [19]. The authors of PALCHAK concluded a scalp haematoma is more significant in children under 2 years [16]. The HIDATq tool does not stipulate location of haematoma, however the authors do specify a size (over 5cm) [5]. Similarly, CHALICE only specifies size, which is also over 5cm, but this is for under 1-year olds only [13]. PECARN clearly specifies 'temporal, occipital or parietal haematoma', and this is for the under 2-year-old tool [6]. The CATCH tool includes 'large boggy scalp haematoma', however it is unclear what constitutes 'large' as it does not specify what size or the location. Most excluded haematomas to the face.

Loss of consciousness:

Loss of consciousness (LOC) has different cut points across the identified clinical decision tools, including 'any loss of consciousness', 'loss of consciousness for over 5 seconds' and 'loss of consciousness for over 5 minutes'. It is the second most frequently used clinical predictor, appearing

For peer review only - http://bmjopen.bmj.com/site/about/guidelines.xhtml

in 17 of the 25 clinical decision tools. The authors of PALCHAK [16] found that the risk of a traumatic brain injury was higher in those with a history of loss of consciousness. However, in those children with an isolated loss of consciousness there were no traumatic brain injuries. This is recognised in Kupperman et als. (2009) derivation study for PECARN [6]; the risk of clinically important traumatic brain injury was substantially lower than 1% in children with isolated loss of consciousness. PECARN includes loss of consciousness for over 5 seconds in the under 2 years of age cohort and loss of consciousness of an unspecified duration for the over 2 cohort. Differing to PECARN, CHALICE uses a duration of over 5 minutes of loss of consciousness in any child under 16 years of age, and HIDATq does not specify a duration of loss of consciousness.

Vomiting:

This clinical criterion is a predictor in 12/25 tools, with four varying descriptors including 'any post injury vomiting', 'over 1 vomit', 'over 2 vomits' or 'over 3 vomits'. PECARN includes 'any vomiting' for the over 2-year-old cohort, as does the HIDATq tool, whereas CHALICE takes a different, less risk averse approach including '3 or more' [13]. Both CHALICE and PECARN have a high sensitivity and acceptable specificity for recognising clinically important traumatic brain injury, suggesting that both tools are appropriate for assessing mild head injury in children in the ED, despite the varying descriptions of vomiting. NEXUS 2 includes 'over 1 vomit' and CATCH stipulates 'over 2 vomits', but as eligibility criterion.

Neurological deficit:

For the purposes of this narrative description, neurological deficit will also include focal neurology, Glasgow Coma Scale score (GCS), altered mental status, behavioural change, drowsiness, and vertigo.

The research evidence that underpinned the DA DALT tool concluded that neurological deficit is one of the predictors that significantly increases the likelihood of intracranial injury [33]. Similarly, the CIDSS2 score concluded that neurological deficit was amongst the most important predictors for identifying whether paediatric patients should be admitted to hospital with a head injury [26]. There are varying descriptions to define a neurological deficit across the 25 tools, and there is some cross over with altered mental status/behavioural change, drowsiness, and GCS. Some terms include vision changes, gait changes, repetitive questioning/speech, delayed response to external stimuli, sensory deficit, disorientation or just stating 'neurological deficit'. Authors of the SNC guidelines suggest that the clinical criteria 'altered mental status' is too complicated to use effectively, with potential to lead to unacceptable increases in CT rates [29]. Both PECARN (under 2) and HIDATq include 'patient well with no clinical concern/acting normal as per parent', which suggests no neurological deficit.

Many tools opt to use 'abnormal mental status' with varying descriptors, such as confusion, somnolence, repetitive or slow to respond to verbal communication. A GCS under 15 is associated with the presence of intracranial acute pathologies in young children with minor blunt head trauma [41]. GCS is a clinical criterion in 15 of the 25 identified tools, including PECARN, CHALICE and CATCH.

KLEMETTI is one of the only tools to include 'vertigo' as a clinical criterion; the authors found this variable was amongst one of the most significant to predict severely complicated head trauma in their multivariate logistic regression analysis [20]. ATABAKI is the only other tool to include vertigo as a clinical criterion, however both these tools have low specificity and were not evaluated in a published validation study after development.

For peer review only - http://bmjopen.bmj.com/site/about/guidelines.xhtml

Headache:

Headache is a clinical predictor in only half of the 25 in-hospital tools. This may be because it is challenging to assess for a headache in younger children; they may express pain differently with signs such as irritability and persistent crying [42]. The authors of the BUCHANIC tool use headache as a clinical predictor, despite an inclusion criterion of aged under 3 years only, however, this tool is not validated. PECARN includes headache for the aged 2- 18 years cohort, but not the aged under 2 years. For children who can verbalise or show that they have a headache, it is important to consider severity and timing of the headache. Some tools specify 'severe headache', 'prolonged headache' or 'worsening headache'. Worsening headache is associated with intracranial injury, being deemed a 'high risk' criterion in CATCH [14]. The HIDATq tool does not include headache as a clinical predictor, despite presenting the tool as 'ultra-safe', although it does include 'irritability' which could indicate pain in a younger child.

Seizure:

Just under half of the 25 in-hospital tools included seizure as a clinical predictor despite a post traumatic seizure being a high determinant for an intracranial injury [43]. The CIDSS2 score found seizure to be one of the most significant criteria to identify whether patients should be admitted with head injury. PECARN is the most widely validated and implemented tool, however it does not include seizure as a specific clinical predictor, compared with CHALICE and HIDATq, which do.

Severe mechanism of injury:

Nine of the 25 identified clinical decision support tools include 'severe or dangerous mechanism of injury' as a clinical predictor. The description of what constitutes a 'severe' mechanism of injury varies across the tools, from different levels of fall from height, road traffic collisions, to specific considerations such as a bicyclist not wearing a helmet who is struck by a car, however; fall from height is the most common mechanism of injury in children. The SNC guideline [29] does not include high-energy trauma mechanism as a strict risk factor, as these patients are relatively uncommon in Scandinavia and are managed according to separate clinical trauma protocols. The authors and creators of the SNC guidelines judged 'mechanism of injury' as a complicated clinical predictor to use, due to having a specific definition and often including assessment of fall height, vehicle speed and number of stairs [29]. Similarly, the Novel Simplified Tool [31] suggests that mechanism of injury is not considered reliable, and that it may be an overestimation to emphasise the injury mechanism as a predictor. The aim of this clinical decision tool is to provide a simplified version of PECARN, CATCH and CHALICE (all of which include varying descriptors for mechanism of injury), so that it can be easily and quickly used for paediatric patients in busy emergency settings. Therefore, one distinguishing feature is that that this tool includes only one predictor for mechanism of injury, however; the Novel CDR misclassified eight patients as low risk.

Amnesia:

The authors of PALCHAK conclude that elimination of amnesia as a clinical predictor may decrease unnecessary CT scanning [16]. However, amnesia is a clinical predictor in eight of the 25 hospital-based tools identified within this review. It is not possible to assess amnesia in non-verbal children and it is difficult to assess in young children [33] which is perhaps why PECARN does not include amnesia as a clinical predictor, however all of CHALICE, CATCH, NEXUS 2 and HIDATq do. One study adapted the PECARN tool by introducing amnesia alongside other clinical predictors [44]. The authors concluded that the adapted PECARN rule was successfully implemented in an Italian

For peer review only - http://bmjopen.bmj.com/site/about/guidelines.xhtml

Paediatric ED; its use determined a low CT scan rate that was unchanged compared to previous clinical practice and showed an optimal safety and high efficacy profile.

Non-accidental injury:

Non-accidental injury is only included as a clinical predictor in two of the in-hospital clinical decision support tools (HIDATq and CHALICE), although some tools include non-accidental injury as an exclusion criterion, such as the Head Injury EBG algorithm and CATCH. Despite this, non-accidental injury is one of the biggest predictors of brain injury, particularly in children under the age of 2 years [45]. PECARN is not especially sensitive for identifying physically abused children [46]. There is no gold standard diagnostic test to identify abusive head trauma though PredAHT and PediBIRN are specifically designed tools that can help clinicians to recognise cases of abusive head trauma [46].

Clotting impairment:

Only three of the 25 in-hospital tools include clotting/impairment as a clinical predictor (HIDATq, the SNC guideline and NEXUS 2), since it is uncommon for a child to be on these types of drugs or have bleeding diatheses. The HIDATq tool is derived using the NICE head injury guidance and is designed to be 'ultra-safe' since the aim of the tool is to discharge children with head injury at triage, and therefore includes 'anticoagulant use/clotting impairment. None of the three most widely validated tools (PECARN, CATCH and CHALICE) include 'anticoagulant use/clotting disorder' as a clinical predictor, because it was too rare to be of use.

Drug and alcohol use:

It is well known that alcohol increases the risk of suffering a head injury due to its effects on balance and coordination [47]. Children with suspected drug or alcohol intoxication may be more difficult to assess; clinicians should assume conscious level and amnesia relates to injury and have a lower threshold for referral and neuroimaging, since blunt head injuries in intoxicated patients is associated with a higher mortality [48]. Alcohol can also lead to headaches, nausea, and irritability, making examination challenging. Five of the identified clinical decision support tools included 'drug/alcohol use' (HIDATq, HAYDEL, ATABAKI, CIDSS2, BIG-1). None of the most validated tools, PECARN, CHALICE, or CATCH include this as a clinical predictor.

Age:

All the identified clinical decision support tools in this review specify an age in which the tool can be used, e.g., under 18 years, and this varies from aged under 2-years to under 21-year-olds. However, only two tools exclude younger age groups as a clinical predictor (HIDATq excludes under 1s and ATABAKI excludes under 2's). HAYDEL is only for five–17-year-olds and PECARN has a separate tool for under 2's, with different questions, more specific to that age group [6]. BUCHANIC, PredAHT and PediBIRN are designed specifically for under 3's, and GREENES is for under 2's only, however despite these tools aiming at the same age group, their clinical predictors still vary considerably. The authors of GREENES found that infants are at greater risk of intracranial hypertension due to their open fontanels and that under 1's with intracranial injuries are frequently asymptomatic, making management inherently difficult for clinicians [19]. It may be for this reason (as well as the increased likelihood of non-accidental injury) that children under 3 months were significantly more likely to receive a CT scan using the PECARN tool.

<u>DISCUSSION:</u>

For peer review only - http://bmjopen.bmj.com/site/about/guidelines.xhtml

This systematic mapping review identified and examined clinical criteria within existing hospital-based clinical decision support tools for children with minor head injury to determine their utility in paramedic practice, to inform the need for hospital conveyance. There are additional factors which require consideration in operationalising a tool for pre-hospital use, including the purpose of existing clinical decision rules, and additional relevant clinical factors.

Challenges of selecting a tool for use in pre-hospital care:

None of the existing hospital-based clinical decision support tools can be translated directly into paramedic practice. No single hospital based clinical decision support tool included all 18 clinical criteria, and there are potentially additional criteria that are not included in any of the tools that may be useful specifically in pre-hospital care; however, this needs to be explored in subsequent work. Most hospital based clinical decision support tools are designed to identify children who need a CT scan; although, this is not actually the clinical end point of interest. The reason for doing a CT scan is to seek intracranial injury that requires neurosurgery. Arguably, if a child receives no active treatment, then a CT scan is unnecessary, even if it does show intracranial injury [49] especially as paediatric CT scanning results in a significantly increased lifetime risk of radiation induced fatal malignancy [50]. The aim of a pre-hospital clinical decision support tool designed for use by paramedics (following additional research) will likely be to identify head-injured children who can safely be left at scene, and *do not* need conveyance. Therefore, the tool will "rule-out" serious head injury and be highly sensitive, since if all answers are negative in a highly sensitive tool, the tool can be used to rule out clinically significant brain injury. This is accepting that there are complicating issues specific to pre-hospital care such as local ambulance policy, time of day, time since injury, complex wound closure, safeguarding, parental concern, and parental capability, that need to be considered and may warrant conveyance, despite there being minimal concern of a clinically significant head injury [51].

All the existing hospital-based tools address diverse questions, age groups, injury severities, and outcomes and have been derived, evaluated, and validated in a different way. In comparison to CHALICE, CATCH and other hospital-based tools, PECARN identifies children with a very low risk of brain injury who *do not* need a scan, which is an important concept, because it considers who a clinician *does not* need to scan, rather than who they *do* [6]. PECARN was derived using two large multicentre cohorts with over 40,000 children and is extremely sensitive, particularly for infants. A tool designed for use by paramedics in the pre-hospital setting would likely aim to follow this approach and identify children who can safely be left at scene and *do not* need conveyance, due to sustaining a trivial or minor head injury that is very low risk of clinically significant brain injury, similarly to Kupperman's PECARN [6], though this needs further consideration and study. The role of ambulance paramedics includes the ability to make autonomous decisions on the safe discharge of patients at scene to prevent unnecessary conveyance to the ED. This is similar to in-hospital clinicians whose role it is to discharge patients from the ED when it is safe to do so. Therefore, the in-hospital clinical decision support tools for the assessment and management of children with head injury will share many similarities, particularly if the tools are being used in triage. PECARN is also the only tool that considers the age of the child from the beginning and thereafter asks different questions relevant to the child's age [6]. Paramedics have reported difficulty using the NICE head injury guidance [4] for this reason, as they could not ask younger children about amnesia, headache, and alcohol use [51].

Paramedic use of clinical decision support tools in current practice:

Previous research demonstrates that paramedics find clinical decision tools useful in identifying alternative patient pathways for other patient groups, such as older adults following a fall [52]. Clinical decision tools are not a new concept and have been at the forefront of digital health solutions for

For peer review only - http://bmjopen.bmj.com/site/about/guidelines.xhtml

more than twelve years [53]. In pre-hospital care, they are used by paramedics to manage risk and support referral to alternative care pathways and community-based care [54]. Additionally, the use of clinical decision tools in the pre-hospital setting by paramedics has been shown to significantly reduce the opportunity for human error, improve diagnostic accuracy, improve optimisation of resources, improve compliance with guidelines and improve overall quality and safety of care for patients [55] [56]. One systematic review of randomised controlled trials (RCT) compared clinical decision tools to usual care in the pre-hospital setting and concluded that there are too few RCTs to draw any firm conclusions in pre-hospital care, although there is a possibility that clinical decision tools increase diagnostic accuracy [57]. A more recent cluster RCT investigating the effectiveness of a clinical decision tool to increase the number of elderly fallers referred to a community falls team rather than ED, found evidence that paramedics use clinical decision tools to justify current practice rather than to inform decisions. The study found that paramedics decided to leave patients at home first, and then used the clinical decision tool to justify their decision. The tool was found to be cost effective and successful at changing paramedic practice [58].

What clinical criteria should be included:

All 18 clinical criteria identified in this systematic mapping review are important to consider for a pre-hospital tool designed to support paramedics in their assessment and management of children with head injury, however some may be difficult to operationalise in the pre-hospital setting. In addition, the hospital-based tools have varying cut off points for different clinical criteria and which descriptor a pre-hospital tool should use requires further consideration. It is important to acknowledge that all clinical decision tools should be used alongside clinical judgment. Whilst this review is intended to support the process of developing a clinical tool to support paramedic decision making at scene, it is only an initial step, and it is recognised that considerably more research is required. It would only be reasonable to adapt in-hospital tools for pre-hospital use if it could be demonstrated that the tool was applied with similar reliability and accuracy to that which is currently achieved in hospital practice.

Certainty of use:

It is clear from the evidence that some clinical predictors are significant for identifying a potentially clinically important traumatic brain injury following a head injury and should be included in a pre-hospital tool, such as a post-traumatic seizure [43]. Signs of a skull fracture was most likely to trigger a CT response over any other clinical criteria, since the risk of intracranial injury is significantly increased in the presence of a skull fracture. This is reflected in the NICE guidance for the management of head injury, which suggests a CT scan should be performed within an hour if there are signs of a skull fracture [4]. The tools indicated that specifically a 'large', or 'non-frontal' (particularly parietal) [19] or 'boggy' haematoma, especially in infants, is more indicative of significant intracranial injury, which is apparent in other research [57]. Under 6-month-olds have a higher likelihood of significant intracranial haemorrhage with haematomas [59]. Greenes and Schutzman (2001) [19] suggest that among asymptomatic head-injured infants, the risk of skull fracture and associated intracranial haemorrhage is correlated with both scalp haematoma size and location. In most ambulance trusts across the United Kingdom, local policy stipulates that any under 2-year-old should be conveyed to the ED, no matter how mild the presenting injury [51]. Abusive head trauma occurs more frequently in under 1's [60]; paramedics are in a unique position to identify suspected non accidental injury since they attend the patient's home environment often in a time of crisis and have a professional duty to protect vulnerable persons [61]. This evidence supports the recommendation of including 'infants' as a clinical predictor for a pre-hospital tool designed to aid decision making regarding conveyance, or to exclude under 1's entirely. Non accidental injury is only a clinical predictor in two in-hospital clinical

For peer review only - http://bmjopen.bmj.com/site/about/guidelines.xhtml

decision tools, however; perhaps this is because it is seen as a "no-brainer" and children were often excluded from the study cohort, as they would always need a scan. A child with suspected non accidental injury should always be conveyed to the ED by paramedics. Abuse tools such as those derived by Pfeiffer [27] and Cowley [24] include certain clinical predictors that can help paramedics in recognising non accidental injury.

Clinical predictors that may need intervention:

The pre-hospital environment is very different to in-hospital and there are some clinical predictors that may warrant conveyance as they may need interventions that cannot be done pre-hospitably, such as observation and complex wound closure. In addition, there are factors that are outside of a paramedic's scope of practice that presents too much risk to discharge at scene, and therefore warrants conveyance.

The number of vomits was variable across the tools; research suggests that children who vomit after a head injury is less significant than in adults and they do not necessarily have a serious brain injury [62]. Other literature supports this, suggesting that clinically important traumatic brain injuries are uncommon in children presenting with head injury with vomiting and a management approach of observation without immediate CT may be appropriate [63]. However, a period of observation is not always practical or possible for paramedics in the pre-hospital setting. This was reported by paramedics in a survey exploring reasons why paramedics convey children with minor head injury to the ED [51]. However, most children with head injury (90%) who are admitted for observation are discharged without any further treatment being given [64]. Notably, *persistent* vomiting post head injury is indicative of a subdural haematoma or skull fracture; Harper *et al.* (2020) [65] supports this, concluding that discreet vomiting over four times was a significant risk factor for intracranial injury. Additionally, vomiting combined with other clinical criteria such as headache and altered mental status, can indicate a more serious head injury [63]. This theme is clear with other clinical predictors such as loss of consciousness; children with minor blunt head trauma presenting to the ED with *isolated* loss of consciousness are at very low risk for clinically significant brain injury and do not routinely require CT scanning [66], however if the loss of consciousness was associated with other clinical predictors, the risk is increased. Any clinical predictor present in isolation is less likely to indicate clinically significant traumatic brain injury than the presence of multiple risk criteria [63]. This is similar for mechanism of injury; evidence implies that mechanism of injury alone is less predictive of an abnormal brain CT, compared with other clinical predictors [67]. Despite this, most paramedics would convey a head-injured child based solely on mechanism, despite them appearing well with no clinical concern [51].

Fowler *et al.* (2018) [68] found that attending to paediatric patients evokes anxiety and discomfort amongst paramedics, which often led to reluctance to initiate treatment and poorer care. This includes unnecessary transport to the ED. Approximately half of paramedic respondents in a survey reported feeling 'not at all confident' with completing a neurological assessment on a child following a head injury [51], despite a focal neurological deficit being a strong indicator of intracranial injury [33]. GCS is inherently difficult to assess in a younger child, however a reduced GCS is a high-risk factor for clinically significant intracranial injury [41].

Clinical predictors that infrequently result in intracranial injury:

There is limited evidence to suggest vertigo as a standalone clinical predictor should be included in a decision tool for conveyance of children with head injury, and this needs further investigation [20]. Additionally, evidence is still conflicting regarding the risk of intracranial haemorrhage in children with

For peer review only - http://bmjopen.bmj.com/site/about/guidelines.xhtml

a clotting impairment/anticoagulation, [69], despite this being a research priority since 2011 [70. Bressan *et al.* (2020) [69] found that although children with a bleeding disorder who have sustained a head injury more often received a CT scan, their risk of intracranial haemorrhage was low. Their findings are echoed in other literature [71]; CTs are obtained twice as often in children with bleeding disorders, although intracranial haemorrhage occurred in only 1.1% of these patients, and they had symptoms that would have prompted the need for a CT anyway. New guidance from NICE [4] advocates a CT head scan within eight hours for children on anticoagulant treatment, or within an hour if they have a bleeding/clotting disorder, independently of other indications. However, only one out of 31 children on anticoagulants in Bressen *et als.* (2020) [69] and Lee *et als*. (2011) [71] study combined, was diagnosed with an intracranial haemorrhage, and that patient very obviously needed a CT from the outset. These clinical criteria need further consideration before being included in a pre-hospital tool for paramedic use.

LIMITATIONS:

The role of mapping reviews is to provide a descriptive account of the published literature; therefore, this should be considered when assessing the findings of the overall evidence synthesis. A direct comparison of the clinical decision tools is not possible because they address different questions, age groups, injury severities, and outcomes. It is recognised that none of the evidence is from the pre-hospital environment, since there are no existing out-of-hospital clinical decision support tools designed to support the assessment and management children with head injury, hence the need for this research. Therefore, the in-hospital publications of tools considering childhood injury is the only research available to draw conclusions from at this point. Elements of in-hospital research can be attributed to pre-hospital care since there are similarities, however it is acknowledged that further research is required with pre-hospital staff in the pre-hospital environment following this work. Although rigorous methods of identifying all in-hospital tools have been followed, it is possible that some studies have been overlooked. This is particularly the case for international studies since this review included English language articles only.

CONCLUSIONS AND FUTURE RESEARCH:

Clinical predictors that markedly increase the likelihood of neurosurgical intervention and should be considered for inclusion in a clinical decision support tool for use by paramedics are:: signs of a skull fracture; a large, boggy or non-frontal haematoma (particularly in an infant); persistent discreet episodes of vomiting; a focal neurological deficit; GCS less than 15; a prolonged or worsening headache; a prolonged loss of consciousness; a post traumatic seizure; amnesia in older children; suspicion of non-accidental injury; drug or alcohol use and being under 1 year old. Clinical criteria that require further investigation include mechanism of injury; clotting impairment/anticoagulated; vertigo; length of time of loss of consciousness; number of vomits, and description of what constitutes a neurological deficit in younger children. Any clinical predictor present in isolation is unlikely to indicate clinically significant traumatic brain injury.

There are likely to be additional clinical criteria that are relevant to paramedic assessment and practice, which are not included in any of these tools. Such criteria would need to be identified and explored in future qualitative work involving interviewing paramedics about what influences them when deciding to convey children with minor head injury to the hospital. None of the existing hospital-based clinical decision support tools can be directly implemented into paramedic practice; however, elements from each of the tools can be adapted to create a new tool specific for paramedics in pre-hospital care. Future qualitative research is needed to investigate paramedics'

For peer review only - http://bmjopen.bmj.com/site/about/guidelines.xhtml

thoughts on a clinical decision tool designed to support them in their assessment and management of head-injured children. Additionally, future research utilising Delphi methods is required to build consensus amongst study matter experts and users to enable the development of a new clinical decision tool to support paramedics in safely assessing and managing children with minor head injury.

CONFLICT OF INTEREST:

None

No financial relationships with any organisations that might have an interest in the submitted work in the previous three years, no other relationships or activities that could appear to have influenced the submitted work.

COPYRIGHT:

The Corresponding Author has the right to grant on behalf of all authors and does grant on behalf of all authors, an exclusive licence (or non-exclusive for government employees) on a worldwide basis to the BMJ Publishing Group Ltd to permit this article (if accepted) to be published in BMJ editions and any other BMJPGL products and sublicenses such use and exploit all subsidiary rights, as set out in our licence

TRANSPARENCY STATEMENT:

This manuscript is an honest, accurate and transparent account of the study being reported. No important aspects of the study have been omitted and any discrepancies from the study as originally planned have been explained.

FUNDING SOURCE:

This work is supported by Health Education England/National Institute for Health Research Integrated Clinical and Practitioner (ICA) Programme's Doctoral Clinical and Practitioner Academic Fellowship (DCAF) Scheme, award number 25215. The views expressed are those of the authors and do not necessarily represent those of Health Education England, the National Institute for Health Research or Department of Health.

ETHICS APPROVAL:

Not required

DATA SHARING:

No additional data available

AUTOR CONTRIBUTOR STATEMENT:

JB, SV and ML supervised the work. AP wrote the protocol, conducted the review, and drafted the manuscript with input from JB, SV, ML, JeB and JS. JeB double screened the abstracts and full texts. PS helped to design the search terms and conduct the search. All authors interpreted and analysed the results. All authors discussed the results, contributed to and approved the final manuscript.

REFERENCES:

For peer review only - http://bmjopen.bmj.com/site/about/guidelines.xhtml

1. NHS (2019) The NHS Long term Plan. Available at: https://www.longtermplan.nhs.uk/ [Accessed 30th January 2023].
2. Arora, R., White, E.N., Niedbala, D., Ravichandran, Y., Sethuraman, U. and Radovic, N. (2020) Reducing Computed Tomography Scan Utilization for Paediatric Minor Head Injury in the Emergency Department: A Quality Improvement Initiative. *Academic Emergency Medicine,* 28(6), pp. 655-665.
3. Healthcare Quality Improvement Partnership (2015) Traumatic head injury in children and young people: a national overview. Available at: https://www.hqip.org.uk/wp-content/uploads/2018/02/traumatic-head-injury-in-children-and-young-people-a-national-overview.pdf [Accessed 30th January 2023].
4. National Institute for Health and Care Excellence (2023) *Head Injury Management.* Available at: https://cks.nice.org.uk/topics/head-injury/management/ [Accessed 23 May 2023].
5. Aldridge, P., Castle, H., Phillips, C., Russel, E., Guerrero-Luduena, R. and Rout, R. (2020). Head home: a prospective cohort study of a nurse led paediatric head injury clinical decision tool at a district general hospital, Emergency Medical Journal, 37(11), pp. 680-685.
6. Kuppermann, N., *et al.* (2009) Identification of children at very low risk of clinically-important brain injuries after head trauma: A prospective cohort study. *The Lancet,* 374(9696), pp. 1160-1170.
7. Campbell, F., Tricco, A.C., Munn, Z., Pollock, D., Saran, A., Sutton, A., White, H. and Khalil, H. (2023) Mapping reviews, scoping reviews, and evidence and gap maps: the same but different – the big picture review family, *Systematic reviews*, 12(45).
8. Grant, M.J. and Booth, A. (2009) A typology of reviews: an analysis of 14 review types and associated methodologies. *Health Info Library Journal,* 26(91), pp.108-9.
9. Clapton, J., Rutter, D. and Sharif, N. (2009) *Social Care Institute for Excellence (SCIE) systematic mapping guidance.* Available at: https://www.scie.org.uk/publications/researchresources/rr03.pdf [Accessed 30 January 2023].
10. Page, M.J., Mckenzie, J.E., Bossuyt, P.M., Hoffman, T.C. and Mulrow, C.D. (2020) The PRISMA 2020 statement: an updated guideline for reporting systematic reviews. BMJ, 172(71).
11. Covidence systematic review software, Veritas Health Innovation, Melbourne, Australia. Available at www.covidence.org [Accessed 18th April 2023].
12. Cooper, D. (2016). What is a “mapping study?” *Journal of the Medical Library Association,* 104(1), pp. 76-78.
13. Dunning, J., Daly, J., Lomas, J., Lecky, F., Batchelor, J. and Mackway-Jones (2006). Derivation of the children's head injury algorithm for the prediction of important clinical events decision rule for head injury in children. *Archives of Disease in Childhood,* 91(11), pp. 885-91.
14. Osmond, M.H (2010) CATCH: a clinical decision rule for the use of computed tomography in children with minor head injury, *Canadian Medical Association Journal,* 182(4).
15. Oman, J. A., Cooper, R. J., Holmes, J. F., Viccellio, P., Nyce, A., Ross, S. E., ... & Mower, W. R. (2006). Predictive performance of a decision rule to predict need for computed tomography among children with blunt head trauma. *Pediatrics,* 117(2), pp. 238-246.
16. Palchak, M.J *et al.* (2003). A decision rule for identifying children at low risk for brain injuries after blunt head trauma, *Annals of Emergency Medicine*, 42(4), pp. 492-506
17. Haydel, M.J. and Shembekar, A. D. (2003) Prediction of intracranial injury in children aged five years and older with loss of consciousness after minor head injury due to nontrivial mechanisms, *Annals of Emergency Medicine,* 42(4).
18. Atabaki, S. M., Stiell, I. G., Bazarian, J. J., Sadow, K. E., Vu, T. T., Camarca, M. A., ... & Chamberlain, J. M. (2008). A clinical decision rule for cranial computed tomography in minor

For peer review only - http://bmjopen.bmj.com/site/about/guidelines.xhtml

pediatric head trauma. *Archives of pediatrics and adolescent medicine,* 162(5), pp. 439-445

19. Greenes, D. and Schutzman, S. (2001) Clinical significance of scalp abnormalities in asymptomatic head-injured infants. *Paediatric Emergency Care,* 17(2), pp. 88-92.
20. Klemetti, S., Uhari, M., Pokka, T., & Rantala, H. (2009). Evaluation of decision rules for identifying serious consequences of traumatic head injuries in pediatric patients. Pediatric emergency care, 25(12), 811-815
21. Quayle, K. S., Jaffe, D. M., Kuppermann, N., Kaufman, B. A., Lee, B. C., Park, T. S., & McAlister, W. H. (1997). Diagnostic testing for acute head injury in children: when are head computed tomography and skull radiographs indicated?. *Pediatrics,* 99(5).
22. Dietrich, A. M., Bowman, M. J., Ginn-Pease, M. E., Kosnik, E., & King, D. R. (1993). Pediatric head injuries: can clinical factors reliably predict an abnormality on computed tomography?. *Annals of emergency medicine, 22*(10), pp. 1535-1540.
23. Güzel, A., Hiçdönmez, T., Temizöz, O., Aksu, B., Aylanç, H., & Karasalihoglu, S. (2009). Indications for brain computed tomography and hospital admission in pediatric patients with minor head injury: how much can we rely upon clinical findings?. *Pediatric neurosurgery,* 45(4), pp. 262-270.
24. Cowley, Laura E *et al.* (2018) Acceptability of the Predicting Abusive Head Trauma (PredAHT) clinical prediction tool: A qualitative study with child protection professionals, *Child Abuse & Neglect* 81():pp. 192-205.
25. Neumayer, Katie *et al.* (2020) Validation of the "CHIIDA" and application for PICU triage in children with complicated mild traumatic brain injury. *Journal of pediatric surgery*, 55(7), pp. 1255-1259
26. Greenberg, J., Yan, Y., Carpenter, C., Lumba-Brown, A., Keller, M., Pineda, J., Brownson, R. and Limbrick, D. (2018) Development of the CIDSS2 Score for Children with Mild Head Trauma without Intracranial Injury. *Journal of Neurotrauma,* 35(22), pp. 2699-2707.
27. Pfeiffer, H., Smith, A., Kemp, M., Cowley, E., Cheek, A., Dalziel, S., Borland, S., O'Brien, S., Bonisch, M., Neutze, J., Oakley, E., Crowe, L., Hearps, S., Lyttle, M., Bressan, S. and Babl, F. (2018) External validation of the PediBIRN clinical prediction rule for abusive head trauma. *Paediatrics,* 141(5).
28. Hess, Erik *et al.*(2014) Effectiveness of the head CT choice decision aid in parents of children with minor head trauma: study protocol for a multicentre randomized trial. *Trials,* 15(1), pp. 253-253.
29. Unden, J. *et al.* (2018) External validation of the Scandinavian guidelines for management of minimal, mild and moderate head injuries in children, *BMC Medicine,* 16(176).
30. Schwartz, Jamie *et al.* (2020) Applying Pediatric Brain Injury Guidelines at a Level I Adult/Pediatric Safety-Net Trauma Center. *The Journal of surgical research,* 255, pp. 106-110
31. Yogo, N., Toida, C., Muguruma, T., Gakumazawa, M., Shinohara, M. and Takeuchi, I. (2021) Simplified Clinical Decision Rule Using Clinically Important Events for Risk Prediction in Pediatric Head Injury: A Retrospective Cohort Study, *Journal of Clinical Medicine,* 10(22).
32. Brittany M. Stopa*, et al.* (2019) Comparison of minor head trauma management in the emergency departments of a United States and Italian Children's hospital, *Italian Journal of Pediatrics,* 45(24).
33. Da Dalt, L. *et al.*(2006) Predictors of intracranial injuries in children after blunt head trauma. *European Journal of Paediatrics,* 165(3), pp. 142–8.
34. Buchanich, J. M. (2007). A clinical decision-making rule for mild head injury in children less than three years old (Doctoral dissertation, University of Pittsburgh).

For peer review only - http://bmjopen.bmj.com/site/about/guidelines.xhtml

35. Greenberg *et al*. (2021) Development and external validation of the KIIDS-TBI tool for managing children with mild traumatic brain injury and intracranial injuries. *Academic Emergency Medicine,* 28(12).
36. Bressan, Silvia *et al.* (2012) Implementation of adapted PECARN decision rule for children with minor head injury in the paediatric emergency department. *Academic emergency medicine: official journal of the Society for Academic Emergency Medicine*, 19(7), pp. 801-807.
37. Babl, F. E., Oakley, E., Dalziel, S.R., Crowe, L., Bressan, S. and Lyttle, M. (2018) Accuracy of clinician practice compared with three head injury decision rules in children: a prospective cohort study, Annals of Emergency Medicine, 71 (6).
38. Pfeiffer, H., Smith, A., Kemp, M., Cowley, E., Cheek, A., Dalziel, S., Borland, S., O'Brien, S., Bonisch, M., Neutze, J., Oakley, E., Crowe, L., Hearps, S., Lyttle, M., Bressan, S. and Babl, F. (2018) External validation of the PediBIRN clinical prediction rule for abusive head trauma. Paediatrics, 141(5).
39. Babl, F.E et al. (2014) Head injury decision rules in children. Lancet, 390, pp. 1488.
40. Gupta, M., William, M., Rodriguez, R. and Hendey, G. (2018) Validation of the Pediatric NEXUS II Head Computed Tomography Decision Instrument for Selective Imaging of Pediatric Patients with Blunt Head Trauma. *Academic Emergency Medicine,* 25(7), pp. 729-737.
41. Pickering, A., Harnan, S., Fitzgerald, P., Pandor, A. and Goodacre, S. (2011) Clinical decision rules for children with minor head injury: a systematic review, Archives of Disease in Childhood, 95(5).
42. Doll, E., Gong, P., Sowell, M. and Evanczyk, L. (2021) Post-traumatic Headache in Children and Adolescents, Current Pain and Headache Reports, 25(51).
43. Fordington, S. and Manford, M. (2020) A review of seizures and epilepsy following traumatic brain injury, Journal of Neurology, 267(10).
44. Bressan, S.B., Romanato, S., Mion, T., Zanconato, S. and Da Dalt, L. (2012) Implementation of adapted PECARN decision rule for children with minor head injury in the paediatric emergency department, Academic Emergency Medicine, 19 (7).
45. Hymel, K.P., Fingarson, A.K., Pierce, M.C., Kaczor, K., Makoroff, K.L. and Wang, M. (2022) External Validation of the PediBIRN Screening Tool for Abusive Head Trauma in Paediatric Emergency Department Settings, Paediatric Emergency Medicine, 38(6).
46. Ide, K., Uematsu, S., Tetsuhara, K., Yoshimura, S. and Kato, T. (2016) External Validation of the PECARN Head Trauma Prediction Rules in Japan, Academic Emergency Medicine, 24(3), pp. 308-314.
47. Weil, Z.M., Corrigan, J.D., Karelina, K. (2018) Alcohol use disorder and traumatic brain injury, Alcohol Research, 39(2), pp. 171-180.
48. Lin, H., Lin, T., Soo, K., Chen, C., Kuo, L., Lin, Y., Lee, W. and Lin, C. (2014) The Effect of Alcohol Intoxication on Mortality of Blunt Head Injury, Biomed Research International.
49. Kambal, M. A., Abou, E.M., Gadi, I, A., Boukai, A. A., Jurayyan, N.A. and Babiker, A.M. (2014). Managing traumatic brain injury in children: When do we need a computed tomography of the head? Sudanese Journal of Paediatrics, 14(1).
50. Meulepas, J.D. et al. (2019) Radiation Exposure from Paediatric CT scans and Subsequent Cancer Risk in the Netherlands. Journal of the National Cancer Institute, 111(3), pp. 256-263.
51. Proctor, A., Voss, S., Lyttle, M.D. and Benger, J. (2023) Assessment and management of children with head injury: practice and opinion, *Journal of Paramedic Practice,* 15(2).
52. Oosterwold, J., Sagel, D., Berben, S, Roodbol, P. and Broekhuis, M. (2018) Factors influencing the decision to convey or not to convey elderly people to the emergency department after emergency ambulance attendance: a systematic mixed studies review. BMJ Open.

For peer review only - http://bmjopen.bmj.com/site/about/guidelines.xhtml

53. Mann, D., Hess, R. and Feldstein, A. (2019) Adaptive design of a clinical decision support tool: What the impact on utilization rates means for future CDS research. Digital Health, 5.
54. Porter, A., Dale, J., Foster, T., Logan, P., Wells, B. and Snooks, H. (2018) Implementation and use of computerised clinical decision support (CCDS) in emergency pre-hospital care: a qualitative study of paramedic views and experience using Strong Structuration Theory. BMC, 13(91)
55. Yeats, A. (2018). *Using Clinical Decision Support to Reduce Clinical Risk.* Available at: https://www.oneadvanced.com/news-and-opinion/using-clinical-decision-support-to-reduce-clinical-risk/- [Accessed 05 July 2022].
56. Bashiri, A., Savareh, B.A. and Ghazisaeed, M. (2019) Promotion of prehospital emergency care through clinical decision support systems: opportunities and challenges, Clinical and Experimental Emergency Medicine, 6(4), pp. 288-296.
57. Hagiwara, M., Henricson, M., Jonsson, A. and Suserud, B. (2011). Decision support tools in pre-hospital care: a systematic review of randomised controlled trials.
58. Snooks, H., Carter, B. and Dale, J. (2014) Support and Assessment for Fall Emergency Referrals (SAFER 1): Cluster Randomised Trial of Computerised Clinical Decision Support for Paramedics, PLOS One.
59. Burns, E. (2016) Scalp Hematoma Characteristics Associated with Intracranial Injury in Paediatric Minor Head Injury. Academic Emergency Medicine, 23(5), pp. 577-583.
60. Araki, T., Yokota, H., and Morita, A. (2017) Paediatric traumatic brain injury: characteristic features, diagnosis, and management, Neurologia medico-chirurgica, 57(2), pp. 82-93.
61. Moritz, D., Ebbs, P. and Carver, H. (2020) Paramedic ethics, capacity and the treatment of vulnerable patients. Journal of Paramedic Practice.
62. Dayan, P.S., Holmes, J.F., Atabaki, S., Miskin, M. and Kupperman, N. (2014) Association of Traumatic Brain Injuries with Vomiting in Children with Blunt Head Trauma, Annals of Emergency Medicine, 63(6), pp. 657-665.
63. Borland, M.L., et al. (2018) Delayed presentations to emergency departments of children with head injury: a predict study. Annals of Emergency Medicine, 74(1), pp. 1-10.
64. Crowe, L., et al., The epidemiology of paediatric head injuries: data from a referral centre in Victoria, Australia. Journal of paediatrics and child health, 2009. 45(6): p. 346-350.
65. Harper, J.A., Klassen, T.P., Balshaw, R., Dyck, J. and Osmond, M.H. (2020) Characteristics of vomiting as a predictor of intracranial injury in paediatric minor head injury, Canadian Journal of Emergency Medicine, 22(6).
66. Lee, L.K., Monroe, D., Bachman, M.C., Glass, T.F., Mahajan, P.V., Cooper, A., Stanley, R.M., Miskin, M., Dayan, P.S., Holmes, J.F., Kuppermann, N. (2014) Traumatic Brain Injury (TBI) Working Group of Pediatric Emergency Care Applied Research Network (PECARN). Isolated loss of consciousness in children with minor blunt head trauma. JAMA Pediatr, 168(9), pp. 837-43.
67. Lockie et al. (2013) Triggers for head computed tomography following paediatric head injury: Comparison of physicians' reported practice and clinical decision rules, Emergency Medicine Australia, 25, pp. 75-82.
68. Fowler, J., Beovich, B. and Williams, B. (2018) Improving paramedic confidence with paediatric patients: A scoping review. 15(1).
69. Bressan, S., Monagle, P., Dalziel, S., Borland, M., Phillips, N., Kochar, A., Lyttle, M., Cheek, J., Neutze, j., Oakley, E., Dalton, S., Gilhotra, Y., Hearps, S., Furyk, J. and Babl, F. (2020) Risk of traumatic intracranial haemorrhage in children with bleeding disorders, Journal of Paediatrics and Child Health, 56(12), pp. 1839-2003.

For peer review only - http://bmjopen.bmj.com/site/about/guidelines.xhtml

70. Pandor, A., Goodacre, S., Harnan, S., Holmes, M., Pickering, A., Fitzgerald, P., Rees, A. and Stevenson, M. (2011) Diagnostic management strategies for adults and children with minor head injury: a systematic review and an economic evaluation, Health Technology Assessment. 15(27).
71. Lee, L., Dayan, P. Gerardi, M., Borgialli, d., Badawy, M., Callahan, J., Lillis, K., Stanley, R., Gorelick, M., Dong, L., Zuspan, S., Holmes, J. and Kupperman, N. (2011) Intracranial haemorrhage after blunt head trauma in children with bleeding disorders, The Journal of Paediatrics, 158(6), pp. 1003-1008.

FIGURE LEGEND:

Figure 1- Prisma flow chart

WORD COUNT (excluding tables and references):

4,100

KEYWORDS:

Systematic review; head injuries; paramedic; children.

For peer review only

Figure 1.0 PRISMA flow diagram

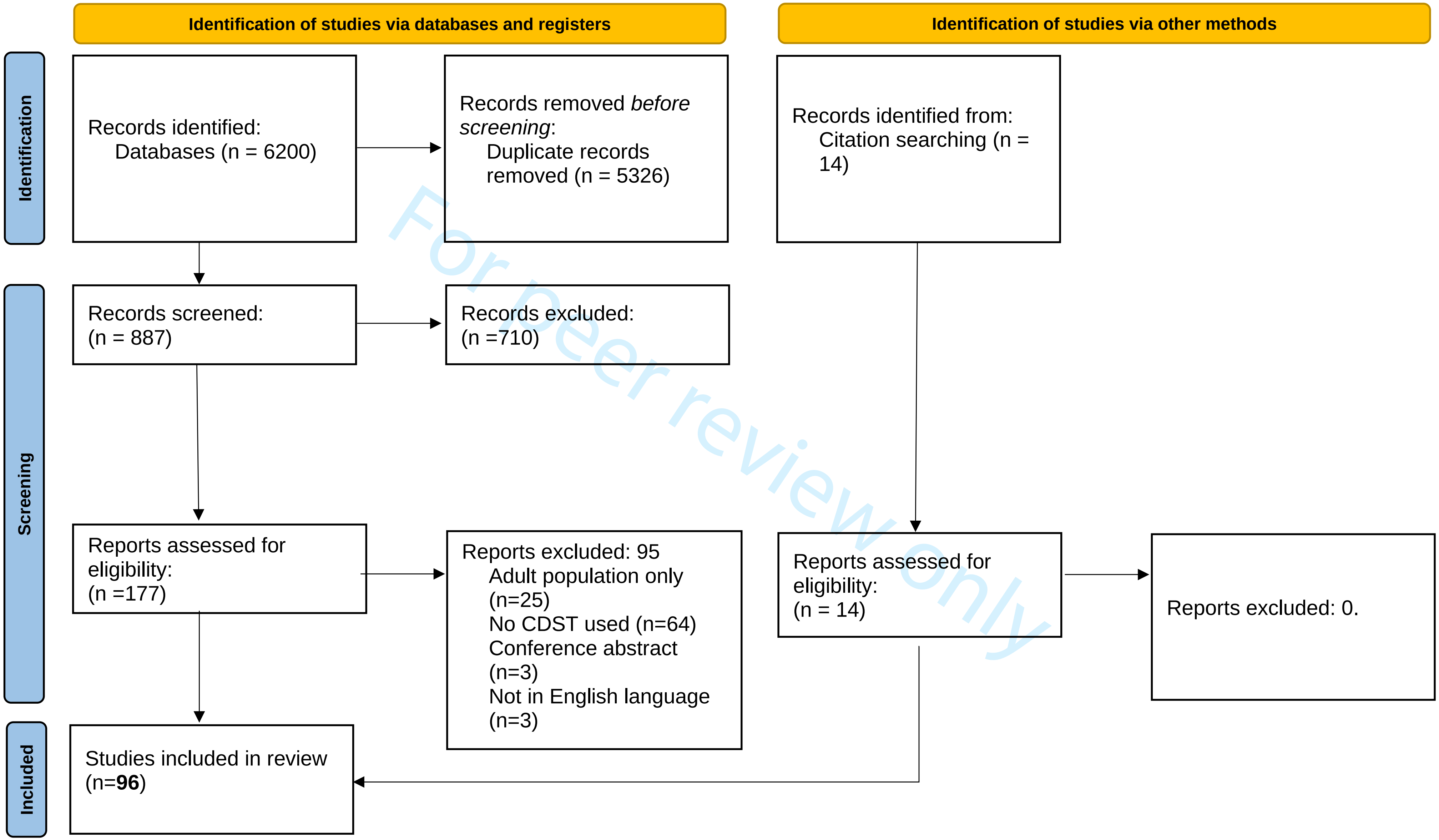

From: Page MJ, McKenzie JE, Bossuyt PM, Boutron I, Hoffmann TC, Mulrow CD, et al. The PRISMA 2020 statement: an updated guideline for reporting systematic reviews. BMJ 2021;372:n71. doi: 10.1136/bmj.n71. For more information, visit: http://www.prisma-statement.org/

For peer review only

Supplementary Table 1. Categories in each tool

	Signs of Skull fracture	Scalp Haematoma	Vomiting	Neuro Deficit	Headache	LOC	AMS	GCS	Seizure	Severe mechanism of injury	Drowsy	Amnesia	Vertigo	Non accidental injury	Clotting impairment/ anticoagulated	Drug/ alcohol use	Patient alert and well	Age
PECARN (2-18)	YES	NO	YES	NO	YES	YES	YES	YES	NO	YES	YES	NO	NO	NO	NO	NO	NO	NO
PECARN (<2)	YES	YES	NO	NO	NO	YES	YES	YES	NO	YES	YES	NO	NO	NO	NO	NO	YES	YES
CHALICE	YES	YES	YES	YES	NO	YES	NO	YES	YES	YES	YES	YES	NO	YES	NO	NO	NO	NO
CATCH	YES	YES	NO	NO	YES	YES	YES	YES	NO	YES	NO	YES	NO	NO	NO	NO	NO	NO
NEXUS 2	YES	YES	YES	YES	NO	YES	YES	YES	NO	NO	YES	YES	NO	NO	YES	NO	NO	NO
HIDATq	YES	YES	YES	YES	NO	YES	YES	YES	YES	YES	YES	YES	NO	YES	YES	NO	YES	YES
PALCHACK	YES	YES	YES	NO	YES	NO	YES	NO	NO	NO	NO	NO	NO	NO	NO	NO	NO	NO
HAYDEL	NO	YES	YES	NO	YES	NO	NO	NO	YES	NO	NO	YES	NO	NO	NO	YES	NO	YES
ATABAKI	YES	YES	YES	YES	YES	YES	YES	YES	YES	YES	YES	YES	YES	NO	NO	YES	NO	YES
GREENES	YES	YES	NO	NO	NO	NO	NO	NO	NO	NO	NO	NO	NO	NO	NO	NO	NO	NO
KLEMETTI	YES	YES	NO	YES	NO	YES	YES	NO	NO	NO	NO	NO	YES	NO	NO	NO	NO	NO
QUAYLE	YES	NO	NO	YES	NO	YES	YES	NO	YES	NO	NO	NO	NO	NO	NO	NO	NO	NO
DIETRICH	NO	NO	YES	YES	YES	YES	NO	NO	YES	NO	NO	NO	NO	NO	NO	NO	NO	NO
GUZEL	YES	YES	NO	YES	YES	YES	NO	NO	YES	YES	NO	YES	NO	NO	NO	NO	NO	NO
PredAHT	NO	YES	NO	NO	NO	NO	NO	NO	YES	NO	NO	NO	NO	NO	NO	NO	NO	NO
CHIDA	YES	NO	NO	NO	NO	NO	NO	YES	NO	NO	NO	NO	NO	NO	NO	NO	NO	NO
CIDSS2	YES	NO	NO	YES	NO	NO	NO	YES	YES	NO	NO	NO	NO	NO	NO	YES	NO	NO
PediBIRN	YES	YES	NO	NO	NO	NO	NO	NO	NO	NO	NO	NO	NO	NO	NO	NO	NO	NO
HEAD CT CHOICE	NO	NO	YES	YES	YES	NO	YES	YES	NO	NO	YES	NO	NO	NO	NO	NO	NO	NO
SNC GUIDELINE	YES	YES	YES	YES	YES	YES	YES	YES	YES	NO	NO	NO	NO	NO	YES	NO	NO	NO
BIG-1	YES	NO	NO	YES	NO	YES	NO	NO	NO	NO	NO	NO	NO	NO	NO	YES	NO	NO
NOVEL CDR	NO	NO	NO	NO	YES	YES	YES	YES	YES	YES	NO	NO	NO	NO	NO	NO	NO	NO
HEAD TRAUMA EBG	YES	YES	YES	YES	NO	YES	YES	YES	NO	YES	NO	NO	NO	NO	NO	NO	NO	YES
DA DALT	YES	NO	YES	YES	YES	YES	YES	YES	YES	NO	YES	YES	NO	NO	NO	NO	NO	NO
BUCHANIC	YES	YES	YES	YES	YES	NO	YES	NO	NO	NO	NO	NO	NO	NO	NO	NO	NO	NO
KIIDS-TBI CDS	YES	YES	NO	NO	NO	NO	NO	YES	NO	NO	NO	NO	NO	NO	NO	NO	NO	NO

Supplementary Table 2. Performance accuracy of each tool.

Name of tool	Internal validity	External validity	Implementation/ evaluation	Performance accuracy
**PECARN (under 2)**	Positive evidence	Positive evidence	Mixed evidence supporting potential effect.<br><br>Mixed evidence supporting positive conclusion for implementation.	Sensitivity: 100%<br><br>Specificity: between 53% and 60%
**PECARN (2-18)**	Positive evidence	Positive evidence	Mixed evidence supporting potential effect.<br><br>Mixed evidence supporting positive conclusion for implementation.	Sensitivity: 96.8%<br><br>Specificity: between 53% and 60%
**CHALICE**	Positive evidence	Positive evidence	Mixed evidence supporting positive conclusion for implementation.	Sensitivity: 98%<br><br>Specificity: 86%
**CATCH**	Positive evidence	Positive evidence	Not evaluated for usability, potential effect, or post implementation impact.	Sensitivity: 98%<br><br>Specificity: 50%
**NEXUS II**	Positive evidence	Mixed evidence supporting positive conclusion	Not evaluated for usability, potential effect, or post implementation impact.	Sensitivity: 99%<br><br>Specificity: 15%
**HIDATq**	Positive evidence	Not reported	Not evaluated in a published study after development	Negative predictive value for CT was 100% for intracranial injury. The

For peer review only - http://bmjopen.bmj.com/site/about/guidelines.xhtml

Supplementary Table 2. Performance accuracy of each tool.

				positive predictive value of the tool was low.
**PALCHACK**	Positive evidence	Positive evidence	Not evaluated in a published study after development	Sensitivity: 100%<br><br>Specificity: 46%
**HAYDEL**	Positive evidence	Not reported	Not evaluated in a published study after development	Sensitivity: 100%<br><br>Specificity: 24%
**ATABAKI**	Positive evidence	Not reported	Not evaluated in a published study after development	Sensitivity: 100%<br><br>Specificity: 46%
**BUCHANICH**	Positive evidence	Not reported	Not evaluated in a published study after development	Sensitivity: 100%<br><br>Specificity: 40%
**DA DALT**	Developed but not tested for internal validity	Not reported	Not evaluated in a published study after development	Sensitivity: 100%<br><br>Specificity: 87%
**GREENES**	Developed but not tested for internal validity	Not reported	Not evaluated in a published study after development	Sensitivity: 53%<br><br>Specificity: 72%
**KLEMETTI**	Developed but not tested for internal validity	Not reported	Not evaluated in a published study after development	Sensitivity: 94%<br><br>Specificity: 29%
**QUAYLE**	Developed but not tested for internal validity	Not reported	Not evaluated in a published study after development	Sensitivity: 44%<br><br>Specificity: 85%

For peer review only

Supplementary Table 2. Performance accuracy of each tool.

**DIETRICH**	Developed but not tested for internal validity	Not reported	Not evaluated in a published study after development	Sensitivity: 100%<br><br>Specificity: 17%
**GUZEL**	Developed but not tested for internal validity	Not reported	Not evaluated in a published study after development	Sensitivity: 69%<br><br>Specificity: 43%
**PredAHT 2**	Positive evidence	Positive evidence	Not evaluated for usability, potential effect, or post implementation impact.	Sensitivity: 72%<br><br>Specificity: 86%
**CHIDA**	Negative evidence	Not reported	Not evaluated in a published study after development	Sensitivity: 94%<br><br>Specificity: 69%
**CIDSS2**	Positive evidence	Not reported	Not evaluated in a published study after development	Sensitivity: 51%<br><br>Specificity: 96%
**PediBIRN**	Positive evidence	Positive evidence	Not evaluated in a published study after development	Sensitivity: 96%<br><br>Specificity: 29%

For peer review only

Supplementary Table 2. Performance accuracy of each tool.

**Head CT Choice**	Positive evidence	Not reported	Not evaluated in a published study after development	Not reported
**SNC guideline**	Positive evidence	Positive evidence	Not evaluated in a published study after development	Sensitivity: 95%<br><br>Specificity: 59%
**BIG-1**	Positive evidence	Not reported	Not evaluated in a published study after development	Not reported
**Novel simplified CDR**	Positive evidence	Not reported	Not evaluated in a published study after development	Sensitivity: 80%<br><br>Specificity: 51%
**Head trauma EBG algorithm**	Positive evidence	Not reported	Not evaluated in a published study after development	Not reported
**KIIDS-TBI CDS**	Positive evidence	Positive evidence	Not evaluated in a published study after development	Sensitivity: 100%<br><br>Specificity: 26%

For peer review only

Supplementary Table 2. Performance accuracy of each tool.

For peer review only

#	Query	Limiters/Expanders	Last Run Via	Results
S1	(("Clinical decision support tool" OR "Clinical decision tool" OR "Clinical decision rule" OR "Diagnostic accuracy tool" OR "Triage tool" OR "Hospital-based tool" OR CDRs OR Intervention OR "Clinical decision score" OR "Decision Support Technique*") AND ("Head injury" OR "Minor head injury" OR "Traumatic brain injury" OR "Head trauma" OR "Head wound" OR "Intracranial injury") AND (Children OR Child OR Pediatric OR Paediatric OR Baby OR Babies OR Infant OR Schoolchild* OR Adolescent OR Teenage* OR "Young person")) AND ("emergency department" OR "trauma center" OR ED OR "A&E" OR "accident and emergency" OR "Emergency Service, Hospital")	Search modes - Boolean/Phrase	Interface - EBSCOhost Research Databases Search Screen - Advanced Search Database - CINAHL Plus;AMED - The Allied and Complementary Medicine Database;MEDLINE;APA PsycInfo	887

https://ezproxy.uwe.ac.uk/login?url=https://search.ebscohost.com/login.aspx?direct=true&db=jlh&db=amed&db=cmedm&db=psyh&bquery=((%26quot%3bClinical+decision+support+tool%26quot%3b+OR+%26quot%3bClinical+decision+tool%26quot%3b+OR+%26quot%3bClinical+decision+rule%26quot%3b+OR+%26quot%3bDiagnostic+accuracy+tool%26quot%3b+OR+%26quot%3bTriage+tool%26quot%3b+OR+%26quot%3bHospital-based+tool%26quot%3b+OR+CDRs+OR+Intervention+OR+%26quot%3bClinical+decision+score%26quot%3b+OR+%26quot%3bDecision+Support+Technique*%26quot%3b)+AND+(%26quot%3bHead+injury%26quot%3b+OR+%26quot%3bMinor+head+injury%26quot%3b+OR+%26quot%3bTraumatic+brain+injury%26quot%3b+OR+%26quot%3bHead+trauma%26quot%3b+OR+%26quot%3bHead+wound%26quot%3b+OR+%26quot%3bIntracranial+injury%26quot%3b)+AND+(Children+OR+Child+OR+Pediatric+OR+Paediatric+OR+Baby+OR+Babies+OR+Infant+OR+Schoolchild*+OR+Adolescent+OR+Teenage*+OR+%26quot%3bYoung+person%26quot%3b))+AND+(%26quot%3bemergency+department%26quot%3b+OR+%26quot%3btrauma+center%26quot%3b+OR+ED+OR+%26quot%3bA%26amp%3bE%26quot%3b+OR+%26quot%3baccident+and+emergency%26quot%3b+OR+%26quot%3bEmergency+Service%2c+Hospital%26quot%3b)&type=1&searchMode=Standard&site=ehost-live

Link to full search strategy.

KIN 4400 Independent Research Study in Kinesiology | PRISMA 2020 Checklist

Section and Topic	Item #	Checklist item	Location where item is reported
**TITLE**			
Title	1	Identify the report as a literature review.	Page 1
**ABSTRACT**			
Abstract	2	Provide a structured summary including, as applicable: background; objectives; data sources; study eligibility criteria, participants, and interventions; study appraisal and synthesis methods; results; limitations; conclusions and implications of key findings.<br>See the PRISMA 2020 for Abstracts checklist for the complete list.	1 and 2
**INTRODUCTION**			
Rationale	3	Describe the rationale for the review in the context of existing knowledge, i.e., what is already known about your topic.	2 and 3
Objectives	4	Provide an explicit statement of the objective(s) or question(s) the review addresses with reference to participants, interventions, comparisons, outcomes, and study design (PICOS).	3
**METHODS**			
Eligibility criteria	5	Specify the inclusion and exclusion criteria for the review and how studies were grouped for the syntheses with study characteristics (e.g., PICOS, length of follow-up) and report characteristics (e.g., years considered, language, publication status) used as criteria for eligibility, giving rationale.	4
Information sources	6	Specify all databases, registers, websites, organisations, reference lists and other sources searched or consulted to identify studies. Specify the date when each source was last searched or consulted.	3
Search strategy	7	Present the full search strategies for all databases, registers and websites, including any filters and limits used.	3,4,5,6
Selection process	8	State the process for selecting studies (i.e., screening, eligibility).<br>Specify the methods used to decide whether a study met the inclusion criteria of the review, including how many reviewers screened each record and each report retrieved, whether they worked independently, and if applicable, details of automation tools used in the process.	3,4,5,6
Study risk of bias assessment	11	Specify the methods used to assess risk of bias in the included studies, including details of the tool(s) used, how many reviewers assessed each study and whether they worked independently, and if applicable, details of automation tools used in the process.	3,4,5
**RESULTS**			
Study selection	16a	Describe the results of the search and selection process, from the number of records identified in the search to the number of studies included in the review, ideally using a flow diagram.	6

The checklist has been adapted for KIN 4400 Independent Research Study in Kinesiology at the University of Guelph-Humber. Last updated: Dec 9, 2021

Adapted From: Page MJ, McKenzie JE, Bossuyt PM, Boutron I, Hoffmann TC, Mulrow CD, et al. The PRISMA 2020 statement: an updated guideline for reporting systematic reviews. BMJ 2021;372:n71. doi: 10.1136/bmj.n71 *Adapted From:* Page MJ, McKenzie JE, Bossuyt PM, Boutron I, Hoffmann TC, Mulrow CD, et al. The PRISMA 2020 statement: an updated guideline for reporting systematic reviews. BMJ 2021;372:n71. doi: 10.1136/bmj.n71

For more information, visit: http://www.prisma-statement.org/

For peer review only - http://bmjopen.bmj.com/site/about/guidelines.xhtml

KIN 4400 Independent Research Study in Kinesiology | PRISMA 2020 Checklist

Section and Topic	Item #	Checklist item	Location where item is reported
	16b	Cite studies that might appear to meet the inclusion criteria, but which were excluded, and explain why they were excluded.	6
Study characteristics	17	Cite each included study and present its characteristics (e.g., study size, PICOS, follow-up period).	6
Risk of bias in studies	18	Present assessments of risk of bias for each included study.	N/A
Results of individual studies	19	For all outcomes, present, for each study: (a) summary statistics for each group (where appropriate) and (b) an effect estimate and its precision (e.g. confidence/credible interval), ideally using structured tables or plots.	6,7,8,9,13,14,15,16
**DISCUSSION**			
Discussion	23a	Provide a general interpretation of the results in the context of other evidence.	20
	23b	Discuss any limitations of the evidence included in the review.	23
	23c	Discuss any limitations of the review processes used.	23
	23d	Discuss implications of the results for practice, policy, and future research.	23
**OTHER INFORMATION**			
Registration and protocol	24a	Provide registration information for the review, including register name and registration number, or state that the review was not registered.	3
	24b	Indicate where the review protocol can be accessed, or state that a protocol was not prepared.	3
	24c	Describe and explain any amendments to information provided at registration or in the protocol.	N/A
Support	25	Describe sources of financial or non-financial support for the review, and the role of the funders or sponsors in the review.	28
Competing interests	26	Declare any competing interests of review authors.	27
Availability of data, code, and other materials	27	Report which of the following are publicly available and where they can be found: template data collection forms; data extracted from included studies; data used for all analyses; analytic code; any other materials used in the review.	n/a

The checklist has been adapted for KIN 4400 Independent Research Study in Kinesiology at the University of Guelph-Humber. Last updated: Dec 9, 2021

Adapted From: Page MJ, McKenzie JE, Bossuyt PM, Boutron I, Hoffmann TC, Mulrow CD, et al. The PRISMA 2020 statement: an updated guideline for reporting systematic reviews. BMJ 2021;372:n71. doi: 10.1136/bmj.n71 *Adapted From:* Page MJ, McKenzie JE, Bossuyt PM, Boutron I, Hoffmann TC, Mulrow CD, et al. The PRISMA 2020 statement: an updated guideline for reporting systematic reviews. BMJ 2021;372:n71. doi: 10.1136/bmj.n71

For more information, visit: http://www.prisma-statement.org/

BMJ Open

Which elements of hospital-based clinical decision support tools for the assessment and management of children with head injury can be adapted for use by paramedics in pre-hospital care? A systematic mapping review and narrative synthesis.

Journal:	*BMJ Open*
Manuscript ID	bmjopen-2023-078363.R3
Article Type:	Original research
Date Submitted by the Author:	22-Dec-2023
Complete List of Authors:	Proctor, Alyesha; University of the West of England,<br>Lyttle, Mark; University of the West of England Bristol, Faculty of Health and Applied Sciences<br>Billing, Jedd; University of the West of England<br>Shaw, Pauline; University of the West of England<br>Simpson, Julian; University of the West of England<br>Voss, Sarah; University of the West of England, Health and Life Sciences<br>Benger, Jonathan; The University Hospitals NHS Foundation trust, Academic Department of Emergency care; The University of the West of England, Faculty of Health & Life Sciences
**Primary Subject Heading**:	Emergency medicine
Secondary Subject Heading:	Paediatrics
Keywords:	Systematic Review, PAEDIATRICS, ACCIDENT & EMERGENCY MEDICINE

SCHOLARONE™
Manuscripts

I, the Submitting Author has the right to grant and does grant on behalf of all authors of the Work (as defined in the below author licence), an exclusive licence and/or a non-exclusive licence for contributions from authors who are: i) UK Crown employees; ii) where BMJ has agreed a CC-BY licence shall apply, and/or iii) in accordance with the terms applicable for US Federal Government officers or employees acting as part of their official duties; on a worldwide, perpetual, irrevocable, royalty-free basis to BMJ Publishing Group Ltd ("BMJ") its licensees and where the relevant Journal is co-owned by BMJ to the co-owners of the Journal, to publish the Work in this journal and any other BMJ products and to exploit all rights, as set out in our licence.

The Submitting Author accepts and understands that any supply made under these terms is made by BMJ to the Submitting Author unless you are acting as an employee on behalf of your employer or a postgraduate student of an affiliated institution which is paying any applicable article publishing charge ("APC") for Open Access articles. Where the Submitting Author wishes to make the Work available on an Open Access basis (and intends to pay the relevant APC), the terms of reuse of such Open Access shall be governed by a Creative Commons licence – details of these licences and which Creative Commons licence will apply to this Work are set out in our licence referred to above.

Other than as permitted in any relevant BMJ Author's Self Archiving Policies, I confirm this Work has not been accepted for publication elsewhere, is not being considered for publication elsewhere and does not duplicate material already published. I confirm all authors consent to publication of this Work and authorise the granting of this licence.

For peer review only

TITLE:

Which elements of hospital-based clinical decision support tools for the assessment and management of children with head injury can be adapted for use by paramedics in pre-hospital care? A systematic mapping review and narrative synthesis.

AUTHORS:

Corresponding author: Alyesha Proctor[1]

Dr Mark D Lyttle[1]

Jedd Billing[1]

Pauline Shaw[1]

Julian Simpson[1]

Professor Sarah Voss[1]

Professor Jonathan Benger[1]

[1]Faculty of Health and Applied Sciences, University of the West of England, Glenside Campus (1H30), Blackberry Hill, Bristol, BS16 1DD, England

Alyesha.proctor@uwe.ac.uk (NIHR Clinical Doctoral Research Fellow, Advanced Paramedic Practitioner)

Mark.Lyttle@uwe.ac.uk (Senior Research Fellow, Consultant in Paediatric Emergency Medicine)

Jedd.billing@uwe.ac.uk (Senior Lecturer in Paramedic Science)

Pauline.shaw@uwe.ac.uk (Subject Librarian)

Julian.simpson@uwe.ac.uk (Programme Lead, Senior Lecturer in Paramedic Science)

Sarah.voss@uwe.ac.uk (Professor of Emergency Care)

Jonathan.benger@uwe.ac.uk (Professor of Emergency Care, Consultant in Emergency Medicine)

*Corresponding author address: Alyesha Proctor, Glenside Campus, Blackberry Hill, Stapleton, Bristol, BS16 1DD.

ABSTRACT:

Objective:

Hospital-based clinical decision tools support clinician decision making when a child presents to the Emergency Department with a head injury, particularly regarding computed tomography scanning. However, there is no decision tool to support pre-hospital clinicians in deciding which head injured children can safely remain at scene. This study aims to identify clinical decision tools, or constituent elements, which may be adapted for use in pre-hospital care.

Design:

Systematic mapping review and narrative synthesis.

Data sources:

For peer review only - http://bmjopen.bmj.com/site/about/guidelines.xhtml

For peer review only

Searches were conducted using MEDLINE, EMBASE, PsycINFO, CINAHL and AMED.

Eligibility criteria:

Quantitative, qualitative, mixed methods or systematic review research that included a clinical decision support tool for assessing and managing children with head injury.

Data extraction and synthesis:
We systematically identified all in-hospital clinical decision support tools and extracted from these the clinical criteria used in decision making. We complemented this with a narrative synthesis.

Results:
Following de-duplication 887 articles were identified. After screening titles and abstracts, 710 articles were excluded, leaving 177 full text articles. Of these, 95 were excluded, yielding 82 studies. A further 14 studies were identified in the literature after cross checking, totalling 96 analysed studies. 25 relevant in-hospital clinical decision tools were identified, encompassing 67 different clinical criteria, which grouped into 18 categories.

Conclusion:
Factors that should be considered for use in a clinical decision tool designed to support paramedics in the assessment and management of children with head injury, are: signs of skull fracture; a large, boggy or non-frontal scalp haematoma neurological deficit; Glasgow Coma Scale score less than 15; prolonged or worsening headache; prolonged loss of consciousness; post traumatic seizure; amnesia in older children; non-accidental injury; drug or alcohol use; less than one year old. Clinical criteria that require further investigation include mechanism of injury; clotting impairment/anticoagulation; vertigo; length of time of unconsciousness; number of vomits.

Strengths and limitations of the study:

- The review is highly inclusive, with a range of global study settings, including qualitative, quantitative, and mixed methods research.
- The review provided an opportunity to complete a detailed and contemporary search of all available sources and identified 25 in-hospital tools, compared to 14 in a previous systematic review.
- The role of mapping reviews is to provide a descriptive account of the published literature; therefore, this should be considered when assessing the findings of the overall evidence synthesis.
- A direct comparison of the clinical decision support tools is not possible because they address different questions, age groups, injury severities, and outcomes.

INTRODUCTION:

Healthcare systems internationally are under substantial pressure due to rising patient demand. A leading priority is to reduce the number of patients who are unnecessarily conveyed to an Emergency Department (ED) by ambulance [1]. Children frequently use the ambulance service and ED, and childhood head injury is common; approximately 800,000 head-injured children attend EDs each year in the UK [2]. Very few children with head injury who are conveyed to the ED by ambulance need specialist treatment, and the vast majority could be managed safely at scene.

For peer review only - http://bmjopen.bmj.com/site/about/guidelines.xhtml

Despite this, a national overview of head injury in children found that one-third of children in hospital with head injury were transported by ambulance, and 74% of those conveyed were non-serious, requiring no intervention [3].

Head injury guidance produced by the National Institute for Health and Care Excellence (NICE) [4] is of limited utility to paramedics for reasons including: a lack of validation in pre-hospital care; reference to patient observation over time which is not always possible; challenges in the assessment of amnesia in younger children; limited research evidence on which to base recommendations relating to ambulance non-conveyance [4].

There are several existing validated hospital-based decision support tools that clinicians use when a child presents to the ED with a minor head injury, and studies have demonstrated their effectiveness in aiding decision-making for both immediate discharge and computed tomography (CT) scanning [5] [6]. Currently, there are no pre-hospital decision tools designed to support paramedics in reducing unnecessary conveyance to the ED for head-injured children. None of the existing hospital-based tools can be implemented directly in pre-hospital care, since they are designed to support decision making on performing CT scanning, rather than ED conveyance.

AIM:

To systematically identify and examine clinical criteria within existing hospital-based clinical decision support tools for children with minor head injury, with a focus on determining their potential to aid decision making regarding hospital conveyance by paramedics.

METHODS:

PATIENT AND PUBLIC INVOLVEMENT:

Patients and public have been involved throughout this research. Both a Young Persons Advisory Group (YPAG) (children aged 9-16 years) and Parent Advisory Group (PAG) have supported this study and confirmed its importance for children, parents, and the wider health and care system.

Introduction:

Systematic mapping aims to collate, explain, and catalogue available evidence (primary and secondary) relating to a topic of interest, to identify a focus for more specific investigation [7]. It is a review methodology that is often used in healthcare research that aims to detail and categorise literature on a particular topic, with the intention of developing this into more comprehensive work [8]. This systematic mapping review was conducted in accordance with published methods [9] and is reported in line with the PRISMA statement [10]. It has not been registered with Prospero as it is a mapping review.

Patients and public have been involved throughout this research. Both a Young Persons Advisory Group (YPAG) (children aged 9-16 years) and Parent Advisory Group (PAG) have supported this study and confirmed its importance for children, parents, and the wider health and care system.

Search strategy:

Searches were conducted in the following databases, for articles published between January 1980 and August 2022 to ensure that all relevant contemporary research was included: MEDLINE, EMBASE, PsycINFO, CINAHL and AMED **[supplementary material search strategy].** A Google Scholar and a Web of Science search were undertaken to identify reports or proceedings not indexed in the

For peer review only - http://bmjopen.bmj.com/site/about/guidelines.xhtml

above. A literature advisory group, including experts in the field, was consulted to identify relevant grey literature and unpublished reports. Furthermore, to find additional published, unpublished, and ongoing studies, the reference lists of all relevant studies were examined.

Search terms were developed iteratively by discussion among the research team, patient and public representatives and a librarian, seeking a balance between comprehensiveness and focus. A combination of MeSH terms and synonym text-strings/phrases were used in the search strategy and were combined using Boolean operators. Updated searches were re-run before the final analysis. The search terms used can be seen in Table 1.

The search was limited to English language studies due to resource restrictions and the cost of translation, and only articles with full texts available were included. Studies that may have been relevant but did not include or refer to a clinical decision support tool for the assessment and management of head-injured children, were excluded; however, there were no restrictions on the types of study included. Eligibility criteria are presented in full in Table 2.

Table 1. Search terms:

Clinical decision support tool	Head injury	Children	Hospital
Clinical decision tool	Minor head injury	Child	ED
Clinical decision rule	TBI (Traumatic brain injury)	Pediatric	Emergency Department
Diagnostic accuracy tool	Head trauma	Paediatric	A+E
Triage tool	Head wound	Baby	Accident and Emergency
Hospital-based tool	Intracranial injury	Babies	Emergency room
CDRs	CiTBI (clinical important traumatic brain injury)	Infant	Trauma centre
Intervention	ICI (intracranial injury)	Schoolchild	
Clinical decision score	CSII (clinically significant intracranial injury)	Adolescent	
Paediatric head injury predictive tool	Trivial head injury	Teenager	
Clinical Prediction Rule		Young person	
Clinical Prediction Tool			
Clinical decision support tool (CDST)			

Table 2. Inclusion and exclusion criteria:

Inclusion Criteria	Exclusion Criteria
Studies in the English Language	Non- English language
Quantitative, qualitative, mixed methods research, systematic reviews	
Children (under 19 years of age)	Adult population (19 years or older)

For peer review only - http://bmjopen.bmj.com/site/about/guidelines.xhtml

For peer review only

Date of publication 1980-present	
Any study that includes a clinical decision support tool for assessing and managing children with head injury	Any study that did not include a clinical decision support tool for the assessment and management of head-injured children
International as long as in the English language	

Extracting, Coding, and Synthesising the Data:

Titles and abstracts were double screened for relevance and assessed for eligibility using Covidence software [11], following which full texts of included articles underwent review for eligibility. Any disagreements were resolved through discussion with a senior researcher. Reasons for exclusion were documented, a topic expert reviewed the final set of included papers to ensure relevant studies and tools had not been overlooked. Figure 1 shows the PRISMA flow diagram [10].

Data extraction included systematically identifying the title of each tool, the aim, derivation reference, supporting literature, conclusion, and key points relating to the content and format of a clinical decision support tool for paramedic use. An inductive coding frame was developed to map emerging concepts, which involved deriving codes from the data. Key messages/concepts in relation to predicting conveyance from all studies were extracted from the methods, results, and conclusion sections. This resulted in a visual synthesis and classification of available studies and tools. The data extraction tool was piloted using two included studies, and data were extracted by hand and cross-checked by another researcher.

Clinical criteria used in decision making were extracted from all tools; a summary map was produced providing a visual display of clinical criteria included in each tool. After independently producing pilot categories based on a sample of clinical decision support tools, the research team formed consensus on categories. The frequency of each clinical criterion was tabulated, and performance accuracy was recorded where reported. A narrative synthesis was subsequently developed using this framework.

Assessment of Quality:

No formal assessment of quality assessment was undertaken, as this is not required in mapping reviews [12]. Data extraction in mapping reviews describes the studies and their main methods but does not attempt a full data extraction and quality assessment [12].

<u>RESULTS:</u>

A total of 887 articles were identified after duplicates were removed. After screening titles and abstracts, 710 articles were excluded, which left 177 full text articles to be assessed for eligibility. Of these, 95 were excluded for reasons including: adult population only; not including a clinical decision support tool; not in the English language; and full text not available. Three articles were not included, as they were conference abstracts. Fourteen additional studies were identified through citation searching, resulting in 96 included studies (Figure 1).

[Figure 1. PRISMA flow diagram]

In total, 25 relevant in-hospital clinical decision support tools for the assessment and management of children with head injury were identified, which can be seen in Table 3.

Table 3. Hospital based clinical decision support tools for the assessment and management of children with head injury:

For peer review only - http://bmjopen.bmj.com/site/about/guidelines.xhtml

CDST	Full title	Reference
PECARN	Paediatric Emergency Care Applied Research Network rule.	[6] Kuppermann, N., *et al.* (2009) Identification of children at very low risk of clinically important brain injuries after head trauma: A prospective cohort study. *The Lancet,* 374(9696), pp. 1160-1170.
CHALICE	Children's Head injury Algorithm for the prediction of Important Clinical Events Rule.	[13] Dunning, J., Daly, J., Lomas, J., Lecky, F., Batchelor, J. and Mackway-Jones (2006). Derivation of the children's head injury algorithm for the prediction of important clinical events decision rule for head injury in children. *Archives of Disease in Childhood,* 91(11), pp. 885-91.
CATCH	Canadian Assessment of Tomography for Childhood Head injury Rule.	[14] Osmond, M.H. (2010) CATCH: a clinical decision rule for the use of computed tomography in children with minor head injury, *Canadian Medical Association Journal,* 182(4).
NEXUS II	National Emergency X-Ray Utilization Study.	[15] Oman, J. A., Cooper, R. J., Holmes, J. F., Viccellio, P., Nyce, A., Ross, S. E., ... & Mower, W. R. (2006). Predictive performance of a decision rule to predict need for computed tomography among children with blunt head trauma. *Pediatrics,* 117(2), pp. 238-246.
HIDATq	Head Injury Discharge At Triage questionnaire.	[5] Aldridge, P., Castle, H., Phillips, C., Russel, E., Guerrero-Luduena, R. and Rout, R. (2020). Head home: a prospective cohort study of a nurse led paediatric head injury clinical decision tool at a district general hospital, Emergency Medical Journal, 37(11), pp. 680-685.
PALCHAK	UC Davis Rule for Paediatric Head Trauma.	[16] Palchak, M.J *et al.* (2003). A decision rule for identifying children at low risk for brain injuries after blunt head trauma, *Annals of Emergency Medicine*, 42(4), pp. 492-506
HAYDEL	New Orleans/Charity Head Trauma/Injury Rule.	[17] Haydel, M.J. and Shembekar, A. D. (2003) Prediction of intracranial injury in children aged five years and older with loss of consciousness after minor head injury due to nontrivial mechanisms, *Annals of Emergency Medicine,* 42(4).
ATABAKI	No title	[18] Atabaki, S. M., Stiell, I. G., Bazarian, J. J., Sadow, K. E., Vu, T. T., Camarca, M. A., ... & Chamberlain, J. M. (2008). A clinical decision rule for cranial computed tomography in minor pediatric head trauma. *Archives of pediatrics and adolescent medicine,* 162(5), pp. 439-445
GREENES	No title	[19] Greenes, D. S., & Schutzman, S. A. (1999). Clinical indicators of intracranial injury in head-injured infants. *Pediatrics,* 104(4), pp. 861-867.
KLEMETTI	No title	[20] Klemetti, S., Uhari, M., Pokka, T., & Rantala, H. (2009). Evaluation of decision rules for identifying serious consequences of traumatic head injuries in pediatric patients. Pediatric emergency care, 25(12), 811-815
QUAYLE	No title	[21] Quayle, K. S., Jaffe, D. M., Kuppermann, N., Kaufman, B. A., Lee, B. C., Park, T. S., & McAlister, W. H. (1997). Diagnostic testing for acute head injury in children: when are head computed tomography and skull radiographs indicated? *Pediatrics,* 99(5).
DIETRICH	No title	[22] Dietrich, A. M., Bowman, M. J., Ginn-Pease, M. E., Kosnik, E., & King, D. R. (1993). Pediatric head injuries: can clinical factors reliably predict an abnormality on computed tomography? *Annals of emergency medicine, 22*(10), pp. 1535-1540.
GUZEL	No title	[23] Güzel, A., Hiçdönmez, T., Temizöz, O., Aksu, B., Aylanç, H., & Karasalihoglu, S. (2009). Indications for brain computed tomography and hospital admission in pediatric patients with minor head injury: how much can we rely upon clinical findings?. *Pediatric neurosurgery,* 45(4), pp. 262-270

For peer review only - http://bmjopen.bmj.com/site/about/guidelines.xhtml

PredAHT	Predicting Abusive Head Trauma	[24] Cowley, Laura E *et al.* (2018) Acceptability of the Predicting Abusive Head Trauma (PredAHT) clinical prediction tool: A qualitative study with child protection professionals, *Child Abuse & Neglect* 81, pp. 192-205.
CHIDA	Children's Intracranial Injury Decision Aid.	[25] Neumayer, Katie *et al.* (2020) Validation of the "CHIIDA" and application for PICU triage in children with complicated mild traumatic brain injury. *Journal of pediatric surgery*, 55(7), pp. 1255-1259
CIDSS2	Stands for:<br>Coma scale<br>Intoxication<br>Deficit (neurological)<br>Seizure<br>Skull fracture 2	[26] Greenberg, J., Yan, Y., Carpenter, C., Lumba-Brown, A., Keller, M., Pineda, J., Brownson, R. and Limbrick, D. (2018) Development of the CIDSS$_2$ Score for Children with Mild Head Trauma without Intracranial Injury. *Journal of Neurotrauma,* 35(22), pp. 2699-2707.
PediBIRN	Paediatric Brain Injury Research Network	[27] Pfeiffer, H., Smith, A., Kemp, M., Cowley, E., Cheek, A., Dalziel, S., Borland, S., O'Brien, S., Bonisch, M., Neutze, J., Oakley, E., Crowe, L., Hearps, S., Lyttle, M., Bressan, S. and Babl, F. (2018) External validation of the PediBIRN clinical prediction rule for abusive head trauma. *Paediatrics,* 141(5).
Head CT Choice	Head Computed Tomography Choice	[28] Hess, Erik *et al.* (2014) Effectiveness of the head CT choice decision aid in parents of children with minor head trauma: study protocol for a multicentre randomized trial. *Trials,* 15(1), pp. 253-253
SNC Tool	The Scandinavian Neurotrauma Committee Guidance	[29] Unden, J. *et al.* (2018) External validation of the Scandinavian guidelines for management of minimal, mild and moderate head injuries in children, *BMC Medicine,* 16(176).
BIG-1	Brain Injury Guidelines	[30] Schwartz, Jamie *et al.* (2020)<br>Applying Pediatric Brain Injury Guidelines at a Level I Adult/Pediatric Safety-Net Trauma Center.<br>*The Journal of surgical research,* 255, pp. 106-110
Novel Simplified CDR	Novel Simplified Clinical Decision Rule	[31] Yogo, N., Toida, C., Muguruma, T., Gakumazawa, M., Shinohara, M. and Takeuchi, I. (2021) Simplified Clinical Decision Rule Using Clinically Important Events for Risk Prediction in Pediatric Head Injury: A Retrospective Cohort Study, *Journal of Clinical Medicine,* 10(22).
Head Trauma EBG Algorithm	Head Trauma EBG Algorithm	[32] Brittany M. Stopa, *et al.* (2019) Comparison of minor head trauma management in the emergency departments of a United States and Italian Children's hospital, *Italian Journal of Pediatrics,* 45(24).
Da Dalt	No title	[33] Da Dalt, L. *et al*. (2006) Predictors of intracranial injuries in children after blunt head trauma. *European Journal of Paediatrics,* 165(3), pp. 142–8.
Buchanich	No title	[34] Buchanich, J. M. (2007). A clinical decision-making rule for mild head injury in children less than three years old (Doctoral dissertation, University of Pittsburgh).
KIIDS-TBI-CDS	The Kids Intracranial Injury Decision Support tool for Traumatic Brain Injury	[35] Greenberg *et al*. (2021) Development and external validation of the KIIDS-TBI tool for managing children with mild traumatic brain injury and intracranial injuries. *Academic Emergency Medicine,* 28(12).

Sixty-seven decision-making criteria were extracted across all tools. Several criteria related to the same clinical feature (for example, vomiting), however there were many different cut points (e.g.,

For peer review only - http://bmjopen.bmj.com/site/about/guidelines.xhtml

vomiting once only, vomiting twice, or vomiting three times). These 67 criteria were grouped into 18 clinical categories: vomiting; headache; loss of consciousness (LOC); seizure; amnesia; dizziness/vertigo; clotting impairment/anticoagulated; non-accidental injury; age; severe mechanism of injury; drug/alcohol use; signs of a skull fracture; haematoma; neurological deficit; altered mental status; Glasgow come score (GCS); drowsiness and well with no clinical concern.

Supplementary Table 1 shows which clinical categories are included within each hospital-based tool.

[Supplementary Table 1. Categories in each tool]

For peer review only

For peer review only - http://bmjopen.bmj.com/site/about/guidelines.xhtml

Of these clinical categories, the most frequent were skull fracture, loss of consciousness, and scalp haematoma. The most infrequently used included vertigo, suspicion of non-accidental injury and patient acting normal as per parent. Table 4 tabulates the frequency of each clinical criterion.

[Table 4. Frequency of each clinical category across all the tools].

Categories	CDST	Frequency (n=25)
Signs of any description of skull fracture<br><br>(Depressed, basal skull, palpable)	PECARN 18, PECARN 2, NEXUS 2, HIDATq, PALCHACK, CHALICE, CATCH, GUZEL, BUCHANICH, DA DALT, KLEMETTI, QUAYLE, ATABAKI, SNC Guideline, BIG-1, Head trauma EBG algorithm, GREENES, CHIDA, CIDSS2, PediBIRN, KIIDS-TBI CDS	**21 (84%)**
LOC	PECARN 18, PECARN 2, CHALICE, CATCH, NEXUS 2, ATABAKI, KLEMETTI, QUAYLE, HIDATq, GUZEL, DIETRICH, DA DALT, BUCHANICH, SNC Guideline, BIG-1, Novel simplified CDR, Head trauma EBG algorithm	**17 (68%)**
Scalp haematoma/trauma	PECARN 2, CHALICE, PALCHAK, KLEMETTI, NEXUS 2, GUZEL, HAYDEL, GREENES, CATCH, HIDATq, BUCHANICH, ATABAKI, SNC Guideline, Head trauma EBG algorithm, PredAHT, PediBIRN, KIIDS-TBI CDS	**17 (68%)**
Neurological deficit	CHALICE, NEXUS 2, HIDATq, GUZEL, DIETRICH, DA DALT, BUCHANICH, KLEMETTI, ATABAKI, QUAYLE, Head CT Choice, SNC Guidelines, BIG-1, Head trauma EBG algorithm, CIDSS2	**15 (60%)**
Behaviour change/ altered mental status	PECARN 18, PECARN 2, CATCH, HIDATq, PALCHAK, ATABAKI, BUCHANICH, DA DALT, KLEMETI, QUAYLE, NEXUS 2, Head CT Choice, SNC Guidelines, Novel simplified CDR, Head trauma EBG algorithm	**15 (60%)**
GCS	PECARN 18, PECARN 2, CHALICE, NEXUS 2, CATCH, HIDATq, ATABAKI, CHIDA, CIDSS2, Head CT Choice, SNC	**15 (60%)**

For peer review only - http://bmjopen.bmj.com/site/about/guidelines.xhtml

	Guideline, Novel simplified CDR, Head trauma EBG algorithm, DA DALT, KIIDS-TBI CDT	
Vomiting	CHALICE, NEXUS 2, PECARN 18, PALCHACK, HIDATq, HAYDEL, DIETRICH, BUCHANICH, DA DALT, Head CT Choice, SNC Guidelines, Head trauma EBG algorithm	**12 (48%)**
Headache	PECARN 18, CATCH, PALCHAK, GUZEL, DIETRICH, HAYDEL, ATABAKI, BUCHANICH, DA DALT, Head CT Choice, SNC Guidelines, Novel simplified CDR	**12 (48%)**
Seizure	CHALICE, HIDATq, HAYDEL, ATABAKI, QUAYLE, DIETRICH, GUZEL, PredAHT, CIDSS2, SNC Guideline, Novel simplified CDR, DA DALT.	**12 (48%)**
Severe mechanism of injury	PECARN 18, PECARN 2, GUZEL CHALICE, CATCH, HIDATq, ATABAKI, Novel simplified CDR, Head trauma EBG algorithm	**9 (36%)**
Drowsiness	CHALICE, HIDATq, DA DALT, PECARN 18, PECARN 2, NEXUS 2, ATABAKI, Head CT Choice	**8 (32%)**
Amnesia	CHALICE, CATCH, NEXUS 2, HIDATq, HAYDEL, ATABAKI, GUZEL, DA DALT	**8 (32%)**
Drug or alcohol use	HIDATq, HAYDEL, ATABAKI, CIDSS2, BIG-1	**5 (20%)**
Age	PECARN, HIDATq, ATABAKI, HAYDEL	**4 (16%)**
Clotting impairment/anticoagulated	NEXUS 2, HIDATq, SNC Guideline	**3 (12%)**
Dizziness/vertigo	ATABAKI, KLEMETI	**2 (8%)**
Non accidental injury	CHALICE, HIDATq,	**2 (8%)**
Patient alert and well with no clinical concern/acting normal as per parent	PECARN 2, HIDATq	**2 (8%)**

The mix of clinical criteria used, and their outcomes were similar but not identical for any of the tools. In addition, the derivation studies used different populations and sample sizes and therefore the performance accuracy of the tools relating to the potential to correctly rule out clinically significant brain injury in head-injured children were variable (Supplementary Table 2).

For peer review only - http://bmjopen.bmj.com/site/about/guidelines.xhtml

[Supplementary Table 2. Performance accuracy of each tool].

Most tools displayed high sensitivities (>90%), while specificities ranged from 15 to 87%. PECARN was the most widely externally validated, with positive conclusions drawn from research examining post-implementation of the tool [36]. However, PECARN results in a considerable number of normal scans being performed to identify one intracranial injury [37]. The HIDATq tool was designed for nurses to use in triage to safely discharge children with head injury before admission, which more closely resembles the aim of a pre-hospital tool designed to inform which children do not need to be conveyed to hospital. A narrative synthesis is presented below.

Signs of skull fracture:

This was the most frequently used clinical decision-making criterion (21/25) (84%). Variability of terms included: open or depressed skull fracture, basal skull fracture, tense fontanelle, palpable and unclear skull fracture. The only tools that do not include signs of skull fracture are HAYDEL, DIETRICH, PredAHT and Novel Simplified CDR. All three of PECARN, CHALICE and CATCH (the three most widely validated tools) included signs of skull fracture. PredAHT does not include signs of skull fracture despite the aim of this tool being to identify abusive head trauma. In contrast, PediBIRN which aims to identify children with abusive head trauma does include signs of skull fracture as a clinical predictor [38]. The KIIDS-TBI CDS Tool concluded that a depressed skull fracture was a major risk factor for significant brain injury [35]. Babl, et al. (2014) [39] and Da Dalt et al. (2006) [33] found that a basal, open or depressed skull fracture was most likely to trigger a CT response out of any other clinical criteria. Similarly, Quayle et al. (1997) [21] concluded that the relative risk for intracranial injury is increased almost fourfold in the presence of a skull fracture, although the absence of skull fracture does not rule out intracranial injury. The CIDSS2 score [26], a tool to help physicians to identify the minority of children with minor traumatic brain injury at increased risk for extended inpatient stay, uses a scoring system; all clinical criteria score 1, apart from signs of skull fracture which scores 2, suggesting it is deemed the most significant clinical predictor of significant brain injury. Many of the tools, such as NEXUS 2, describe signs and symptoms of a skull fracture, rather than explicitly stating 'signs of a skull fracture'. For example: periorbital or periauricular ecchymoses, haemotympanum, and clear fluid from ears or nose [40].

Scalp haematoma:

Scalp haematoma is used in 17 of the tools identified. However, many specify 'non-frontal' haematomas only, such as the NEXUS 2 tool [40]. Others (GREENES) support inclusion of non-frontal haematomas only; the authors found that non-frontal scalp haematomas had the strongest association with presence of intracranial haemorrhage [19]. The authors of PALCHAK concluded a scalp haematoma is more significant in children under 2 years [16]. The HIDATq tool does not stipulate location of haematoma, however the authors do specify a size (over 5cm) [5]. Similarly, CHALICE only specifies size, which is also over 5cm, but this is for under 1-year olds only [13]. PECARN clearly specifies 'temporal, occipital or parietal haematoma', and this is for the under 2-year-old tool [6]. The CATCH tool includes 'large boggy scalp haematoma', however it is unclear what constitutes 'large' as it does not specify what size or the location. Most excluded haematomas to the face.

Loss of consciousness:

Loss of consciousness (LOC) has different cut points across the identified clinical decision tools, including 'any loss of consciousness', 'loss of consciousness for over 5 seconds' and 'loss of consciousness for over 5 minutes'. It is the second most frequently used clinical predictor, appearing

For peer review only - http://bmjopen.bmj.com/site/about/guidelines.xhtml

in 17 of the 25 clinical decision tools. The authors of PALCHAK [16] found that the risk of a traumatic brain injury was higher in those with a history of loss of consciousness. However, in those children with an isolated loss of consciousness there were no traumatic brain injuries. This is recognised in Kupperman et als. (2009) derivation study for PECARN [6]; the risk of clinically important traumatic brain injury was substantially lower than 1% in children with isolated loss of consciousness. PECARN includes loss of consciousness for over 5 seconds in the under 2 years of age cohort and loss of consciousness of an unspecified duration for the over 2 cohort. Differing to PECARN, CHALICE uses a duration of over 5 minutes of loss of consciousness in any child under 16 years of age, and HIDATq does not specify a duration of loss of consciousness.

Vomiting:

This clinical criterion is a predictor in 12/25 tools, with four varying descriptors including 'any post injury vomiting', 'over 1 vomit', 'over 2 vomits' or 'over 3 vomits'. PECARN includes 'any vomiting' for the over 2-year-old cohort, as does the HIDATq tool, whereas CHALICE takes a different, less risk averse approach including '3 or more' [13]. Both CHALICE and PECARN have a high sensitivity and acceptable specificity for recognising clinically important traumatic brain injury, suggesting that both tools are appropriate for assessing mild head injury in children in the ED, despite the varying descriptions of vomiting. NEXUS 2 includes 'over 1 vomit' and CATCH stipulates 'over 2 vomits', but as eligibility criterion.

Neurological deficit:

For the purposes of this narrative description, neurological deficit will also include focal neurology, Glasgow Coma Scale score (GCS), altered mental status, behavioural change, drowsiness, and vertigo.

The research evidence that underpinned the DA DALT tool concluded that neurological deficit is one of the predictors that significantly increases the likelihood of intracranial injury [33]. Similarly, the CIDSS2 score concluded that neurological deficit was amongst the most important predictors for identifying whether paediatric patients should be admitted to hospital with a head injury [26]. There are varying descriptions to define a neurological deficit across the 25 tools, and there is some cross over with altered mental status/behavioural change, drowsiness, and GCS. Some terms include vision changes, gait changes, repetitive questioning/speech, delayed response to external stimuli, sensory deficit, disorientation or just stating 'neurological deficit'. Authors of the SNC guidelines suggest that the clinical criteria 'altered mental status' is too complicated to use effectively, with potential to lead to unacceptable increases in CT rates [29]. Both PECARN (under 2) and HIDATq include 'patient well with no clinical concern/acting normal as per parent', which suggests no neurological deficit.

Many tools opt to use 'abnormal mental status' with varying descriptors, such as confusion, somnolence, repetitive or slow to respond to verbal communication. A GCS under 15 is associated with the presence of intracranial acute pathologies in young children with minor blunt head trauma [41]. GCS is a clinical criterion in 15 of the 25 identified tools, including PECARN, CHALICE and CATCH.

KLEMETTI is one of the only tools to include 'vertigo' as a clinical criterion; the authors found this variable was amongst one of the most significant to predict severely complicated head trauma in their multivariate logistic regression analysis [20]. ATABAKI is the only other tool to include vertigo as a clinical criterion, however both these tools have low specificity and were not evaluated in a published validation study after development.

For peer review only - http://bmjopen.bmj.com/site/about/guidelines.xhtml

Headache:

Headache is a clinical predictor in only half of the 25 in-hospital tools. This may be because it is challenging to assess for a headache in younger children; they may express pain differently with signs such as irritability and persistent crying [42]. The authors of the BUCHANIC tool use headache as a clinical predictor, despite an inclusion criterion of aged under 3 years only, however, this tool is not validated. PECARN includes headache for the aged 2- 18 years cohort, but not the aged under 2 years. For children who can verbalise or show that they have a headache, it is important to consider severity and timing of the headache. Some tools specify 'severe headache', 'prolonged headache' or 'worsening headache'. Worsening headache is associated with intracranial injury, being deemed a 'high risk' criterion in CATCH [14]. The HIDATq tool does not include headache as a clinical predictor, despite presenting the tool as 'ultra-safe', although it does include 'irritability' which could indicate pain in a younger child.

Seizure:

Just under half of the 25 in-hospital tools included seizure as a clinical predictor despite a post traumatic seizure being a high determinant for an intracranial injury [43]. The CIDSS2 score found seizure to be one of the most significant criteria to identify whether patients should be admitted with head injury. PECARN is the most widely validated and implemented tool, however it does not include seizure as a specific clinical predictor, compared with CHALICE and HIDATq, which do.

Severe mechanism of injury:

Nine of the 25 identified clinical decision support tools include 'severe or dangerous mechanism of injury' as a clinical predictor. The description of what constitutes a 'severe' mechanism of injury varies across the tools, from different levels of fall from height, road traffic collisions, to specific considerations such as a bicyclist not wearing a helmet who is struck by a car, however; fall from height is the most common mechanism of injury in children. The SNC guideline [29] does not include high-energy trauma mechanism as a strict risk factor, as these patients are relatively uncommon in Scandinavia and are managed according to separate clinical trauma protocols. The authors and creators of the SNC guidelines judged 'mechanism of injury' as a complicated clinical predictor to use, due to having a specific definition and often including assessment of fall height, vehicle speed and number of stairs [29]. Similarly, the Novel Simplified Tool [31] suggests that mechanism of injury is not considered reliable, and that it may be an overestimation to emphasise the injury mechanism as a predictor. The aim of this clinical decision tool is to provide a simplified version of PECARN, CATCH and CHALICE (all of which include varying descriptors for mechanism of injury), so that it can be easily and quickly used for paediatric patients in busy emergency settings. Therefore, one distinguishing feature is that that this tool includes only one predictor for mechanism of injury, however; the Novel CDR misclassified eight patients as low risk.

Amnesia:

The authors of PALCHAK conclude that elimination of amnesia as a clinical predictor may decrease unnecessary CT scanning [16]. However, amnesia is a clinical predictor in eight of the 25 hospital-based tools identified within this review. It is not possible to assess amnesia in non-verbal children and it is difficult to assess in young children [33] which is perhaps why PECARN does not include amnesia as a clinical predictor, however all of CHALICE, CATCH, NEXUS 2 and HIDATq do. One study adapted the PECARN tool by introducing amnesia alongside other clinical predictors [44]. The authors concluded that the adapted PECARN rule was successfully implemented in an Italian

For peer review only - http://bmjopen.bmj.com/site/about/guidelines.xhtml

Paediatric ED; its use determined a low CT scan rate that was unchanged compared to previous clinical practice and showed an optimal safety and high efficacy profile.

Non-accidental injury:

Non-accidental injury is only included as a clinical predictor in two of the in-hospital clinical decision support tools (HIDATq and CHALICE), although some tools include non-accidental injury as an exclusion criterion, such as the Head Injury EBG algorithm and CATCH. Despite this, non-accidental injury is one of the biggest predictors of brain injury, particularly in children under the age of 2 years [45]. PECARN is not especially sensitive for identifying physically abused children [46]. There is no gold standard diagnostic test to identify abusive head trauma though PredAHT and PediBIRN are specifically designed tools that can help clinicians to recognise cases of abusive head trauma [46].

Clotting impairment:

Only three of the 25 in-hospital tools include clotting/impairment as a clinical predictor (HIDATq, the SNC guideline and NEXUS 2), since it is uncommon for a child to be on these types of drugs or have bleeding diatheses. The HIDATq tool is derived using the NICE head injury guidance and is designed to be 'ultra-safe' since the aim of the tool is to discharge children with head injury at triage, and therefore includes 'anticoagulant use/clotting impairment. None of the three most widely validated tools (PECARN, CATCH and CHALICE) include 'anticoagulant use/clotting disorder' as a clinical predictor, because it was too rare to be of use.

Drug and alcohol use:

It is well known that alcohol increases the risk of suffering a head injury due to its effects on balance and coordination [47]. Children with suspected drug or alcohol intoxication may be more difficult to assess; clinicians should assume conscious level and amnesia relates to injury and have a lower threshold for referral and neuroimaging, since blunt head injuries in intoxicated patients is associated with a higher mortality [48]. Alcohol can also lead to headaches, nausea, and irritability, making examination challenging. Five of the identified clinical decision support tools included 'drug/alcohol use' (HIDATq, HAYDEL, ATABAKI, CIDSS2, BIG-1). None of the most validated tools, PECARN, CHALICE, or CATCH include this as a clinical predictor.

Age:

All the identified clinical decision support tools in this review specify an age in which the tool can be used, e.g., under 18 years, and this varies from aged under 2-years to under 21-year-olds. However, only two tools exclude younger age groups as a clinical predictor (HIDATq excludes under 1s and ATABAKI excludes under 2's). HAYDEL is only for five–17-year-olds and PECARN has a separate tool for under 2's, with different questions, more specific to that age group [6]. BUCHANIC, PredAHT and PediBIRN are designed specifically for under 3's, and GREENES is for under 2's only, however despite these tools aiming at the same age group, their clinical predictors still vary considerably. The authors of GREENES found that infants are at greater risk of intracranial hypertension due to their open fontanels and that under 1's with intracranial injuries are frequently asymptomatic, making management inherently difficult for clinicians [19]. It may be for this reason (as well as the increased likelihood of non-accidental injury) that children under 3 months were significantly more likely to receive a CT scan using the PECARN tool.

DISCUSSION:

For peer review only - http://bmjopen.bmj.com/site/about/guidelines.xhtml

This systematic mapping review identified and examined clinical criteria within existing hospital-based clinical decision support tools for children with minor head injury to determine their utility in paramedic practice, to inform the need for hospital conveyance. There are additional factors which require consideration in operationalising a tool for pre-hospital use, including the purpose of existing clinical decision rules, and additional relevant clinical factors.

Challenges of selecting a tool for use in pre-hospital care:

None of the existing hospital-based clinical decision support tools can be translated directly into paramedic practice. No single hospital based clinical decision support tool included all 18 clinical criteria, and there are potentially additional criteria that are not included in any of the tools that may be useful specifically in pre-hospital care; however, this needs to be explored in subsequent work. Most hospital based clinical decision support tools are designed to identify children who need a CT scan; although, this is not actually the clinical end point of interest. The reason for doing a CT scan is to seek intracranial injury that requires neurosurgery. Arguably, if a child receives no active treatment, then a CT scan is unnecessary, even if it does show intracranial injury [49] especially as paediatric CT scanning results in a significantly increased lifetime risk of radiation induced fatal malignancy [50]. The aim of a pre-hospital clinical decision support tool designed for use by paramedics (following additional research) will likely be to identify head-injured children who can safely be left at scene, and *do not* need conveyance. Therefore, the tool will "rule-out" serious head injury and be highly sensitive, since if all answers are negative in a highly sensitive tool, the tool can be used to rule out clinically significant brain injury. This is accepting that there are complicating issues specific to pre-hospital care such as local ambulance policy, time of day, time since injury, complex wound closure, safeguarding, parental concern, and parental capability, that need to be considered and may warrant conveyance, despite there being minimal concern of a clinically significant head injury [51].

All the existing hospital-based tools address diverse questions, age groups, injury severities, and outcomes and have been derived, evaluated, and validated in a different way. In comparison to CHALICE, CATCH and other hospital-based tools, PECARN identifies children with a very low risk of brain injury who *do not* need a scan, which is an important concept, because it considers who a clinician *does not* need to scan, rather than who they *do* [6]. PECARN was derived using two large multicentre cohorts with over 40,000 children and is extremely sensitive, particularly for infants. A tool designed for use by paramedics in the pre-hospital setting would likely aim to follow this approach and identify children who can safely be left at scene and *do not* need conveyance, due to sustaining a trivial or minor head injury that is very low risk of clinically significant brain injury, similarly to Kupperman's PECARN [6], though this needs further consideration and study. The role of ambulance paramedics includes the ability to make autonomous decisions on the safe discharge of patients at scene to prevent unnecessary conveyance to the ED. This is similar to in-hospital clinicians whose role it is to discharge patients from the ED when it is safe to do so. Therefore, the in-hospital clinical decision support tools for the assessment and management of children with head injury will share many similarities, particularly if the tools are being used in triage. PECARN is also the only tool that considers the age of the child from the beginning and thereafter asks different questions relevant to the child's age [6]. Paramedics have reported difficulty using the NICE head injury guidance [4] for this reason, as they could not ask younger children about amnesia, headache, and alcohol use [51].

Paramedic use of clinical decision support tools in current practice:

Previous research demonstrates that paramedics find clinical decision tools useful in identifying alternative patient pathways for other patient groups, such as older adults following a fall [52]. Clinical decision tools are not a new concept and have been at the forefront of digital health solutions for

For peer review only - http://bmjopen.bmj.com/site/about/guidelines.xhtml

more than twelve years [53]. In pre-hospital care, they are used by paramedics to manage risk and support referral to alternative care pathways and community-based care [54]. Additionally, the use of clinical decision tools in the pre-hospital setting by paramedics has been shown to significantly reduce the opportunity for human error, improve diagnostic accuracy, improve optimisation of resources, improve compliance with guidelines and improve overall quality and safety of care for patients [55] [56]. One systematic review of randomised controlled trials (RCT) compared clinical decision tools to usual care in the pre-hospital setting and concluded that there are too few RCTs to draw any firm conclusions in pre-hospital care, although there is a possibility that clinical decision tools increase diagnostic accuracy [57]. A more recent cluster RCT investigating the effectiveness of a clinical decision tool to increase the number of elderly fallers referred to a community falls team rather than ED, found evidence that paramedics use clinical decision tools to justify current practice rather than to inform decisions. The study found that paramedics decided to leave patients at home first, and then used the clinical decision tool to justify their decision. The tool was found to be cost effective and successful at changing paramedic practice [58].

__What clinical criteria should be included:__

All 18 clinical criteria identified in this systematic mapping review are important to consider for a pre-hospital tool designed to support paramedics in their assessment and management of children with head injury, however some may be difficult to operationalise in the pre-hospital setting. In addition, the hospital-based tools have varying cut off points for different clinical criteria and which descriptor a pre-hospital tool should use requires further consideration. It is important to acknowledge that all clinical decision tools should be used alongside clinical judgment. Whilst this review is intended to support the process of developing a clinical tool to support paramedic decision making at scene, it is only an initial step, and it is recognised that considerably more research is required. It would only be reasonable to adapt in-hospital tools for pre-hospital use if it could be demonstrated that the tool was applied with similar reliability and accuracy to that which is currently achieved in hospital practice.

Certainty of use:

It is clear from the evidence that some clinical predictors are significant for identifying a potentially clinically important traumatic brain injury following a head injury and should be included in a pre-hospital tool, such as a post-traumatic seizure [43]. Signs of a skull fracture was most likely to trigger a CT response over any other clinical criteria, since the risk of intracranial injury is significantly increased in the presence of a skull fracture. This is reflected in the NICE guidance for the management of head injury, which suggests a CT scan should be performed within an hour if there are signs of a skull fracture [4]. The tools indicated that specifically a 'large', or 'non-frontal' (particularly parietal) [19] or 'boggy' haematoma, especially in infants, is more indicative of significant intracranial injury, which is apparent in other research [57]. Under 6-month-olds have a higher likelihood of significant intracranial haemorrhage with haematomas [59]. Greenes and Schutzman (2001) [19] suggest that among asymptomatic head-injured infants, the risk of skull fracture and associated intracranial haemorrhage is correlated with both scalp haematoma size and location. In most ambulance trusts across the United Kingdom, local policy stipulates that any under 2-year-old should be conveyed to the ED, no matter how mild the presenting injury [51]. Abusive head trauma occurs more frequently in under 1's [60]; paramedics are in a unique position to identify suspected non accidental injury since they attend the patient's home environment often in a time of crisis and have a professional duty to protect vulnerable persons [61]. This evidence supports the recommendation of including 'infants' as a clinical predictor for a pre-hospital tool designed to aid decision making regarding conveyance, or to exclude under 1's entirely. Non accidental injury is only a clinical predictor in two in-hospital clinical

For peer review only - http://bmjopen.bmj.com/site/about/guidelines.xhtml

decision tools, however; perhaps this is because it is seen as a "no-brainer" and children were often excluded from the study cohort, as they would always need a scan. A child with suspected non accidental injury should always be conveyed to the ED by paramedics. Abuse tools such as those derived by Pfeiffer [27] and Cowley [24] include certain clinical predictors that can help paramedics in recognising non accidental injury.

Clinical predictors that may need intervention:

The pre-hospital environment is very different to in-hospital and there are some clinical predictors that may warrant conveyance as they may need interventions that cannot be done pre-hospitably, such as observation and complex wound closure. In addition, there are factors that are outside of a paramedic's scope of practice that presents too much risk to discharge at scene, and therefore warrants conveyance.

The number of vomits was variable across the tools; research suggests that children who vomit after a head injury is less significant than in adults and they do not necessarily have a serious brain injury [62]. Other literature supports this, suggesting that clinically important traumatic brain injuries are uncommon in children presenting with head injury with vomiting and a management approach of observation without immediate CT may be appropriate [63]. However, a period of observation is not always practical or possible for paramedics in the pre-hospital setting. This was reported by paramedics in a survey exploring reasons why paramedics convey children with minor head injury to the ED [51]. However, most children with head injury (90%) who are admitted for observation are discharged without any further treatment being given [64]. Notably, *persistent* vomiting post head injury is indicative of a subdural haematoma or skull fracture; Harper *et al.* (2020) [65] supports this, concluding that discreet vomiting over four times was a significant risk factor for intracranial injury. Additionally, vomiting combined with other clinical criteria such as headache and altered mental status, can indicate a more serious head injury [63]. This theme is clear with other clinical predictors such as loss of consciousness; children with minor blunt head trauma presenting to the ED with *isolated* loss of consciousness are at very low risk for clinically significant brain injury and do not routinely require CT scanning [66], however if the loss of consciousness was associated with other clinical predictors, the risk is increased. Any clinical predictor present in isolation is less likely to indicate clinically significant traumatic brain injury than the presence of multiple risk criteria [63]. This is similar for mechanism of injury; evidence implies that mechanism of injury alone is less predictive of an abnormal brain CT, compared with other clinical predictors [67]. Despite this, most paramedics would convey a head-injured child based solely on mechanism, despite them appearing well with no clinical concern [51].

Fowler *et al.* (2018) [68] found that attending to paediatric patients evokes anxiety and discomfort amongst paramedics, which often led to reluctance to initiate treatment and poorer care. This includes unnecessary transport to the ED. Approximately half of paramedic respondents in a survey reported feeling 'not at all confident' with completing a neurological assessment on a child following a head injury [51], despite a focal neurological deficit being a strong indicator of intracranial injury [33]. GCS is inherently difficult to assess in a younger child, however a reduced GCS is a high-risk factor for clinically significant intracranial injury [41].

Clinical predictors that infrequently result in intracranial injury:

There is limited evidence to suggest vertigo as a standalone clinical predictor should be included in a decision tool for conveyance of children with head injury, and this needs further investigation [20]. Additionally, evidence is still conflicting regarding the risk of intracranial haemorrhage in children with

For peer review only - http://bmjopen.bmj.com/site/about/guidelines.xhtml

a clotting impairment/anticoagulation, [69], despite this being a research priority since 2011 [70. Bressan *et al.* (2020) [69] found that although children with a bleeding disorder who have sustained a head injury more often received a CT scan, their risk of intracranial haemorrhage was low. Their findings are echoed in other literature [71]; CTs are obtained twice as often in children with bleeding disorders, although intracranial haemorrhage occurred in only 1.1% of these patients, and they had symptoms that would have prompted the need for a CT anyway. New guidance from NICE [4] advocates a CT head scan within eight hours for children on anticoagulant treatment, or within an hour if they have a bleeding/clotting disorder, independently of other indications. However, only one out of 31 children on anticoagulants in Bressen *et als.* (2020) [69] and Lee *et als*. (2011) [71] study combined, was diagnosed with an intracranial haemorrhage, and that patient very obviously needed a CT from the outset. These clinical criteria need further consideration before being included in a pre-hospital tool for paramedic use.

Research trajectory for a clinical decision tool designed to support paramedics in the assessment and management of children with head injury:

The proposed development of this clinical decision support tool will follow the MRC guidance for complex interventions. The 'development' phase of the 'development-feasibility-evaluation-implementation' process should begin with an investigation and identification of the evidence base through a systematic review. The next stage will involve developing theory using interviews and a Delphi with key stakeholders, with the aim of creating the new tool. Following this, the process and outcomes will be modelled before proceeding to a full-scale evaluation by assessing the acceptability and usability of the new tool by paramedics using simulated scenarios. Further evaluation will involve assessing the validity and test characteristics of the final tool to examine safety, without changing clinical practice. If validity and safety are confirmed then a feasibility study for a subsequent randomised controlled trial to determine whether the introduction of the new support tool reduces conveyance to the ED for children with head injury, whilst maintaining safety and avoiding adverse clinical outcomes. It is acknowledged that even if the tool had very high sensitivity, it would need to be compared to clinical judgement.

LIMITATIONS:

The role of mapping reviews is to provide a descriptive account of the published literature; therefore, this should be considered when assessing the findings of the overall evidence synthesis. A direct comparison of the clinical decision tools is not possible because they address different questions, age groups, injury severities, and outcomes. It is recognised that none of the evidence is from the pre-hospital environment, since there are no existing out-of-hospital clinical decision support tools designed to support the assessment and management children with head injury, hence the need for this research. Therefore, the in-hospital publications of tools considering childhood injury is the only research available to draw conclusions from at this point. Elements of in-hospital research can be attributed to pre-hospital care since there are similarities, however it is acknowledged that further research is required with pre-hospital staff in the pre-hospital environment following this work. Although rigorous methods of identifying all in-hospital tools have been followed, it is possible that some studies have been overlooked. This is particularly the case for international studies since this review included English language articles only.

CONCLUSIONS AND FUTURE RESEARCH:

For peer review only - http://bmjopen.bmj.com/site/about/guidelines.xhtml

Clinical predictors that markedly increase the likelihood of neurosurgical intervention and should be considered for inclusion in a clinical decision support tool for use by paramedics are:: signs of a skull fracture; a large, boggy or non-frontal haematoma (particularly in an infant); persistent discreet episodes of vomiting; a focal neurological deficit; GCS less than 15; a prolonged or worsening headache; a prolonged loss of consciousness; a post traumatic seizure; amnesia in older children; suspicion of non-accidental injury; drug or alcohol use and being under 1 year old. Clinical criteria that require further investigation include mechanism of injury; clotting impairment/anticoagulated; vertigo; length of time of loss of consciousness; number of vomits, and description of what constitutes a neurological deficit in younger children. Any clinical predictor present in isolation is unlikely to indicate clinically significant traumatic brain injury.

There are likely to be additional clinical criteria that are relevant to paramedic assessment and practice, which are not included in any of these tools. Such criteria would need to be identified and explored in future qualitative work involving interviewing paramedics about what influences them when deciding to convey children with minor head injury to the hospital. None of the existing hospital-based clinical decision support tools can be directly implemented into paramedic practice; however, elements from each of the tools can be adapted to create a new tool specific for paramedics in pre-hospital care. Future qualitative research is needed to investigate paramedics' thoughts on a clinical decision tool designed to support them in their assessment and management of head-injured children. Additionally, future research utilising Delphi methods is required to build consensus amongst study matter experts and users to enable the development of a new clinical decision tool to support paramedics in safely assessing and managing children with minor head injury.

CONFLICT OF INTEREST:

None

No financial relationships with any organisations that might have an interest in the submitted work in the previous three years, no other relationships or activities that could appear to have influenced the submitted work.

COPYRIGHT:

The Corresponding Author has the right to grant on behalf of all authors and does grant on behalf of all authors, an exclusive licence (or non-exclusive for government employees) on a worldwide basis to the BMJ Publishing Group Ltd to permit this article (if accepted) to be published in BMJ editions and any other BMJPGL products and sublicenses such use and exploit all subsidiary rights, as set out in our licence

TRANSPARENCY STATEMENT:

This manuscript is an honest, accurate and transparent account of the study being reported. No important aspects of the study have been omitted and any discrepancies from the study as originally planned have been explained.

FUNDING SOURCE:

This work is supported by Health Education England/National Institute for Health Research Integrated Clinical and Practitioner (ICA) Programme's Doctoral Clinical and Practitioner Academic Fellowship (DCAF) Scheme, award number 25215. The views expressed are those of the authors and

For peer review only - http://bmjopen.bmj.com/site/about/guidelines.xhtml

do not necessarily represent those of Health Education England, the National Institute for Health Research or Department of Health.

ETHICS APPROVAL:

Not required

DATA SHARING:

No additional data available

AUTOR CONTRIBUTOR STATEMENT:

JB, SV and ML supervised the work. AP wrote the protocol, conducted the review, and drafted the manuscript with input from JB, SV, ML, JeB and JS. JeB double screened the abstracts and full texts. PS helped to design the search terms and conduct the search. All authors interpreted and analysed the results. All authors discussed the results, contributed to and approved the final manuscript.

REFERENCES:

1. NHS (2019) The NHS Long term Plan. Available at: https://www.longtermplan.nhs.uk/ [Accessed 30th January 2023].
2. Arora, R., White, E.N., Niedbala, D., Ravichandran, Y., Sethuraman, U. and Radovic, N. (2020) Reducing Computed Tomography Scan Utilization for Paediatric Minor Head Injury in the Emergency Department: A Quality Improvement Initiative. *Academic Emergency Medicine,* 28(6), pp. 655-665.
3. Healthcare Quality Improvement Partnership (2015) Traumatic head injury in children and young people: a national overview. Available at: https://www.hqip.org.uk/wp-content/uploads/2018/02/traumatic-head-injury-in-children-and-young-people-a-national-overview.pdf [Accessed 30th January 2023].
4. National Institute for Health and Care Excellence (2023) *Head Injury Management.* Available at: https://cks.nice.org.uk/topics/head-injury/management/ [Accessed 23 May 2023].
5. Aldridge, P., Castle, H., Phillips, C., Russel, E., Guerrero-Luduena, R. and Rout, R. (2020). Head home: a prospective cohort study of a nurse led paediatric head injury clinical decision tool at a district general hospital, Emergency Medical Journal, 37(11), pp. 680-685.
6. Kuppermann, N., *et al.* (2009) Identification of children at very low risk of clinically-important brain injuries after head trauma: A prospective cohort study. *The Lancet,* 374(9696), pp. 1160-1170.
7. Campbell, F., Tricco, A.C., Munn, Z., Pollock, D., Saran, A., Sutton, A., White, H. and Khalil, H. (2023) Mapping reviews, scoping reviews, and evidence and gap maps: the same but different – the big picture review family, *Systematic reviews*, 12(45).
8. Grant, M.J. and Booth, A. (2009) A typology of reviews: an analysis of 14 review types and associated methodologies. *Health Info Library Journal,* 26(91), pp.108-9.
9. Clapton, J., Rutter, D. and Sharif, N. (2009) *Social Care Institute for Excellence (SCIE) systematic mapping guidance.* Available at: https://www.scie.org.uk/publications/researchresources/rr03.pdf [Accessed 30 January 2023].

For peer review only - http://bmjopen.bmj.com/site/about/guidelines.xhtml

10. Page, M.J., Mckenzie, J.E., Bossuyt, P.M., Hoffman, T.C. and Mulrow, C.D. (2020) The PRISMA 2020 statement: an updated guideline for reporting systematic reviews. BMJ, 172(71).
11. Covidence systematic review software, Veritas Health Innovation, Melbourne, Australia. Available at www.covidence.org [Accessed 18th April 2023].
12. Cooper, D. (2016). What is a "mapping study?" *Journal of the Medical Library Association,* 104(1), pp. 76-78.
13. Dunning, J., Daly, J., Lomas, J., Lecky, F., Batchelor, J. and Mackway-Jones (2006). Derivation of the children's head injury algorithm for the prediction of important clinical events decision rule for head injury in children. *Archives of Disease in Childhood,* 91(11), pp. 885-91.
14. Osmond, M.H (2010) CATCH: a clinical decision rule for the use of computed tomography in children with minor head injury, *Canadian Medical Association Journal,* 182(4).
15. Oman, J. A., Cooper, R. J., Holmes, J. F., Viccellio, P., Nyce, A., Ross, S. E., ... & Mower, W. R. (2006). Predictive performance of a decision rule to predict need for computed tomography among children with blunt head trauma. *Pediatrics,* 117(2), pp. 238-246.
16. Palchak, M.J *et al.* (2003). A decision rule for identifying children at low risk for brain injuries after blunt head trauma, *Annals of Emergency Medicine*, 42(4), pp. 492-506
17. Haydel, M.J. and Shembekar, A. D. (2003) Prediction of intracranial injury in children aged five years and older with loss of consciousness after minor head injury due to nontrivial mechanisms, *Annals of Emergency Medicine,* 42(4).
18. Atabaki, S. M., Stiell, I. G., Bazarian, J. J., Sadow, K. E., Vu, T. T., Camarca, M. A., ... & Chamberlain, J. M. (2008). A clinical decision rule for cranial computed tomography in minor pediatric head trauma. *Archives of pediatrics and adolescent medicine,* 162(5), pp. 439-445
19. Greenes, D. and Schutzman, S. (2001) Clinical significance of scalp abnormalities in asymptomatic head-injured infants. *Paediatric Emergency Care,* 17(2), pp. 88-92.
20. Klemetti, S., Uhari, M., Pokka, T., & Rantala, H. (2009). Evaluation of decision rules for identifying serious consequences of traumatic head injuries in pediatric patients. Pediatric emergency care, 25(12), 811-815
21. Quayle, K. S., Jaffe, D. M., Kuppermann, N., Kaufman, B. A., Lee, B. C., Park, T. S., & McAlister, W. H. (1997). Diagnostic testing for acute head injury in children: when are head computed tomography and skull radiographs indicated?. *Pediatrics,* 99(5).
22. Dietrich, A. M., Bowman, M. J., Ginn-Pease, M. E., Kosnik, E., & King, D. R. (1993). Pediatric head injuries: can clinical factors reliably predict an abnormality on computed tomography?. *Annals of emergency medicine, 22*(10), pp. 1535-1540.
23. Güzel, A., Hiçdönmez, T., Temizöz, O., Aksu, B., Aylanç, H., & Karasalihoglu, S. (2009). Indications for brain computed tomography and hospital admission in pediatric patients with minor head injury: how much can we rely upon clinical findings?. *Pediatric neurosurgery,* 45(4), pp. 262-270.
24. Cowley, Laura E *et al.* (2018) Acceptability of the Predicting Abusive Head Trauma (PredAHT) clinical prediction tool: A qualitative study with child protection professionals, *Child Abuse & Neglect* 81():pp. 192-205.
25. Neumayer, Katie *et al.* (2020) Validation of the "CHIIDA" and application for PICU triage in children with complicated mild traumatic brain injury. *Journal of pediatric surgery*, 55(7), pp. 1255-1259
26. Greenberg, J., Yan, Y., Carpenter, C., Lumba-Brown, A., Keller, M., Pineda, J., Brownson, R. and Limbrick, D. (2018) Development of the CIDSS2 Score for Children with Mild Head Trauma without Intracranial Injury. *Journal of Neurotrauma,* 35(22), pp. 2699-2707.

For peer review only - http://bmjopen.bmj.com/site/about/guidelines.xhtml

27. Pfeiffer, H., Smith, A., Kemp, M., Cowley, E., Cheek, A., Dalziel, S., Borland, S., O'Brien, S., Bonisch, M., Neutze, J., Oakley, E., Crowe, L., Hearps, S., Lyttle, M., Bressan, S. and Babl, F. (2018) External validation of the PediBIRN clinical prediction rule for abusive head trauma. *Paediatrics,* 141(5).
28. Hess, Erik *et al.*(2014) Effectiveness of the head CT choice decision aid in parents of children with minor head trauma: study protocol for a multicentre randomized trial. *Trials,* 15(1), pp. 253-253.
29. Unden, J. *et al.* (2018) External validation of the Scandinavian guidelines for management of minimal, mild and moderate head injuries in children, *BMC Medicine,* 16(176).
30. Schwartz, Jamie *et al.* (2020) Applying Pediatric Brain Injury Guidelines at a Level I Adult/Pediatric Safety-Net Trauma Center. *The Journal of surgical research,* 255, pp. 106-110
31. Yogo, N., Toida, C., Muguruma, T., Gakumazawa, M., Shinohara, M. and Takeuchi, I. (2021) Simplified Clinical Decision Rule Using Clinically Important Events for Risk Prediction in Pediatric Head Injury: A Retrospective Cohort Study, *Journal of Clinical Medicine,* 10(22).
32. Brittany M. Stopa*, et al.* (2019) Comparison of minor head trauma management in the emergency departments of a United States and Italian Children's hospital, *Italian Journal of Pediatrics,* 45(24).
33. Da Dalt, L. *et al.*(2006) Predictors of intracranial injuries in children after blunt head trauma. *European Journal of Paediatrics,* 165(3), pp. 142–8.
34. Buchanich, J. M. (2007). A clinical decision-making rule for mild head injury in children less than three years old (Doctoral dissertation, University of Pittsburgh).
35. Greenberg *et al*. (2021) Development and external validation of the KIIDS-TBI tool for managing children with mild traumatic brain injury and intracranial injuries. *Academic Emergency Medicine,* 28(12).
36. Bressan, Silvia *et al.* (2012) Implementation of adapted PECARN decision rule for children with minor head injury in the paediatric emergency department. *Academic emergency medicine: official journal of the Society for Academic Emergency Medicine*, 19(7), pp. 801-807.
37. Babl, F. E., Oakley, E., Dalziel, S.R., Crowe, L., Bressan, S. and Lyttle, M. (2018) Accuracy of clinician practice compared with three head injury decision rules in children: a prospective cohort study, Annals of Emergency Medicine, 71 (6).
38. Pfeiffer, H., Smith, A., Kemp, M., Cowley, E., Cheek, A., Dalziel, S., Borland, S., O'Brien, S., Bonisch, M., Neutze, J., Oakley, E., Crowe, L., Hearps, S., Lyttle, M., Bressan, S. and Babl, F. (2018) External validation of the PediBIRN clinical prediction rule for abusive head trauma. Paediatrics, 141(5).
39. Babl, F.E et al. (2014) Head injury decision rules in children. Lancet, 390, pp. 1488.
40. Gupta, M., William, M., Rodriguez, R. and Hendey, G. (2018) Validation of the Pediatric NEXUS II Head Computed Tomography Decision Instrument for Selective Imaging of Pediatric Patients with Blunt Head Trauma. *Academic Emergency Medicine,* 25(7), pp. 729-737.
41. Pickering, A., Harnan, S., Fitzgerald, P., Pandor, A. and Goodacre, S. (2011) Clinical decision rules for children with minor head injury: a systematic review, Archives of Disease in Childhood, 95(5).
42. Doll, E., Gong, P., Sowell, M. and Evanczyk, L. (2021) Post-traumatic Headache in Children and Adolescents, Current Pain and Headache Reports, 25(51).
43. Fordington, S. and Manford, M. (2020) A review of seizures and epilepsy following traumatic brain injury, Journal of Neurology, 267(10).

For peer review only

44. Bressan, S.B., Romanato, S., Mion, T., Zanconato, S. and Da Dalt, L. (2012) Implementation of adapted PECARN decision rule for children with minor head injury in the paediatric emergency department, Academic Emergency Medicine, 19 (7).
45. Hymel, K.P., Fingarson, A.K., Pierce, M.C., Kaczor, K., Makoroff, K.L. and Wang, M. (2022) External Validation of the PediBIRN Screening Tool for Abusive Head Trauma in Paediatric Emergency Department Settings, Paediatric Emergency Medicine, 38(6).
46. Ide, K., Uematsu, S., Tetsuhara, K., Yoshimura, S. and Kato, T. (2016) External Validation of the PECARN Head Trauma Prediction Rules in Japan, Academic Emergency Medicine, 24(3), pp. 308-314.
47. Weil, Z.M., Corrigan, J.D., Karelina, K. (2018) Alcohol use disorder and traumatic brain injury, Alcohol Research, 39(2), pp. 171-180.
48. Lin, H., Lin, T., Soo, K., Chen, C., Kuo, L., Lin, Y., Lee, W. and Lin, C. (2014) The Effect of Alcohol Intoxication on Mortality of Blunt Head Injury, Biomed Research International.
49. Kambal, M. A., Abou, E.M., Gadi, I, A., Boukai, A. A., Jurayyan, N.A. and Babiker, A.M. (2014). Managing traumatic brain injury in children: When do we need a computed tomography of the head? Sudanese Journal of Paediatrics, 14(1).
50. Meulepas, J.D. et al. (2019) Radiation Exposure from Paediatric CT scans and Subsequent Cancer Risk in the Netherlands. Journal of the National Cancer Institute, 111(3), pp. 256-263.
51. Proctor, A., Voss, S., Lyttle, M.D. and Benger, J. (2023) Assessment and management of children with head injury: practice and opinion, *Journal of Paramedic Practice,* 15(2).
52. Oosterwold, J., Sagel, D., Berben, S, Roodbol, P. and Broekhuis, M. (2018) Factors influencing the decision to convey or not to convey elderly people to the emergency department after emergency ambulance attendance: a systematic mixed studies review. BMJ Open.

53. Mann, D., Hess, R. and Feldstein, A. (2019) Adaptive design of a clinical decision support tool: What the impact on utilization rates means for future CDS research. Digital Health, 5.
54. Porter, A., Dale, J., Foster, T., Logan, P., Wells, B. and Snooks, H. (2018) Implementation and use of computerised clinical decision support (CCDS) in emergency pre-hospital care: a qualitative study of paramedic views and experience using Strong Structuration Theory. BMC, 13(91)
55. Yeats, A. (2018). *Using Clinical Decision Support to Reduce Clinical Risk.* Available at: https://www.oneadvanced.com/news-and-opinion/using-clinical-decision-support-to-reduce-clinical-risk/- [Accessed 05 July 2022].
56. Bashiri, A., Savareh, B.A. and Ghazisaeed, M. (2019) Promotion of prehospital emergency care through clinical decision support systems: opportunities and challenges, Clinical and Experimental Emergency Medicine, 6(4), pp. 288-296.
57. Hagiwara, M., Henricson, M., Jonsson, A. and Suserud, B. (2011). Decision support tools in pre-hospital care: a systematic review of randomised controlled trials.
58. Snooks, H., Carter, B. and Dale, J. (2014) Support and Assessment for Fall Emergency Referrals (SAFER 1): Cluster Randomised Trial of Computerised Clinical Decision Support for Paramedics, PLOS One.
59. Burns, E. (2016) Scalp Hematoma Characteristics Associated with Intracranial Injury in Paediatric Minor Head Injury. Academic Emergency Medicine, 23(5), pp. 577-583.
60. Araki, T., Yokota, H., and Morita, A. (2017) Paediatric traumatic brain injury: characteristic features, diagnosis, and management, Neurologia medico-chirurgica, 57(2), pp. 82-93.
61. Moritz, D., Ebbs, P. and Carver, H. (2020) Paramedic ethics, capacity and the treatment of vulnerable patients. Journal of Paramedic Practice.

For peer review only - http://bmjopen.bmj.com/site/about/guidelines.xhtml

62. Dayan, P.S., Holmes, J.F., Atabaki, S., Miskin, M. and Kupperman, N. (2014) Association of Traumatic Brain Injuries with Vomiting in Children with Blunt Head Trauma, Annals of Emergency Medicine, 63(6), pp. 657-665.
63. Borland, M.L., et al. (2018) Delayed presentations to emergency departments of children with head injury: a predict study. Annals of Emergency Medicine, 74(1), pp. 1-10.
64. Crowe, L., et al., The epidemiology of paediatric head injuries: data from a referral centre in Victoria, Australia. Journal of paediatrics and child health, 2009. 45(6): p. 346-350.
65. Harper, J.A., Klassen, T.P., Balshaw, R., Dyck, J. and Osmond, M.H. (2020) Characteristics of vomiting as a predictor of intracranial injury in paediatric minor head injury, Canadian Journal of Emergency Medicine, 22(6).
66. Lee, L.K., Monroe, D., Bachman, M.C., Glass, T.F., Mahajan, P.V., Cooper, A., Stanley, R.M., Miskin, M., Dayan, P.S., Holmes, J.F., Kuppermann, N. (2014) Traumatic Brain Injury (TBI) Working Group of Pediatric Emergency Care Applied Research Network (PECARN). Isolated loss of consciousness in children with minor blunt head trauma. JAMA Pediatr, 168(9), pp. 837-43.
67. Lockie et al. (2013) Triggers for head computed tomography following paediatric head injury: Comparison of physicians' reported practice and clinical decision rules, Emergency Medicine Australia, 25, pp. 75-82.
68. Fowler, J., Beovich, B. and Williams, B. (2018) Improving paramedic confidence with paediatric patients: A scoping review. 15(1).
69. Bressan, S., Monagle, P., Dalziel, S., Borland, M., Phillips, N., Kochar, A., Lyttle, M., Cheek, J., Neutze, j., Oakley, E., Dalton, S., Gilhotra, Y., Hearps, S., Furyk, J. and Babl, F. (2020) Risk of traumatic intracranial haemorrhage in children with bleeding disorders, Journal of Paediatrics and Child Health, 56(12), pp. 1839-2003.
70. Pandor, A., Goodacre, S., Harnan, S., Holmes, M., Pickering, A., Fitzgerald, P., Rees, A. and Stevenson, M. (2011) Diagnostic management strategies for adults and children with minor head injury: a systematic review and an economic evaluation, Health Technology Assessment. 15(27).
71. Lee, L., Dayan, P. Gerardi, M., Borgialli, d., Badawy, M., Callahan, J., Lillis, K., Stanley, R., Gorelick, M., Dong, L., Zuspan, S., Holmes, J. and Kupperman, N. (2011) Intracranial haemorrhage after blunt head trauma in children with bleeding disorders, The Journal of Paediatrics, 158(6), pp. 1003-1008.

FIGURE LEGEND:

Figure 1- Prisma flow chart

WORD COUNT (excluding tables and references):

6000

KEYWORDS:

Systematic review; head injuries; paramedic; children.

For peer review only

Figure 1.0 PRISMA flow diagram

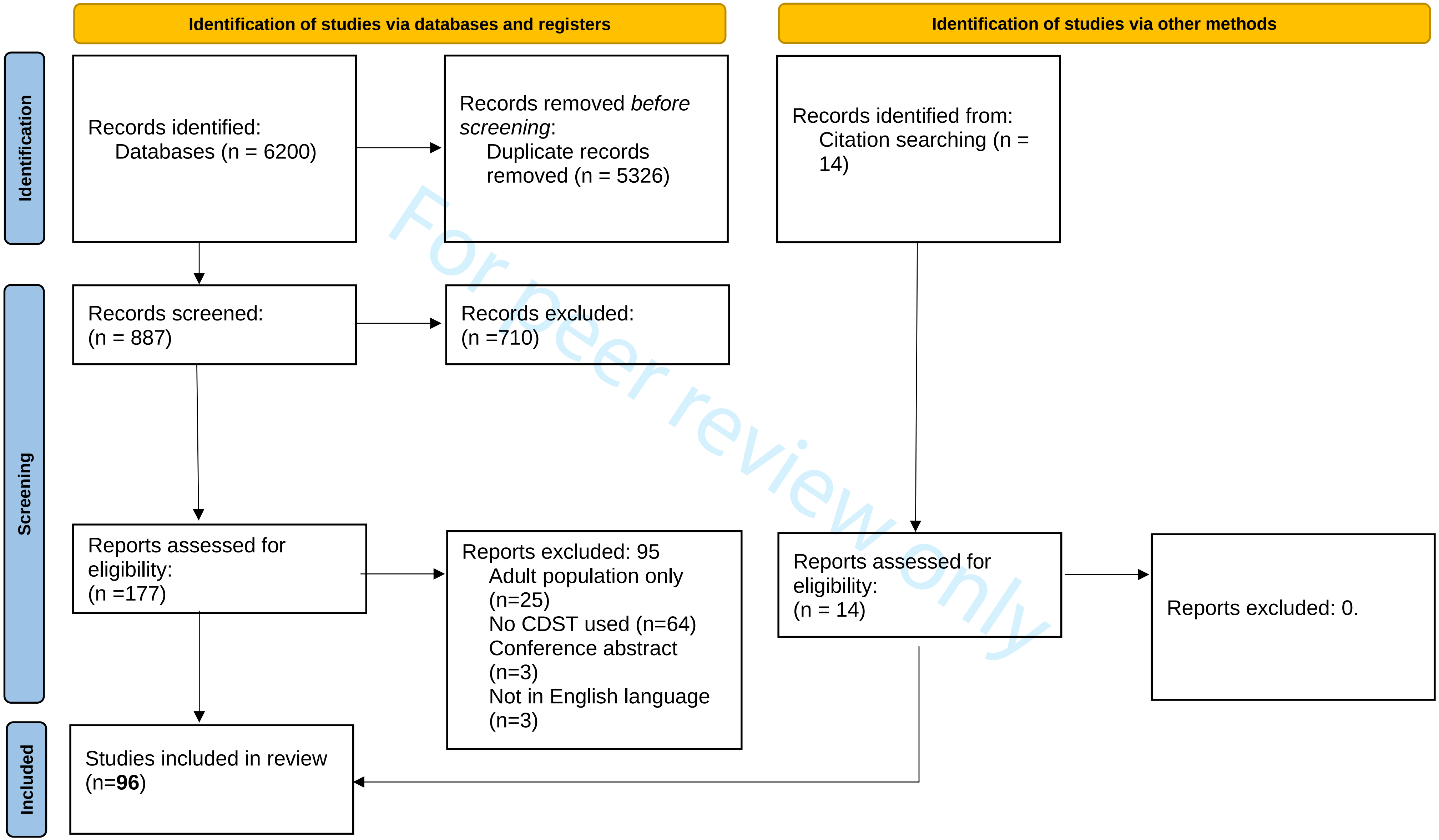

From: Page MJ, McKenzie JE, Bossuyt PM, Boutron I, Hoffmann TC, Mulrow CD, et al. The PRISMA 2020 statement: an updated guideline for reporting systematic reviews. BMJ 2021;372:n71. doi: 10.1136/bmj.n71. For more information, visit: http://www.prisma-statement.org/

#	Query	Limiters/Expanders	Last Run Via	Results
S1	(("Clinical decision support tool" OR "Clinical decision tool" OR "Clinical decision rule" OR "Diagnostic accuracy tool" OR "Triage tool" OR "Hospital-based tool" OR CDRs OR Intervention OR "Clinical decision score" OR "Decision Support Technique*") AND ("Head injury" OR "Minor head injury" OR "Traumatic brain injury" OR "Head trauma" OR "Head wound" OR "Intracranial injury") AND (Children OR Child OR Pediatric OR Paediatric OR Baby OR Babies OR Infant OR Schoolchild* OR Adolescent OR Teenage* OR "Young person")) AND ("emergency department" OR "trauma center" OR ED OR "A&E" OR "accident and emergency" OR "Emergency Service, Hospital")	Search modes - Boolean/Phrase	Interface - EBSCOhost Research Databases<br>Search Screen - Advanced Search<br>Database - CINAHL Plus;AMED - The Allied and Complementary Medicine Database;MEDLINE;APA PsycInfo	895

https://exports.ebscohost.com/sdc/a60c5b63-e8cf-4ea9-909b-1c08e39cb914.zip

Link to full search strategy.

For peer review only

Supplementary Table 1. Categories in each tool

	Signs of Skull fracture	Scalp Haematoma	Vomiting	Neuro Deficit	Headache	LOC	AMS	GCS	Seizure	Severe mechanism of injury	Drowsy	Amnesia	Vertigo	Non accidental injury	Clotting impairment/ anticoagulated	Drug/ alcohol use	Patient alert and well	Age
**PECARN (2-18)**	YES	NO	YES	NO	YES	YES	YES	YES	NO	YES	YES	NO	NO	NO	NO	NO	NO	NO
**PECARN (<2)**	YES	YES	NO	NO	NO	YES	YES	YES	NO	YES	YES	NO	NO	NO	NO	NO	YES	YES
**CHALICE**	YES	YES	YES	YES	NO	YES	NO	YES	YES	YES	YES	YES	NO	YES	NO	NO	NO	NO
**CATCH**	YES	YES	NO	NO	YES	YES	YES	YES	NO	YES	NO	YES	NO	NO	NO	NO	NO	NO
**NEXUS 2**	YES	YES	YES	YES	NO	YES	YES	YES	NO	NO	YES	YES	NO	NO	YES	NO	NO	NO
**HIDATq**	YES	YES	YES	YES	NO	YES	YES	YES	YES	YES	YES	YES	NO	YES	YES	NO	YES	YES
**PALCHACK**	YES	YES	YES	NO	YES	NO	YES	NO	NO	NO	NO	NO	NO	NO	NO	NO	NO	NO
**HAYDEL**	NO	YES	YES	NO	YES	NO	NO	NO	YES	NO	NO	YES	NO	NO	NO	YES	NO	YES
**ATABAKI**	YES	YES	YES	YES	YES	YES	YES	YES	YES	YES	YES	YES	YES	NO	NO	YES	NO	YES
**GREENES**	YES	YES	NO	NO	NO	NO	NO	NO	NO	NO	NO	NO	NO	NO	NO	NO	NO	NO
**KLEMETTI**	YES	YES	NO	YES	NO	YES	YES	NO	NO	NO	NO	NO	YES	NO	NO	NO	NO	NO
**QUAYLE**	YES	NO	NO	YES	NO	YES	YES	NO	YES	NO	NO	NO	NO	NO	NO	NO	NO	NO
**DIETRICH**	NO	NO	YES	YES	YES	YES	NO	NO	YES	NO	NO	NO	NO	NO	NO	NO	NO	NO
**GUZEL**	YES	YES	NO	YES	YES	YES	NO	NO	YES	YES	NO	YES	NO	NO	NO	NO	NO	NO
**PredAHT**	NO	YES	NO	NO	NO	NO	NO	NO	YES	NO	NO	NO	NO	NO	NO	NO	NO	NO
**CHIDA**	YES	NO	NO	NO	NO	NO	NO	YES	NO	NO	NO	NO	NO	NO	NO	NO	NO	NO
**CIDSS2**	YES	NO	NO	YES	NO	NO	NO	YES	YES	NO	NO	NO	NO	NO	NO	YES	NO	NO
**PediBIRN**	YES	YES	NO	NO	NO	NO	NO	NO	NO	NO	NO	NO	NO	NO	NO	NO	NO	NO
**HEAD CT CHOICE**	NO	NO	YES	YES	YES	NO	YES	YES	NO	NO	YES	NO	NO	NO	NO	NO	NO	NO
**SNC GUIDELINE**	YES	YES	YES	YES	YES	YES	YES	YES	YES	NO	NO	NO	NO	NO	YES	NO	NO	NO
**BIG-1**	YES	NO	NO	YES	NO	YES	NO	NO	NO	NO	NO	NO	NO	NO	NO	YES	NO	NO
**NOVEL CDR**	NO	NO	NO	NO	YES	YES	YES	YES	YES	YES	NO	NO	NO	NO	NO	NO	NO	NO
**HEAD TRAUMA EBG**	YES	YES	YES	YES	NO	YES	YES	YES	NO	YES	NO	NO	NO	NO	NO	NO	NO	YES
**DA DALT**	YES	NO	YES	YES	YES	YES	YES	YES	YES	NO	YES	YES	NO	NO	NO	NO	NO	NO
**BUCHANIC**	YES	YES	YES	YES	YES	NO	YES	NO	NO	NO	NO	NO	NO	NO	NO	NO	NO	NO
**KIIDS-TBI CDS**	YES	YES	NO	NO	NO	NO	NO	YES	NO	NO	NO	NO	NO	NO	NO	NO	NO	NO

Supplementary Table 2. Performance accuracy of each tool.

Name of tool	Internal validity	External validity	Implementation/ evaluation	Performance accuracy
**PECARN (under 2)**	Positive evidence	Positive evidence	Mixed evidence supporting potential effect.<br><br>Mixed evidence supporting positive conclusion for implementation.	Sensitivity: 100%<br><br>Specificity: between 53% and 60%
**PECARN (2-18)**	Positive evidence	Positive evidence	Mixed evidence supporting potential effect.<br><br>Mixed evidence supporting positive conclusion for implementation.	Sensitivity: 96.8%<br><br>Specificity: between 53% and 60%
**CHALICE**	Positive evidence	Positive evidence	Mixed evidence supporting positive conclusion for implementation.	Sensitivity: 98%<br><br>Specificity: 86%
**CATCH**	Positive evidence	Positive evidence	Not evaluated for usability, potential effect, or post implementation impact.	Sensitivity: 98%<br><br>Specificity: 50%
**NEXUS II**	Positive evidence	Mixed evidence supporting positive conclusion	Not evaluated for usability, potential effect, or post implementation impact.	Sensitivity: 99%<br><br>Specificity: 15%
**HIDATq**	Positive evidence	Not reported	Not evaluated in a published study after development	Negative predictive value for CT was 100% for intracranial injury. The

For peer review only

Supplementary Table 2. Performance accuracy of each tool.

				positive predictive value of the tool was low.
**PALCHACK**	Positive evidence	Positive evidence	Not evaluated in a published study after development	Sensitivity: 100%<br><br>Specificity: 46%
**HAYDEL**	Positive evidence	Not reported	Not evaluated in a published study after development	Sensitivity: 100%<br><br>Specificity: 24%
**ATABAKI**	Positive evidence	Not reported	Not evaluated in a published study after development	Sensitivity: 100%<br><br>Specificity: 46%
**BUCHANICH**	Positive evidence	Not reported	Not evaluated in a published study after development	Sensitivity: 100%<br><br>Specificity: 40%
**DA DALT**	Developed but not tested for internal validity	Not reported	Not evaluated in a published study after development	Sensitivity: 100%<br><br>Specificity: 87%
**GREENES**	Developed but not tested for internal validity	Not reported	Not evaluated in a published study after development	Sensitivity: 53%<br><br>Specificity: 72%
**KLEMETTI**	Developed but not tested for internal validity	Not reported	Not evaluated in a published study after development	Sensitivity: 94%<br><br>Specificity: 29%
**QUAYLE**	Developed but not tested for internal validity	Not reported	Not evaluated in a published study after development	Sensitivity: 44%<br><br>Specificity: 85%

For peer review only

Supplementary Table 2. Performance accuracy of each tool.

**DIETRICH**	Developed but not tested for internal validity	Not reported	Not evaluated in a published study after development	Sensitivity: 100%<br><br>Specificity: 17%
**GUZEL**	Developed but not tested for internal validity	Not reported	Not evaluated in a published study after development	Sensitivity: 69%<br><br>Specificity: 43%
**PredAHT 2**	Positive evidence	Positive evidence	Not evaluated for usability, potential effect, or post implementation impact.	Sensitivity: 72%<br><br>Specificity: 86%
**CHIDA**	Negative evidence	Not reported	Not evaluated in a published study after development	Sensitivity: 94%<br><br>Specificity: 69%
**CIDSS2**	Positive evidence	Not reported	Not evaluated in a published study after development	Sensitivity: 51%<br><br>Specificity: 96%
**PediBIRN**	Positive evidence	Positive evidence	Not evaluated in a published study after development	Sensitivity: 96%<br><br>Specificity: 29%

For peer review only

Supplementary Table 2. Performance accuracy of each tool.

**Head CT Choice**	Positive evidence	Not reported	Not evaluated in a published study after development	Not reported
**SNC guideline**	Positive evidence	Positive evidence	Not evaluated in a published study after development	Sensitivity: 95%<br><br>Specificity: 59%
**BIG-1**	Positive evidence	Not reported	Not evaluated in a published study after development	Not reported
**Novel simplified CDR**	Positive evidence	Not reported	Not evaluated in a published study after development	Sensitivity: 80%<br><br>Specificity: 51%
**Head trauma EBG algorithm**	Positive evidence	Not reported	Not evaluated in a published study after development	Not reported
**KIIDS-TBI CDS**	Positive evidence	Positive evidence	Not evaluated in a published study after development	Sensitivity: 100%<br><br>Specificity: 26%

For peer review only

Supplementary Table 2. Performance accuracy of each tool.

For peer review only

KIN 4400 Independent Research Study in Kinesiology | PRISMA 2020 Checklist

Section and Topic	Item #	Checklist item	Location where item is reported
**TITLE**			
Title	1	Identify the report as a literature review.	Page 1
**ABSTRACT**			
Abstract	2	Provide a structured summary including, as applicable: background; objectives; data sources; study eligibility criteria, participants, and interventions; study appraisal and synthesis methods; results; limitations; conclusions and implications of key findings.<br>See the PRISMA 2020 for Abstracts checklist for the complete list.	1 and 2
**INTRODUCTION**			
Rationale	3	Describe the rationale for the review in the context of existing knowledge, i.e., what is already known about your topic.	2 and 3
Objectives	4	Provide an explicit statement of the objective(s) or question(s) the review addresses with reference to participants, interventions, comparisons, outcomes, and study design (PICOS).	3
**METHODS**			
Eligibility criteria	5	Specify the inclusion and exclusion criteria for the review and how studies were grouped for the syntheses with study characteristics (e.g., PICOS, length of follow-up) and report characteristics (e.g., years considered, language, publication status) used as criteria for eligibility, giving rationale.	4
Information sources	6	Specify all databases, registers, websites, organisations, reference lists and other sources searched or consulted to identify studies. Specify the date when each source was last searched or consulted.	3
Search strategy	7	Present the full search strategies for all databases, registers and websites, including any filters and limits used.	3,4,5,6
Selection process	8	State the process for selecting studies (i.e., screening, eligibility).<br>Specify the methods used to decide whether a study met the inclusion criteria of the review, including how many reviewers screened each record and each report retrieved, whether they worked independently, and if applicable, details of automation tools used in the process.	3,4,5,6
Study risk of bias assessment	11	Specify the methods used to assess risk of bias in the included studies, including details of the tool(s) used, how many reviewers assessed each study and whether they worked independently, and if applicable, details of automation tools used in the process.	3,4,5
**RESULTS**			
Study selection	16a	Describe the results of the search and selection process, from the number of records identified in the search to the number of studies included in the review, ideally using a flow diagram.	6

The checklist has been adapted for KIN 4400 Independent Research Study in Kinesiology at the University of Guelph-Humber. Last updated: Dec 9, 2021

Adapted From: Page MJ, McKenzie JE, Bossuyt PM, Boutron I, Hoffmann TC, Mulrow CD, et al. The PRISMA 2020 statement: an updated guideline for reporting systematic reviews. BMJ 2021;372:n71. doi: 10.1136/bmj.n71 *Adapted From:* Page MJ, McKenzie JE, Bossuyt PM, Boutron I, Hoffmann TC, Mulrow CD, et al. The PRISMA 2020 statement: an updated guideline for reporting systematic reviews. BMJ 2021;372:n71. doi: 10.1136/bmj.n71

For more information, visit: http://www.prisma-statement.org/

For peer review only - http://bmjopen.bmj.com/site/about/guidelines.xhtml

KIN 4400 Independent Research Study in Kinesiology | PRISMA 2020 Checklist

Section and Topic	Item #	Checklist item	Location where item is reported
	16b	Cite studies that might appear to meet the inclusion criteria, but which were excluded, and explain why they were excluded.	6
Study characteristics	17	Cite each included study and present its characteristics (e.g., study size, PICOS, follow-up period).	6
Risk of bias in studies	18	Present assessments of risk of bias for each included study.	N/A
Results of individual studies	19	For all outcomes, present, for each study: (a) summary statistics for each group (where appropriate) and (b) an effect estimate and its precision (e.g. confidence/credible interval), ideally using structured tables or plots.	6,7,8,9,13,14,15,16
**DISCUSSION**			
Discussion	23a	Provide a general interpretation of the results in the context of other evidence.	20
	23b	Discuss any limitations of the evidence included in the review.	23
	23c	Discuss any limitations of the review processes used.	23
	23d	Discuss implications of the results for practice, policy, and future research.	23
**OTHER INFORMATION**			
Registration and protocol	24a	Provide registration information for the review, including register name and registration number, or state that the review was not registered.	3
	24b	Indicate where the review protocol can be accessed, or state that a protocol was not prepared.	3
	24c	Describe and explain any amendments to information provided at registration or in the protocol.	N/A
Support	25	Describe sources of financial or non-financial support for the review, and the role of the funders or sponsors in the review.	28
Competing interests	26	Declare any competing interests of review authors.	27
Availability of data, code, and other materials	27	Report which of the following are publicly available and where they can be found: template data collection forms; data extracted from included studies; data used for all analyses; analytic code; any other materials used in the review.	n/a

For peer review only

The checklist has been adapted for KIN 4400 Independent Research Study in Kinesiology at the University of Guelph-Humber. Last updated: Dec 9, 2021

Adapted From: Page MJ, McKenzie JE, Bossuyt PM, Boutron I, Hoffmann TC, Mulrow CD, et al. The PRISMA 2020 statement: an updated guideline for reporting systematic reviews. BMJ 2021;372:n71. doi: 10.1136/bmj.n71 *Adapted From:* Page MJ, McKenzie JE, Bossuyt PM, Boutron I, Hoffmann TC, Mulrow CD, et al. The PRISMA 2020 statement: an updated guideline for reporting systematic reviews. BMJ 2021;372:n71. doi: 10.1136/bmj.n71

For more information, visit: http://www.prisma-statement.org/

For peer review only - http://bmjopen.bmj.com/site/about/guidelines.xhtml
